# Identification of novel antifungal agents: antimicrobial evaluation, SAR, ADME–Tox and molecular docking studies of a series of imidazole derivatives

**DOI:** 10.1186/s13065-019-0623-6

**Published:** 2019-08-06

**Authors:** Btissam Bouchal, Farid Abrigach, Abdelilah Takfaoui, Manal Elidrissi Errahhali, Mounia Elidrissi Errahhali, Pierre H. Dixneuf, Henri Doucet, Rachid Touzani, Mohammed Bellaoui

**Affiliations:** 1 Genetics Unit, Faculty of Medicine and Pharmacy of Oujda, University Mohammed Premier, Oujda, Morocco; 2 Laboratory of Applied Chemistry & Environment, Faculty of Sciences, University Premier, Oujda, Morocco Mohamed Premier, Oujda, Morocco; 3 Institut des Sciences Chimiques de Rennes, UMR 6226, CNRS, Université de Rennes, Rennes, France

**Keywords:** Imidazole, Antifungal, Antibacterial, Structure–activity relationship, ADME–Tox, Docking

## Abstract

Thirty-four imidazole-based compounds synthesized by one-pot catalytic method were evaluated for their antifungal and antibacterial activities against several fungal and bacterial strains. None of the compounds had antibacterial activity. Interestingly, compounds **1**, **2**, **3**, **10** and **15** displayed a strong antifungal activity against all the tested fungal species, while compounds **5**, **7**, **9**, **11**, **21** and **27** showed a moderate antifungal activity. To better understand the biological activity of the most active compounds ADME–Tox and molecular docking studies were carried out. Interestingly, compounds **1**, **2**, **3**, **7**, **10** and **15** showed excellent bioavailability. In addition, compounds **1**, **2** and **3**, exhibited good toxicity profiles. Docking studies of the two most active compounds **2** (IC_50_ of 95 ± 7.07 μM) and **10** (IC_50_ of 235 ± 7.07 μM) suggested that they might act by inhibiting the fungal lanosterol 14α-demethylase. Therefore, these novel antifungal agents merit further characterization for the development of new antifungal therapeutics.

**Electronic supplementary material:**

The online version of this article (10.1186/s13065-019-0623-6) contains supplementary material, which is available to authorized users.

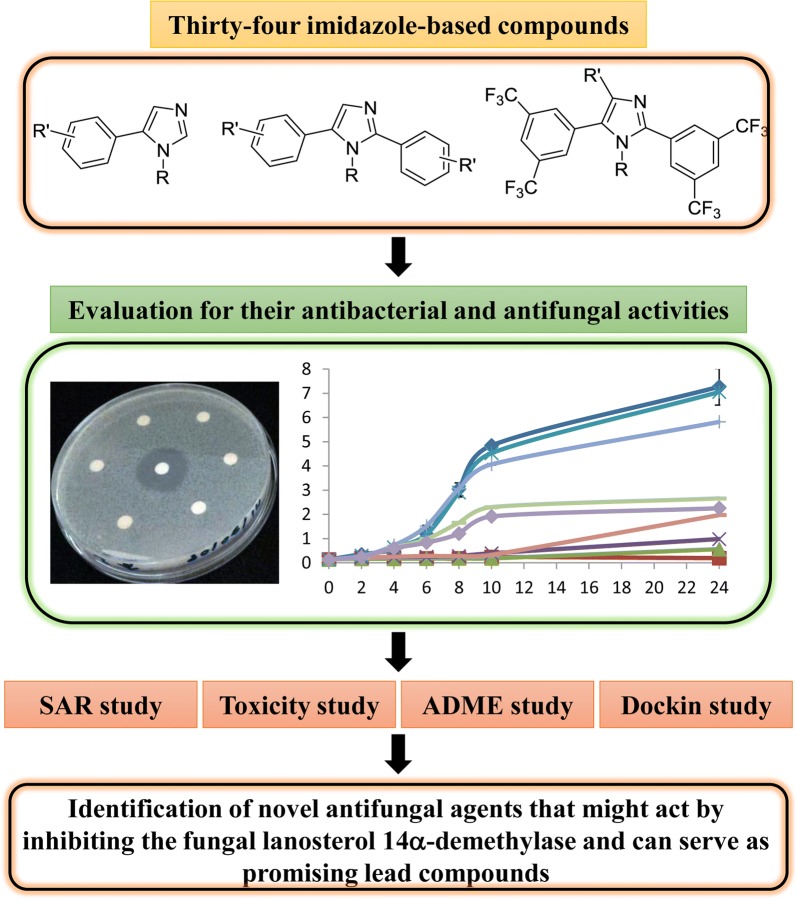

## Introduction

Human infectious diseases are among the top ten causes of death worldwide, according to the World Health Organization [1]. Therefore, infectious diseases are among the most important public health problems around the world, especially in developing countries [2]. Drug-resistant infections are also a growing public health threat worldwide [3–5]. As a matter of fact, the increasing number of antifungal drug resistance has become more frequent and serious, creating a need for safer and more effective antifungal therapies [6–9]. Indeed, increasing resistance of fungal pathogens to current antifungal drugs is one of the reasons for the difficulty to tackle fungal infections, particularly in immune-compromised individuals [10]. Given this difficulty in controlling fungal infections, a new class of antifungal drugs with novel mechanisms of action and broad spectrum of activity must be discovered.

In this view, many heterocyclic compounds have been studied in order to discover novel antimicrobial agents. In fact, heterocyclic chemistry has grown considerably and more than 90% of new drugs contain heterocycles [11]. More particularly, imidazole-based heterocyclic compounds occupy a prominent place in heterocyclic chemistry [12, 13]. Members of this family of compounds have diverse biological, pharmacological, environmental and industrial applications [14]. Indeed, this type of compounds act as inhibitors of p38 MAP kinase and B-Raf [15], and showed anti-inflammatory, anti-cancer, antifungal, anti-tuberculosis, and anti-diabetic activities [16–18]. Accordingly, several imidazole-based heterocyclic compounds have been clinically used to treat many diseases such as Bifonazole which is a clinically used antifungal agent with a broad spectrum of activity [19, 20], Metronidazole used to treat a wide variety of bacterial and parasitic infections [21], Cimetidine used in the treatment of duodenal and gastric ulcers [22], and Eprosartan used as angiotensin II inhibitor and antihypertensive agent [23].

In this context, we sought to study the antifungal and antibacterial biological activities of a series of 34 imidazole-based heterocyclic compounds that have been synthesized by a simple one-pot catalytic method. We have thus identified five imidazole derivatives **1**, **2**, **3**, **10** and **15**, which act specifically as potent antifungal agents and lack antibacterial activity. Their structure-activity relationship analysis (SAR), ADME–Tox profiles, and molecular docking studies were carried out to understand their biological activity. These novel antifungal agents merit further characterization and can serve as a promising lead compounds for the discovery of new antifungal therapeutics.

## Results and discussion

**Fig. 1:**
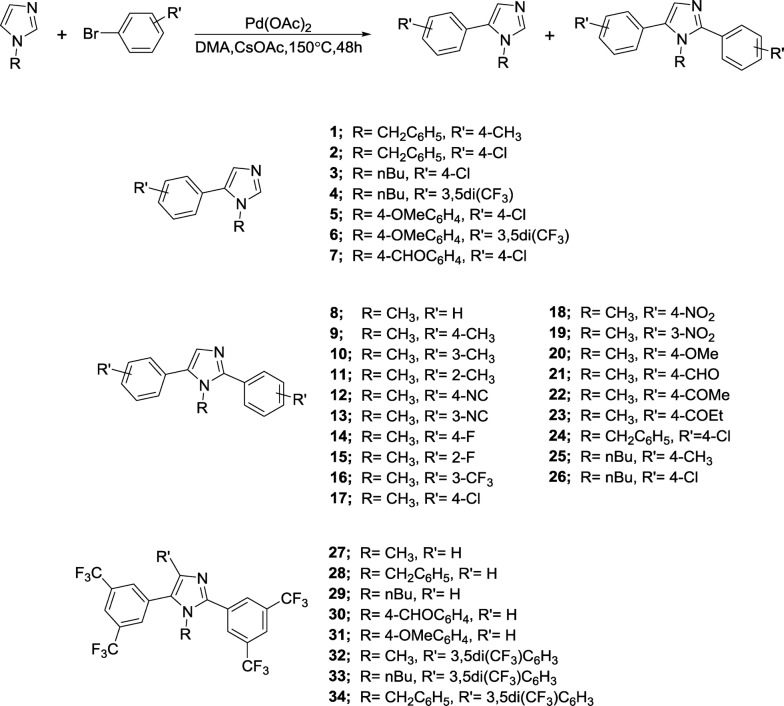
Synthetic route and structure of the molecules studied in this paper

The imidazole-based compounds studied in this paper were synthesized by one-pot catalytic method [24] and are shown in Fig. 1. The spectroscopic information of these compounds is described below in materials and methods.

### Antifungal and antibacterial activities of imidazole derivatives

**Fig. 2:**
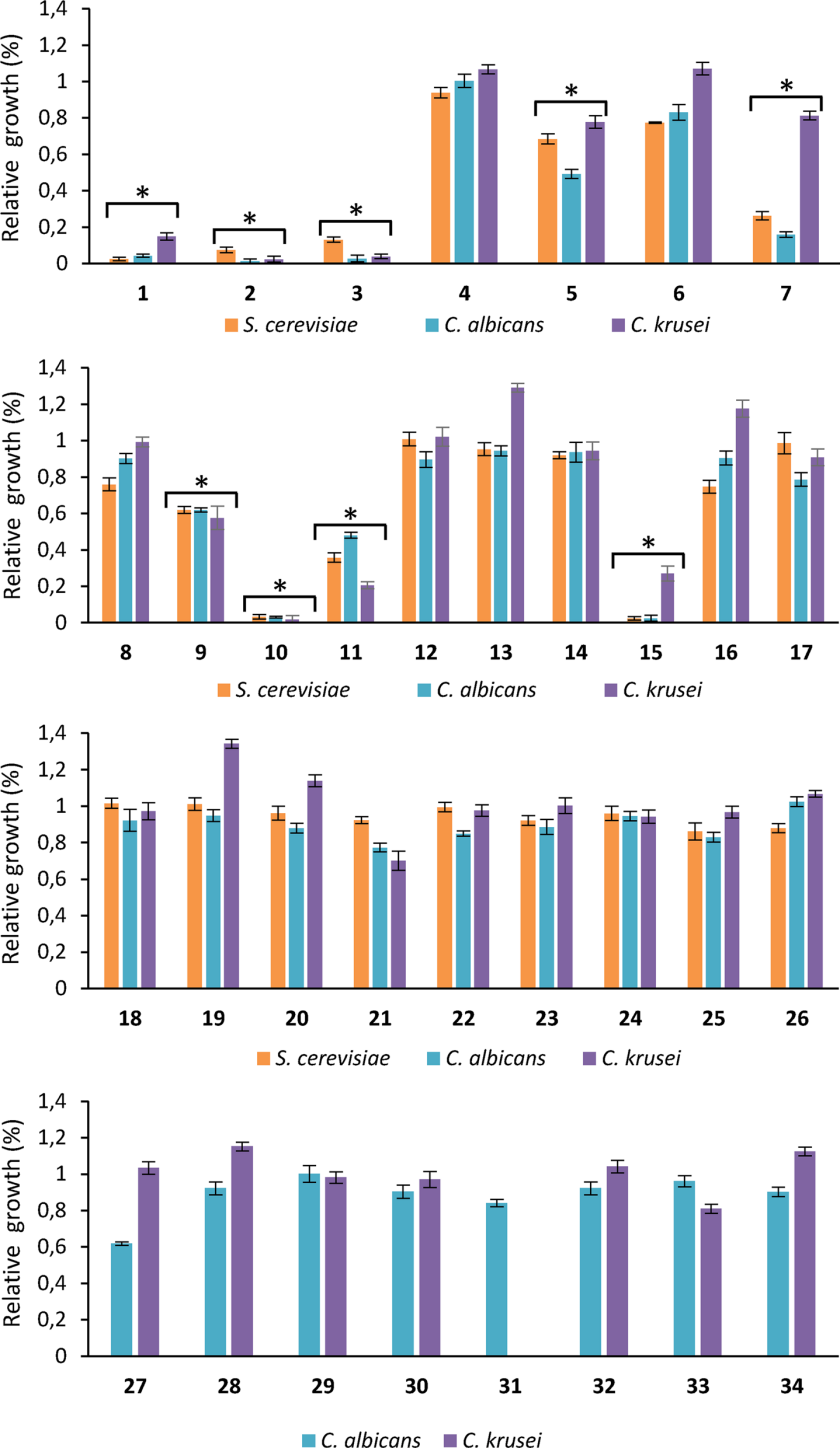
Antifungal activity of the studied compounds against *Saccharomyces cerevisiae*, *Candida albicans* and *Candida krusei*. Cells were cultured in the presence of 500 µM of each compound for 24 h and growth rate was then assayed by the OD_600_. Growth in the presence of compound was expressed as a percentage relative to the untreated control. All experiments were carried out in triplicate and means were calculated ± SD. *p < 0.05 versus untreated control

We first evaluated the antifungal activity of our imidazole derivatives against three fungal species (*S. cerevisiae*, *C. albicans* and *C. krusei*) as described in materials and methods. All the compounds were used at 500 μM. Interestingly, compounds **1**, **2**, **3**, **10** and **15** displayed strong antifungal activity (greater than 80% growth inhibition) against all three tested fungal species, whereas compounds **5**, **7**, **9**, **11**, **21** and **27** showed moderate antifungal activity (20–50% growth inhibition) (Fig. 2). On the other hand, compounds, **6**, **8**, **16**, **17**, **25**, **31** exhibited weak antifungal activity (5–20% growth inhibition), while the rest of the molecules were not toxic to yeast cells (Fig. 2).

Regarding the antibacterial activity, all the compounds were tested for toxicity against three gram-negative bacterial strains (*E. coli*, *C. freundii* and *S. braenderup*) and two gram-positive bacterial strains (*L. monocytogenes* and *S. aureus*). All the compounds showed no antibacterial activity against all bacterial strains used. Therefore, to better understand the antifungal activity of the most active compounds, SAR, ADME–Tox and molecular docking studies were carried out and are detailed below.

### SAR of imidazole derivatives

The SAR analysis of the mono-arylated series (**1**–**7**) revealed that the antifungal activity of these compounds depends essentially on the attached group (R) at the position 1 of imidazole moiety and the attached group (R’) of the phenyl ring. Investigation of R moiety in compounds **2**, **3**, **5** and **7**, showed that the presence of OMeC_6_H_4_ substituent in compound **5** or CHOC_6_H_4_ group in compound **7** resulted in moderate antifungal activity. Whereas, the presence of benzyl substituent in compound **2** or *n*-Butyl group in compound **3** resulted in high antifungal activity. Further evaluation of compounds **2** and **3** demonstrated that **2** was more potent than **3**, with a half-maximal inhibitory concentration (IC_50_) against *S. cerevisiae* of 95 ± 7.07 μM and 220 ± 14.14 μM, respectively (Table 1).

**Table 1:** IC_50_ against *S. cerevisiae* of the imidazole derivatives with strong antifungal activity

Compd.	Structure	IC_50_ (μM)^a^
**1**		240 ± 14.14
**2**		95 ± 7.07
**3**		220 ± 14.14
**10**		235 ± 7.07
**15**		305 ± 21.21

^a^The value obtained for each compound represents the mean of three independent experiments ± SD

These findings suggest that the small size and the electron withdrawing character of the substituent at the position 1 of imidazole moiety are important for the antifungal activity of these compounds. Regarding the comparison between compounds **1** and **2**, we observed a stronger antifungal activity against *S. cerevisiae* with **2** (IC_50_ of 95 ± 7.07 μM) compared to **1** (IC_50_ of 240 ± 14.14 μM), suggesting that the replacement of chlorine (an electron withdrawing group) with methyl (an electron donating group) decreases the antifungal potential. Together, these data suggest that the introduction of electron withdrawing substituents in R and R’ increases the antifungal activity of these compounds.

The SAR analysis of the 2, 5-diarylated imidazole derivatives series (**8**–**26**) revealed that the introduction of electron withdrawing or electron donating groups into the phenyl rings at the *para* position resulted in moderate antifungal activity (**9**, **21**) or no activity (**12**, **14**, **17**, **18**, **20**, **22** and **23**). We also observed that the presence of electron withdrawing groups [CF_3_ (**16**), CN (**13**) or NO_2_ (**19**)] into the phenyl rings at the *meta* position resulted in loss of antifungal activity. However, the presence of an electron donating group (methyl group) at the *meta* position of the phenyl rings (**10**) resulted in strong antifungal activity. We also found that the substitution of the phenyl rings at the *ortho* position with Fluor or methyl groups led to strong antifungal activity (**15**) or moderate activity (**11**). Further evaluation of the most active compounds of this series (**10** and **15**) has demonstrated that compound **10** was more potent than compound **15**, with IC_50_’s against *S. cerevisiae* of 235 ± 7.07 μM and 305 ± 21.21 μM, respectively (Table 1). Together, these findings suggest that the antifungal activity of these diarylated imidazole derivatives depends on the size (small or bulky), position (*para*, *orth*o or *meta*) and electronic effect (withdrawing or donating) of the substituent at the phenyl rings.

The SAR studies of the imidazole derivatives series (**27** to **34**) revealed that the presence of many strong electron withdrawing CF_3_ groups at the *meta* position of phenyl groups did not lead to any antifungal activity. Similarly, the introduction of bulky groups at the position 1 of imidazole moiety did not lead to significant growth inhibition against the tested fungal strains. These findings are consistent with the above observations which suggest that the presence of bulky substituent at the position 1 of imidazole moiety, or at the *meta* position of phenyl group is unfavorable for the antifungal activity.

### ADME–Tox predictions

Currently, the computational predictions of pharmacokinetic and pharmacodynamic parameters such as absorption, distribution, metabolism excretion (ADME) and toxicity risks (Tox) are of great importance in the drug discovery process [25]. Therefore, in this study we used Molinspiration and DataWarroir programs, as described in Materials and Methods, to determine the ADME–Tox profiles of the most active compounds (**1**, **2**, **3**, **7**, **10**, **11** and **15**) as well as the reference antifungal drug Fluconazole.

As shown in Table 2, in silico prediction of toxicity properties (Mutagenic; Tumorigenic; Irritant; Reproductive effect) of our imidazole derivatives revealed that compounds **1**, **2** and **3** have a good toxicological profile with no risk of mutagenicity, tumorigenicity, irritation or reproduction. These Tox properties are similar to those of Fluconazole. However, compound **7** showed a high toxic effect on reproduction probably due to the presence of the C=O carbonyl group at the aldehyde moiety. Regarding 2, 5-diarylated imidazole derivatives **10**, **11** and **15**, toxicity prediction revealed that these compounds have a low mutagenic effect, but without risk of tumorigenicity, irritation or reproduction. Overall, these Tox studies suggest that compounds **1**, **2** and **3** do not have any undesirable moieties involved in toxicity problems, while compounds **10**, **11** and **15** exhibit acceptable toxicity profiles with a low mutagenic effect.

**Table 2:** Toxicity risks and physicochemical properties of the imidazole derivatives with good antifungal activity and the reference drug Fluconazole

Compound	Toxicity risks				Physicochemical properties					
	MU	TU	IR	RE	Log*P*	MW	*n*OH	*n*OHNH	*n*rotb	TPSA (Å^2^)
**1**	None	None	None	None	3.88	248.33	2	0	3	17.83
**2**	None	None	None	None	3.97	268.75	2	0	3	17.83
**3**	None	None	None	None	3.57	234.73	2	0	4	17.83
**7**	None	None	None	*High*	3.62	282.73	3	0	3	34.90
**10**	*Low*	None	None	None	4.70	262.36	2	0	2	17.83
**11**	*Low*	None	None	None	4.70	262.36	2	0	2	17.83
**15**	*Low*	None	None	None	3.96	270.28	2	0	2	17.83
Fluconazole	None	None	None	None	0.56	306.28	7	1	5	81.66

MU, mutagenic; TU, tumorigenic; IR, irritant; RE, reproductive effect; Log*P*, octanol/water partition coefficient characterizing lipophilicity; MW, molecular weight expressed in Daltons; *n*OH, number of hydrogen bond acceptors; *n*OHNH, number of hydrogen bond donors; *n*rotb, number of rotatable bonds; TPSA, total polar surface area

As stated above, Molinspiration was used to determine pharmacokinetic parameters of the most active compounds. Therefore, the following ADME characteristics were calculated: *n*-octanol/water partition coefficient characterizing lipophilicity (Log*P*), molecular weight expressed in Daltons (MW), number of hydrogen bond acceptors (*n*OH), number of hydrogen bond donors (*n*OHNH), number of rotatable bonds (*n*rotb), and total polar surface area (TPSA). As shown in Table 2, all the studied compounds exhibit ADME characteristics that are in agreement with Lipinski’s rule of five, which evaluate the drug-likeness, absorption and intestinal permeability of a compound [26, 27]. Indeed, for all the compounds Log*P* are less than 5.0, MW are less than 500, *n*OH are less than 10, *n*OHNH are less than 5, *n*rotb are less than 10 and TPSA are less than 140 Å^2^. Together, these data suggest that these studied compounds present good bioavailability and therefore can be qualified as a good lead.

### Molecular docking studies

Fungal lanosterol 14α-demethylase (CYP51) is an attractive therapeutic target for the development of antifungal drugs [28, 29]. This enzyme catalyzes an essential step in the synthesis of ergosterol which is an essential component of fungal cell membrane. CYP51 is the target of azoles, the most popular class of antifungal drugs. There has been a considerable amount of research interest into this enzyme and the azoles because of the dramatically increasing number of drug resistance among certain fungal species [30]. Ketoconazole is an antifungal imidazole that belongs to the azole class and it is currently used to treat a wide variety of fungal infections. Like other azoles, it acts by inhibiting selectively [31]. However, the use of Ketoconazole has been limited because it is associated with clinically important toxic side effects [32, 33]. Therefore, it is important to discover novel antifungal imidazoles which act by inhibiting selectively CYP51 without toxic side effects.

Considering the fact that our compounds have imidazole moiety we sought to study the possible binding of our most potent compounds to CYP51 as a possible target protein. Therefore, molecular docking studies were conducted in order to explore the affinity of our most potent imidazole derivatives towards CYP51 from *S. cerevisiae*. Molecular docking is a very powerful computational method for predicting and modeling the interactions between a small molecule and a protein target at the atomic level. Indeed, molecular docking is an important tool, which is widely used in drug design [34, 35].

**Fig. 3:**
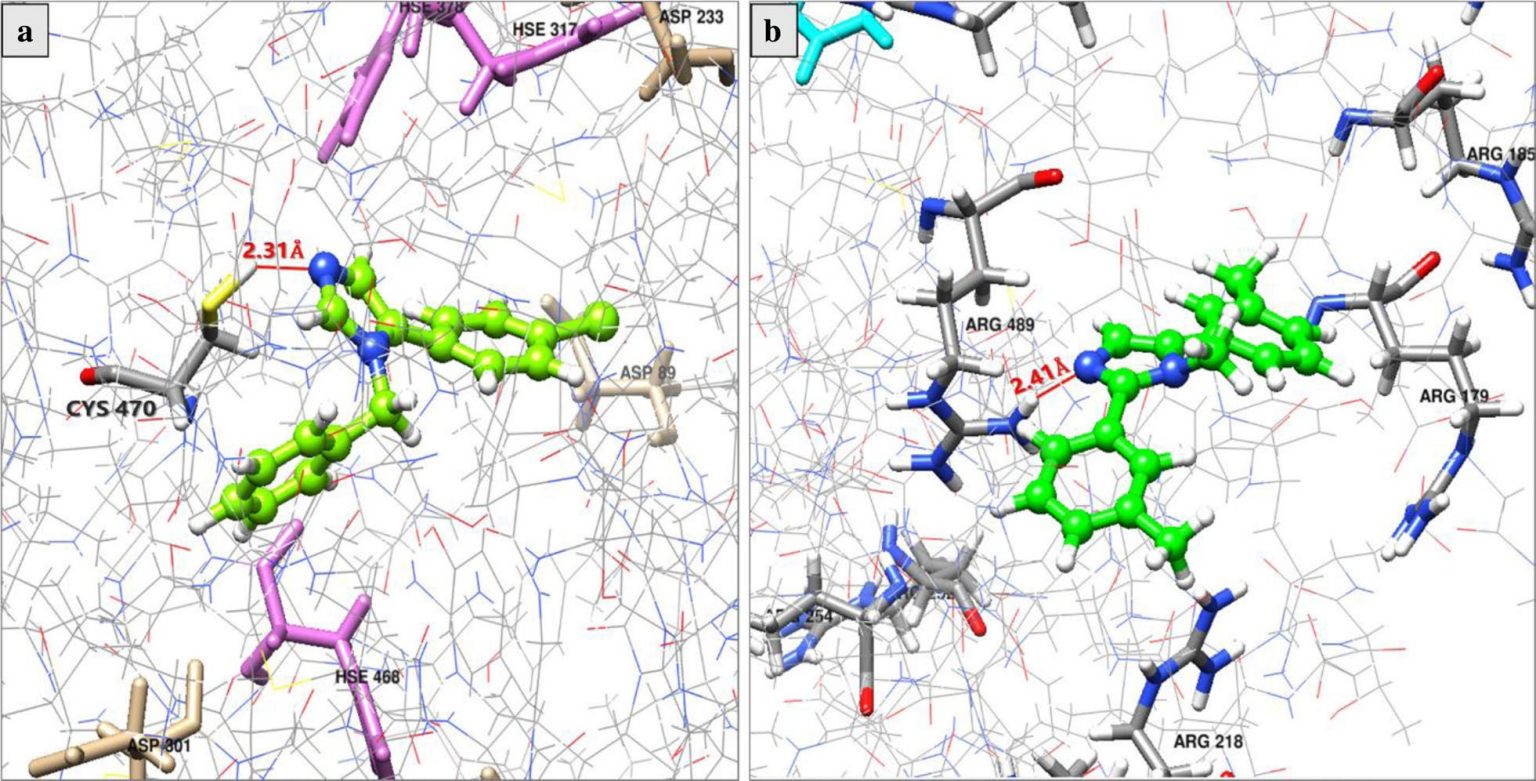
Binding mode of compounds **2** (**a**) and **10** (**b**) with target enzyme CYP51 from *S. cerevisiae*

Table 3 and Fig. 3 show the docking analysis results of compound **2** (the most potent compounds of the mono-arylated series), compound **10** (the most potent compounds of the 2, 5-diarylated imidazole derivatives series), and the antifungal reference drug fluconazole towards CYP51 from *S. cerevisiae*.

**Table 3:** Docking analysis of some imidazole derivatives and the reference drug Fluconazole against *Saccharomyces cerevisiae* CYP51

Compound	ΔG_binding_ (kcal/mol)	H-bond	Distance (Å)
**2**	− 6.857	N(3)-Cys470	2.31
**10**	− 6.791	N(3)-Arg489	2.41
**6**	− 5.616	–	–
**30**	–	–	–
Fluconazole	− 7.337	N(4)-Arg467	2.70

Three parameters were used for docking analysis: the binding affinity expressed in kcal/mol, the interactions between ligand atoms and amino acid residues of the target protein, and the distance of these interactions. Therefore, our results of docking showed that the two tested imidazole derivatives (**2** and **10)** and fluconazole perform a hydrogen bond interaction (H-bond) between one of their nitrogen atoms and an amino acid residue of the target protein. For fluconazole, nitrogen atom in position 4 makes one H-bond with Arg467 amino acid of the target protein at a bond distance of 2.70 Å and a binding affinity of − 7.337 kcal/mol. While for compound **2**, nitrogen atom in position 3 makes one H-bond with Cys470 amino acid of the target protein at a bond distance of 2.31 Å and a binding affinity of − 6.857 kcal/mol. Similarly, nitrogen atom in position 3 of compound **10** makes one H-bond, but with Arg489 amino acid of the target protein with a bond distance of 2.41 Å and a binding affinity of − 6.791 kcal/mol.

To affirm correlation between in silico prediction of binding affinity to CYP51 and antifungal activity, we also docked two imidazole derivatives which lack antifungal activity (**6** and **30**) against CYP51 (Table 3). Interestingly, our docking studies revealed that compound **30** does not have binding affinity toward CYP51. Regarding compound **6**, the results of docking indicated that this compound have lower binding affinity (− 5.616 kcal/mol) toward CYP51 as compared to the active compounds **2** and **10** (− 6.857 and − 6.791 kcal/mol). Moreover, the docking result revealed that, like compound **30**, compound **6** does not interact with CYP51 by hydrogen bond interaction. Together, these docking studies agreed with the antifungal activity and suggest that the two most active compounds **2** and **10** have good binding affinity with target enzyme CYP51 and therefore might act by inhibiting the fungal lanosterol 14α-demethylase.

## Conclusion

In this paper we investigated the antifungal and antibacterial biological activities of thirty-four imidazole-based compounds synthesized by one-pot catalytic method. Antifungal activity was assayed against five fungal species, while antibacterial activity was tested against five bacterial species. None of the tested compounds showed an antibacterial activity. Interestingly, compounds **1**, **2**, **3**, **10** and **15** displayed a strong antifungal activity against all the tested fungal species, while compounds **5**, **7**, **9**, **11**, **21** and **27** showed a moderate antifungal activity. SAR studies revealed that the antifungal activity of these imidazole derivatives depends on the size (small or bulky), position (*para*, *orth*o or *meta*) and electronic effect (withdrawing or donating) of the substituents at the phenyl rings, as well as the bulkiness and the electronic effect of the substituent in position 1 of the imidazole moiety. ADME analysis showed that compounds **1**, **2**, **3**, **7**, **10** and **15** have excellent bioavailability. In addition, Tox studies showed that compounds **1**, **2** and **3**, have good toxicity profiles, whereas **10**, **11** and **15** have low mutagenic effect. On the other hand, compound **7** is likely to cause toxicity because of the high toxic effect on reproduction. Docking studies of the two most active compounds **2** and **10** suggested that they might act by inhibiting the fungal lanosterol 14α-demethylase. Therefore, these novel antifungal agents merit further characterization and can serve as promising lead compounds for the discovery of new antifungal therapeutics.

## Materials and methods

### Chemistry

The 34 imidazole-based molecules analyzed in this paper have been synthesized by a simple one-pot catalytic method [24]. The spectroscopic information of these compounds is as follows:

#### *1*-*Benzyl*-*5*-*p*-*tolyl*-*1H*-*imidazole (****1****)*

31% yield. ^1^H NMR (400 MHz, CDCl_3_) δ 7.48 (s, 1H), 7.30–7.15 (m, 3H), 7.10 (s, 4H), 7.04 (s, 1H), 6.95 (d, *J *= 8.0 Hz, 2H), 5.06 (s, 2H), 2.29 (s, 3H).^13^C NMR (100 MHz, CDCl_3_) δ 138.4, 138.0, 136.9, 133.5, 129.4, 128.9, 128.8, 128.0, 127.9, 126.8, 126.7, 48.7, 21.2.

#### *1*-*benzyl*-*5*-*(4*-*chlorophenyl)*-*1H*-*imidazole (****2****)*

44% yield. ^1^H NMR (400 MHz, CDCl_3_) δ 7.51 (s, 1H), 7.28–7.15 (m, 5H), 7.12 (d, *J *= 8.4 Hz, 2H), 7.06 (s, 1H), 6.92 (d, *J *= 8.4 Hz, 2H), 5.06 (s, 2H). ^13^C NMR (100 MHz, CDCl_3_) δ 139.0, 136.5, 134.2, 132.3, 130.1, 129.0, 128.9, 128.6, 128.2, 128.1,126.5, 48.8.

#### *1*-*butyl*-*5*-*(4*-*chlorophenyl)*-*1H*-*imidazole (****3****)*

45% yield. ^1^H NMR (400 MHz, CDCl_3_) δ 7.50 (s, 1H), 7.34 (d, *J *= 8.4 Hz, 2H), 7.23 (d, *J *= 8.4 Hz, 2H), 6.98 (s, 1H), 3.87 (t, *J *= 7.5 Hz, 2H), 1.53 (quint., *J *= 7.5 Hz, 2H), 1.16 (sext., *J *= 7.5 Hz, 2H), 0.77 (t, *J *= 7.5 Hz, 3H). ^13^C NMR (100 MHz, CDCl_3_) δ 138.3, 134.0, 131.7, 130.0, 129.0, 128.7, 128.3, 45.1, 32.9, 19.6, 13.4.

#### *5*-*(3,5*-*bis(trifluoromethyl)phenyl)*-*1*-*butyl*-*1H*-*imidazole (****4****)*

The product was obtained as trace observed by GC/MS analysis of the crude mixture.

#### *5*-*(4*-*chlorophenyl)*-*1*-*(4*-*methoxyphenyl)*-*1H*-*imidazole (****5****)*

62% yield. ^1^H NMR (400 MHz, CDCl_3_) δ 7.57 (s, 1H), 7.16 (d, *J *= 8.4 Hz, 2H), 7.13 (s, 1H), 7.02 (d, *J *= 8.4 Hz, 2H), 6.99 (d, *J *= 8.4 Hz, 2H), 6.83 (d, *J *= 8.4 Hz, 2H), 3.76 (s, 3H). ^13^C NMR (100 MHz, CDCl_3_) δ 159.4, 139.4, 133.4, 132.1, 129.3, 129.2, 128.8, 128.7, 128.0, 127.0, 114.7, 55.5. C_16_H_13_ClN_2_O (284.74): Calcd C 67.49, H 4.60, N 9.84; Found C 67.28, H 4.37, N 10.08.

#### *5*-*(3,5*-*bis(trifluoromethyl)phenyl)*-*1*-*(4*-*methoxyphenyl)*-*1H*-*imidazole (****6****)*

75% yield. ^1^H NMR (400 MHz, CDCl_3_) δ 7.62 (s, 1H), 7.62 (s, 1H), 7.46 (s, 2H), 7.35 (s, 1H), 7.05 (d, *J *= 8.4 Hz, 2H), 6.88 (d, *J *= 8.4 Hz, 2H), 3.76 (s, 3H).^13^C NMR (100 MHz, CDCl_3_) δ 160.0, 140.4, 131.7 (q, *J *= 34.0 Hz), 130.4, 130.1, 128.4, 127.3 (m), 127.1, 123.0 (q, *J *= 272.7 Hz), 120.6 (m), 115.0, 55.6. C_18_H_12_F_6_N_2_O (386.29): Calcd C 55.97, H 3.13, N 7.25; Found C 55.79, H 3.20, N 7.41.

#### *4*-*(5*-*(4*-*chlorophenyl)*-*1H*-*imidazol*-*1*-*yl)benzaldehyde (****7****)*

53% yield. ^1^H NMR (400 MHz, CDCl_3_) δ 9.97 (s, 1H), 7.87 (d, *J *= 8.4 Hz, 2H), 7.70 (s, 1H), 7.27 (d, *J *= 8.4 Hz, 2H), 7.22 (s, 1H), 7.20 (d, *J *= 8.4 Hz, 2H), 6.99 (d, *J *= 8.4 Hz, 2H).^13^C NMR (100 MHz, CDCl_3_) δ 190.7, 141.1, 135.6, 134.0, 131.0, 130.0, 129.4, 129.0, 127.4, 125.7. C_16_H_11_ClN_2_O (282.72): Calcd C 67.97, H 3.92, N 9.91; Found C 67.75, H 3.97, N 9.72.

#### *1*-*methyl*-*2,5*-*diphenyl*-*1H*-*imidazole (****8****)*

80% yield. ^1^H NMR (400 MHz, CDCl_3_)δ 7.63 (d, *J *= 8.4 Hz, 2H), 7.45–7.25 (m, 8H), 7.14 (s, 1H), 3.62 (s, 3H).

#### *1*-*methyl*-*2,5*-*di*-*p*-*tolyl*-*1H*-*imidazole (****9****)*

81% yield. ^1^H NMR (400 MHz, CDCl_3_) δ 7.52 (d, *J *= 8.4 Hz, 2H), 7.27 (d, *J *= 8.4 Hz, 4H), 7.23–7.17 (m, 4H), 7.10 (s, 1H), 3.58 (s, 3H), 2.34 (s, 6H). ^13^C NMR (100 MHz, CDCl_3_) δ 148.9, 139.0, 138.0, 135.3, 129.5, 129.3, 128.8, 128.7, 127.4, 127.1, 126.2, 33.8, 21.4, 21.3. C_18_H_18_N_2_ (262.35): Calcd C 82.41, H 6.92; Found C 82.50, H 6.98.

#### *1*-*methyl*-*2,5*-*di*-*m*-*tolyl*-*1H*-*imidazole (****10****)*

79% yield. ^1^H NMR (400 MHz, CDCl_3_) δ 7.50 (s, 1H), 7.39 (d, *J *= 8.4 Hz, 1H), 7.30 (d, *J *= 8.4 Hz, 1H), 7.26 (d, *J *= 8.4 Hz, 1H), 7.22–7.10 (m, 5H), 3.59 (s, 3H), 2.35 (s, 3H), 2.34 (s, 3H). ^13^C NMR (100 MHz, CDCl_3_) δ 174.1, 149.1, 138.5, 138.4, 135.5, 130.3, 130.0, 129.7, 129.5, 128.8, 128.7, 128.4, 126.7, 125.8, 125.7, 33.8, 21.5, 21.4. C_18_H_18_N_2_ (262.35): Calcd C 82.41, H 6.92; Found C 82.27, H 6.90.

#### *1*-*methyl*-*2,5*-*di*-*o*-*tolyl*-*1H*-*imidazole (****11****)*

54% yield. ^1^H NMR (400 MHz, CDCl_3_) δ 7.38–7.15 (m, 8H), 7.03 (s, 1H), 3.11 (s, 3H), 2.21 (s, 3H), 2.18 (s, 3H). ^13^C NMR (100 MHz, CDCl_3_) δ 147.7, 138.2, 132.4, 131.3, 130.6, 130.5, 130.4, 130.3, 129.7, 129.4, 129.0, 126.7, 125.9, 125.8, 31.5, 20.0, 19.7. C_18_H_18_N_2_ (262.35): Calcd C 82.41, H 6.92; Found C 82.50, H 6.98.

#### *4,4′*-*(1*-*methyl*-*1H*-*imidazole*-*2,5*-*diyle)benzonitrile (****12****)*

60% yield. ^1^H NMR (400 MHz, CDCl_3_) δ 7.83 (d, *J *= 8.4 Hz, 2H), 7.73 (d, *J *= 8.4 Hz, 4H), 7.55 (d, *J *= 8.4 Hz, 2H), 7.28 (s, 1H), 3.70 (s, 3H). ^13^C NMR (100 MHz, CDCl_3_) δ 148.6, 134.9, 134.1, 133.9, 132.8, 132.6, 129.5, 129.3, 128.8, 118.4, 118.3, 112.9, 112.0, 34.4. C_18_H_12_N_4_ (284.31): Calcd C 76.04, H 4.25; Found C 76.18, H 4.08.

#### *3,3′*-*(1*-*methyl*-*1H*-*imidazole*-*2,5*-*diyle)benzonitrile (****13****)*

60% yield. ^1^H NMR (400 MHz, CDCl_3_) δ 7.99 (s, 1H), 7.98 (d, *J *= 8.4 Hz, 1H), 7.75–7.65 (m, 4H), 7.61 (d, *J *= 8.4 Hz, 1H), 7.57 (d, *J *= 8.4 Hz, 1H), 7.23 (s, 1H), 3.68 (s, 3H). ^13^C NMR (100 MHz, CDCl_3_) δ 146.8, 134.1, 133.4, 133.2, 133.1, 132.4, 132.3, 132.1, 130.2, 130.1, 130.0, 118.0, 117.9, 113.6, 113.3, 34.2. C_18_H_12_N_4_ (284.31): Calcd C 76.04, H 4.25; Found C 76.29, H 4.22.

#### *2,5*-*bis(4*-*fluorophényl)*-*1*-*methyl*-*1H*-*imidazole (****14****)*

78% yield. ^1^H NMR (400 MHz, CDCl_3_) δ 7.65–7.55 (m, 2H), 7.40–7.30 (m, 2H), 7.15–7.00 (m, 5H), 3.57 (s, 3H). ^13^C NMR (100 MHz, CDCl_3_) δ 163.1 (d, *J *= 249.0 Hz), 162.7 (d, *J *= 249.0 Hz), 148.4, 134.4, 130.7 (d, *J *= 8.3 Hz), 130.5 (d, *J *= 8.3 Hz), 127.4, 126.9 (d, *J *= 3.3 Hz), 126.2 (d, *J *= 3.3 Hz), 115.9 (d, *J *= 17.3 Hz), 115.7 (d, *J *= 17.3 Hz), 33.6. C_16_H_12_F_2_N_2_ (270.28): Calcd C 71.10, H 4.48; Found C 71.02, H 4.34.

#### *2,5*-*bis(2*-*fluorophenyl)*-*1*-*methyl*-*1H*-*imidazole (****15****)*

59% yield. ^1^H NMR (400 MHz, CDCl_3_) δ 7.56 (t, *J *= 8.0 Hz, 1H), 7.42–7.30 (m, 3H), 7.25–7.08 (m, 5H), 3.42 (s, 3H). ^13^C NMR (100 MHz, CDCl_3_) δ 160.0 (dd, *J *= 249.2, 7.4 Hz), 144.7, 132.4 (d, *J *= 2.7 Hz), 131.9 (d, *J *= 2.7 Hz), 131.2 (d, *J *= 8.1 Hz), 130.4 (d, *J *= 8.1 Hz), 129.3, 124.6 (d, *J *= 3.4 Hz), 124.4 (d, *J *= 3.4 Hz), 119.1 (d, *J *= 14.9 Hz), 118.1 (d, *J *= 14.9 Hz), 116.1 (d, *J *= 13.2 Hz), 115.9 (d, *J *= 13.2 Hz), 32.4. C_16_H_12_F_2_N_2_ (270.28): Calcd C 71.10, H 4.48; Found C 71.27, H 4.55.

#### *1*-*méthyl*-*2,5*-*bis(3*-*(trifluoromethyl)phenyl)*-*1H*-*imidazole (****16****)*

59% yield. ^1^H NMR (400 MHz, CDCl_3_) δ 7.92 (s, 1H), 7.83 (d, *J *= 8.4 Hz, 1H), 7.65–7.50 (m, 6H), 7.21 (s, 1H), 3.63 (s, 3H).^13^C NMR (100 MHz, CDCl_3_) δ 148.5, 134.5, 131.9, 131.8, 131.5 (q, *J *= 20.4 Hz), 131.4, 131.1 (q, *J *= 20.4 Hz), 130.7, 129.4, 129.2, 128.6, 125.7, (q, *J *= 3.7 Hz), 125.6 (q, *J *= 3.7 Hz), 125.3 (q, *J *= 3.7 Hz), 124.8 (q, *J *= 3.7 Hz), 122.5, 33.8. C_18_H_12_F_6_N_2_ (370.29): Calcd C 58.38, H 3.27; Found C 58.47, H 3.45.

#### *2,5*-*bis(4*-*chlorophenyl)*-*1*-*methyl*-*1H*-*imidazole (17)*

70% yield. ^1^H NMR (400 MHz, CDCl_3_) δ 7.56 (d, *J *= 8.4 Hz, 2H), 7.39 (d, *J *= 8.4 Hz, 2H), 7.37 (d, *J *= 8.4 Hz, 2H), 7.30 (d, *J *= 8.4 Hz, 2H), 7.12 (s, 1H), 3.58 (s, 3H). ^13^C NMR (100 MHz, CDCl_3_) δ 148.2, 135.3, 134.6, 134.4, 130.1, 129.9, 129.2, 129.0, 128.5, 128.2, 127.1, 33.9. C_16_H_12_Cl_2_N_2_ (303.19): Calcd C 63.38, H 3.99; Found C 63.55, H 4.09.

#### *1*-*methyl*-*2,5*-*bis(4*-*nitrophenyl)*-*1H*-*imidazole (****18****)*

32% yield. ^1^H NMR (400 MHz, CDCl_3_) δ 8.39 (d, *J *= 8.4 Hz, 2H), 8.36 (d, *J *= 8.4 Hz, 2H), 8.07 (d, *J *= 8.4 Hz, 2H), 7.89 (d, *J *= 8.4 Hz, 2H), 7.55 (s, 1H), 3.83 (s, 3H). ^13^C NMR (100 MHz, CDCl_3_) δ 148.7, 147.6, 147.0, 136.8, 136.4, 135.1, 130.9, 130.0, 129.3, 124.6, 124.3, 35.0. C_16_H_12_N_4_O_4_ (324.29): Calcd C 59.26, H 3.73; Found C 59.04, H 3.49.

#### *1*-*methyl*-*2,5*-*bis(3*-*nitrophenyl)*-*1H*-*imidazole (****19****)*

62% yield. ^1^H NMR (400 MHz, CDCl_3_) δ 8.52 (s, 1H), 8.35–8.15 (m, 3H), 8.05 (d, *J *= 8.4 Hz, 1H), 7.74 (d, *J *= 8.4 Hz, 1H), 7.65 (d, *J *= 8.4 Hz, 1H), 7.62 (d, *J *= 8.4 Hz, 1H), 7.25 (s, 1H), 3.72 (s, 3H). ^13^C NMR (100 MHz, CDCl_3_) δ 148.6, 148.4, 147.9, 134.7, 134.3, 134.0, 131.9, 131.3, 130.1, 130.0, 129.4, 123.8, 123.4, 123.1, 123.0, 34.0. C_16_H_12_N_4_O_4_ (324.29): Calcd C 59.26, H 3.73; Found C 59.40, H 3.61.

#### *2,5*-*bis(4*-*methoxyphenyl)*-*1*-*methyl*-*1H*-*imidazole (****20****)*

78% yield. ^1^H NMR (400 MHz, CDCl_3_) δ 7.55 (d, *J *= 8.4 Hz, 2H), 7.30 (d, *J *= 8.4 Hz, 2H), 7.04 (s, 1H), 6.93 (d, *J *= 8.4 Hz, 2H), 6.92 (d, *J *= 8.4 Hz, 2H), 3.80 (s, 3H), 3.79 (s, 3H), 3.55 (s, 3H).

#### *4,4′*-*(1*-*methyl*-*1H*-*imidazole*-*2,5*-*diyle)dibenzaldehyde (****21****)*

62% yield. ^1^H NMR (400 MHz, CDCl_3_) δ 10.03 (s, 1H), 10.01 (s, 1H), 7.96 (d, *J *= 8.4 Hz, 2H), 7.93 (d, *J *= 8.4 Hz, 2H), 7.85 (d, *J *= 8.4 Hz, 2H), 7.59 (d, *J *= 8.4 Hz, 2H), 7.33 (s, 1H), 3.73 (s, 3H). ^13^C NMR (100 MHz, CDCl_3_) δ 191.6, 191.4, 149.3, 136.2, 135.9, 135.6, 131.5, 130.3, 130.0, 129.8, 129.2, 128.7, 127.5, 34.4. C_18_H_14_N_2_O_2_ (290.32): Calcd C 74.47, H 4.86; Found C 74.55, H 4.99.

#### *1,1′*-*((1*-*methyl*-*1H*-*imidazole*-*2,5*-*diyl)bis(4,1*-*phenylene))bis(propane*-*1*-*one) (****22****)*

65% yield. ^1^H NMR (400 MHz, CDCl_3_) δ 8.02 (d, *J *= 8.4 Hz, 2H), 7.98 (d, *J *= 8.4 Hz, 2H), 7.75 (d, *J *= 8.4 Hz, 2H), 7.50 (d, *J *= 8.4 Hz, 2H), 7.27 (s, 1H), 3.71 (s, 3H), 2.95 (q, *J *= 7.5 Hz, 4H), 1.15 (t, *J *= 7.5 Hz, 6H). ^13^C NMR (100 MHz, CDCl_3_) δ 200.1, 200.0, 149.0, 136.9, 136.2, 135.3, 134.1, 133.9, 128.9, 128.6, 128.4, 128.3, 34.4, 32.0, 31.9, 8.3, 8.2. C_22_H_22_N_2_O_2_ (346.42): Calcd C 76.28, H 6.40; Found C 76.08, H 6.21.

#### *1,1′*-*((1*-*methyl*-*1H*-*imidazole*-*2,5*-*diyl)bis(4,1*-*phenylene))bis(propane*-*1*-*one) (****23****)*

65% yield. ^1^H NMR (400 MHz, CDCl_3_) δ 8.02 (d, *J *= 8.4 Hz, 2H), 7.98 (d, *J *= 8.4 Hz, 2H), 7.75 (d, *J *= 8.4 Hz, 2H), 7.50 (d, *J *= 8.4 Hz, 2H), 7.27 (s, 1H), 3.71 (s, 3H), 2.95 (q, *J *= 7.5 Hz, 4H), 1.15 (t, *J *= 7.5 Hz, 6H). ^13^C NMR (100 MHz, CDCl_3_) δ 200.1, 200.0, 149.0, 136.9, 136.2, 135.3, 134.1, 133.9, 128.9, 128.6, 128.4, 128.3, 34.4, 32.0, 31.9, 8.3, 8.2. C_22_H_22_N_2_O_2_ (346.42): Calcd C 76.28, H 6.40.; Found C 76.08, H 6.21.

#### *1*-*benzyl*-*5*-*(4*-*chlorophenyl)*-*1H*-*imidazole (****24****)*

18% yield. ^1^H NMR (400 MHz, CDCl_3_) δ 7.51 (s, 1H), 7.28–7.15 (m, 5H), 7.12 (d, *J *= 8.4 Hz, 2H), 7.06 (s, 1H), 6.92 (d, *J *= 8.4 Hz, 2H), 5.06 (s, 2H). ^13^C NMR (100 MHz, CDCl_3_) δ 139.0, 136.5, 134.2, 132.3, 130.1, 129.0, 128.9, 128.6, 128.2, 128.1,126.5, 48.8.

#### *1*-*butyl*-*2,5*-*di*-*p*-*tolyl*-*1H*-*imidazole (****25****)*

25% yield. ^1^H NMR (400 MHz, CDCl_3_) δ 7.46 (d, *J *= 8.4 Hz, 2H), 7.25 (d, *J *= 8.4 Hz, 2H), 7.23–7.17 (m, 4H), 7.02 (s, 1H), 3.99 (t, *J *= 7.5 Hz, 2H), 2.34 (s, 6H), 1.26–1.15 (m, 2H), 0.90 0.83 (m, 2H), 0.54 (t, *J *= 7.5 Hz, 3H). ^13^C NMR (100 MHz, CDCl_3_) δ 149.1, 138.5, 137.8, 134.3, 129.4, 129.2, 128.8, 128.7, 128.0, 127.7, 44.7, 32.3, 21.4, 21.3, 19.3, 13.3. C_21_H_24_N_2_ (304.43): Calcd C 82.85, H 7.95; Found C 82.67, H 8.14.

#### *1*-*butyl*-*2,5*-*bis(4*-*chlorophenyl)*-*1H*-*imidazole (****26****)*

24% yield. ^1^H NMR (400 MHz, CDCl_3_) δ 7.48 (d, *J *= 8.4 Hz, 2H), 7.39 (d, *J *= 8.4 Hz, 2H),7.38 (d, *J *= 8.4 Hz, 2H), 7.31 (d, *J *= 8.4 Hz, 2H), 7.06 (s, 1H), 3.99 (t, *J *= 7.5 Hz, 2H), 1.26–1.15 (m, 2H), 0.90–0.83 (m, 2H), 0.57 (t, *J *= 7.5 Hz, 3H). ^13^C NMR (100 MHz, CDCl_3_) δ 148.3, 135.0, 134.2, 133.6, 130.2, 130.1, 129.9, 129.1, 128.9, 128.5, 44.9, 32.4, 19.3, 13.3. C_19_H_18_Cl_2_N_2_ (345.27): Calcd C 66.09, H 5.25; Found C 66.14, H 5.08.

#### *2,5*-*bis(3,5*-*bis(trifluoromethyl)phenyl)*-*1*-*methyl*-*1H*-*imidazole (****27****)*

48% yield. ^1^H NMR (400 MHz, CDCl_3_) δ 8.13 (s, 2H), 7.89 (s, 1H), 7.85 (s, 3H), 7.31 (s, 1H), 3.70 (s, 3H).^13^C NMR (100 MHz, CDCl_3_) δ 147.6, 133.8, 132.7 (q, *J *= 26.2 Hz), 132.1 (q, *J *= 26.2 Hz), 131.5, 129.6, 128.5, 128.4, 124.3, 122.8 (quint., *J *= 3.6 Hz), 122.1 (quint., *J *= 3.6 Hz), 121.6, 118.9, 33.9. C_20_H_10_F_12_N_2_ (506.29): Calcd C 47.45, H 1.99, N 5.53; Found C 47.40, H 2.09, N 5.36.

#### *1*-*benzyl*-*2,5*-*bis(3,5*-*bis(trifluoromethyl)phenyl)*-*1H*-*imidazole (****28****)*

32% yield. ^1^H NMR (400 MHz, CDCl_3_) δ 8.01 (s, 2H), 7.81 (s, 1H), 7.76 (s, 1H), 7.67 (s, 2H), 7.38 (s, 1H),7.28–7.20 (m, 3H), 6.81 (d, *J *= 8.4 Hz, 2H), 5.19 (s, 2H). ^13^C NMR (100 MHz, CDCl_3_) δ 147.7, 135.8, 133.5, 132.4 (q, *J *= 34.0 Hz), 132.3 (q, *J *= 34.0 Hz), 132.2, 131.6, 130.4, 129.4, 128.7 (m), 128.5, 125.4, 122.9 (q, *J *= 272.7 Hz), 122.8 (m), 122.7 (q, *J *= 272.7 Hz), 122.0 (m), 49.3. C_26_H_14_F_12_N_2_ (582.38): Calcd C 53.62, H 2.42, N 4.81; Found C 53.60, H 2.54, N 4.88

#### *2,5*-*bis(3,5*-*bis(trifluoromethyl)phenyl)*-*1*-*butyl*-*1H*-*imidazole (****29****)*

35% yield. ^1^H NMR (400 MHz, CDCl_3_) δ 8.05 (s, 2H), 7.91 (s, 1H), 7.88 (s, 1H), 7.84 (s, 2H), 7.26 (s, 1H), 4.04 (t, *J *= 7.5 Hz, 2H), 1.40–1.25 (m, 2H), 1.05–0.90 (m, 2H), 0.60 (t, *J *= 7.5 Hz, 3H). ^13^C NMR (100 MHz, CDCl_3_) δ 147.3, 133.0–131.5 (m), 130.4, 128.8, 128.6, 127.1, 124.4, 122.8 (q, J = *J *= 3.7 Hz), 122.1 (q, *J *= 3.7 Hz), 121.6, 118.9, 45.4, 32.6, 19.2, 13.0. C_23_H_16_F_12_N_2_ (548.37): Calcd C 50.38, H 2.94, N 5.11; Found C 50.19, H 2.78, N 4.83.

#### *4*-*(2,5*-*bis(3,5*-*bis(trifluoromethyl)phenyl)*-*1H*-*imidazol*-*1*-*yl)benzaldehyde (****30****)*

The product was obtained as trace observed by GC/MS analysis of the crude mixture.

#### *2,5*-*bis(3,5*-*bis(trifluoromethyl)phenyl)*-*1*-*(4*-*methoxyphenyl)*-*1H*-*imidazole (****31****)*

Low yield. ^1^H NMR (400 MHz, CDCl_3_) δ 7.79 (s, 2H), 7.70 (s, 1H), 7.66 (s, 1H), 7.50 (s, 1H), 7.47 (s, 2H), 7.03 (d, *J *= 8.4 Hz, 2H), 6.92 (d, *J *= 8.4 Hz, 2H), 3.78 (s, 3H).

#### *2,4,5*-*tris(3,5*-*bis(trifluoromethyl)phenyl)*-*1*-*methyl*-*1H*-*imidazole (****32****)*

30% yield. ^1^H NMR (400 MHz, CDCl_3_) δ 8.18 (s, 2H), 8.00 (s, 1H), 7.96 (s, 1H), 7.84 (s, 4H), 7.66 (s, 1H), 3.59 (s, 3H). ^13^C NMR (100 MHz, CDCl_3_) δ 145.7, 136.4, 134.0, 132.3 (q, *J *= 34.8 Hz), 131.5 (q, *J *= 34.8 Hz), 130.9 (q, *J *= 34.8 Hz), 130.7, 130.6, 129.6 (m), 128.6, 128.0 (m), 122.4 (m), 122.2 (m), 120.0 (m), 121.7 (q, *J *= 270.0 Hz), 121.6 (q, *J *= 270.0 Hz), 121.5 (q, *J *= 270.0 Hz), 32.6. C_28_H_12_F_18_N_2_ (718.38): Calcd C 46.81, H 1.68; Found C 46.99, H 1.88.

#### *2,4,5*-*tris(3,5*-*bis(trifluoromethyl)phenyl)*-*1*-*butyl*-*1H*-*imidazole (****33****)*

33% yield. ^1^H NMR (400 MHz, CDCl_3_) δ 8.11 (s, 2H), 8.02 (s, 1H), 7.97 (s, 1H), 7.85 (s, 2H), 7.78 (s, 2H), 7.64 (s, 1H), 3.89 (t, *J *= 7.5 Hz, 2H), 1.40–1.25 (m, 2H), 1.05–0.90 (m, 2H), 0.60 (t, *J *= 7.5 Hz, 3H).

#### *1*-*benzyl*-*2,4,5*-*tris(3,5*-*bis(trifluoromethyl)phenyl)*-*1H*-*imidazole (****34****)*

34% yield. ^1^H NMR (400 MHz, CDCl_3_) δ 8.00 (s, 2H), 7.89 (s, 1H), 7.87 (s, 1H), 7.84 (s, 2H), 7.64 (s, 1H), 7.59 (s, 2H), 7.25–7.20 (m, 3H), 6.73 6.81 (d, *J *= 8.4 Hz, 2H), 5.08 (s, 2H). ^13^C NMR (100 MHz, CDCl_3_) δ 146.9, 137.7, 135.2, 134.9, 133.1 (q, *J *= 34.0 Hz), 132.5 (q, *J *= 34.0 Hz), 131.1, 131.7, 131.6, 130.8 (m), 129.4, 129.0 (m), 128.7, 126.5 (m), 125.6, 123.3 (m), 122.9 (q, *J *= 272.7 Hz), 122.7 (q, *J *= 272.7 Hz), 122.5 (q, *J *= 272.7 Hz), 120.9 (m), 49.4.

### Bacterial strains and determination of the antibacterial activity

The antibacterial activity has been determined using the disc diffusion assay as previously described [36]. The measurements of inhibition zones were carried out three times for each drug including the antibiotic streptomycin as a positive control. Five bacterial strains were used in this study: *Escherichia coli* (DH5α), *Citrobacter freundii, Salmonella braenderup*, *Staphylococcus aureus,* and *Listeria monocytogenes*. The last four strains were provided from the Pasteur Institute of Casablanca Morocco.

### Fungal strains and determination of the antifungal activity

The compounds were evaluated for their antifungal activity using liquid cell culture against *Saccharomyces cerevisiae* (BY4741) and two Candida species: *Candida albicans* (SC5314) and *Candida krusei* (ATCC6258). Growth rate of yeast cells in liquid culture was monitored by measuring the absorbance of the cells at 600 nm (OD_600_) using a V-1200 spectrophotometer (Shanghai Mapada Instruments CO., LTD). The antifungal activity of a compound was evaluated as follows: Cells were grown overnight in yeast peptone dextrose medium (YPD) at 30 °C in a shaking incubator. Cells were then diluted to an OD_600_ of ~ 0.08 and allowed to grow until the OD_600_ reached ~ 0.14, to ensure that the cells were in logarithmic phase. Compound was then added and the OD_600_ was measured after 24 h of cell growth. The relative growth of yeast cells in the presence of a compound was then obtained by calculating the ratio of the OD_600_ determined for the treated cells to the OD_600_ of the untreated cells. All experiments were repeated at least twice and means were calculated.

### Determination of the IC_50_

IC_50_ is the concentration at which growth is inhibited by 50% in the presence of the compound. IC_50_s were determined as previously described [37].

### Statistical analysis

Statistical analyses were performed using one-way ANOVA Test in SPSS software version 21.0. The results were statistically considered significant when *p value* < 0.05.

### In silico ADME and toxicity predictions

In silico screening for prediction of the ADME properties (absorption, distribution, metabolism, excretion) of the studied compounds was performed with Molinspiration, a web-based software, (http://www.molinspiration.com), while screening for toxicity risks (mutagenicity, tumorogenicity, irritation, reproduction) was carried out with DataWarroir software [26, 38, 39].

### Molecular docking studies

The chemical structures of the studied molecules were sketched using ACD/ChemSketch, then optimized by the DFT/B3LYP method with 6–31G(d,p) basis sets using Gaussian 09 software [40].

The crystal structure of lanosterol 14α-demethylase from *S. cerevisiae* co-crystallized with the azole antifungal ligand Fluconazole (PDB: 4wmz) was obtained from the Protein Data Bank (http://www.pdb.org) and used as a target in docking studies. The docking studies were carried out using SwissDock web server which is based on the docking software EADock DSS [5, 41]. The analysis and the visualization of the docking results were performed using the UCSF Chimera molecular viewer [42].

## Additional file

**Additional file 1.** Supporting document showing the ^1^H and ^13^C NMR spectra of each compound studied in this paper.

## Data Availability

All data generated or analyzed during this study are included in this published article.

## Abbreviations

IC_50_: the concentration at which growth is inhibited by 50%
SAR: structure–activity relationship analysis
ADME: absorption, distribution, metabolism excretion properties
Tox: toxicity risks
CYP51: fungal lanosterol 14α-demethylase
