# Supplementary material for: Identification of novel antifungal agents: antimicrobial evaluation, SAR, ADME–Tox and molecular docking studies of a series of imidazole derivatives
Source: BMC Chem. 2019 Aug 6;13(1):100. doi: 10.1186/s13065-019-0623-6 (PMC6685181; doi:10.1186/s13065-019-0623-6)
Supplement: Supplementary file 1 — Additional file 1. Supporting document showing the 1H and 13C NMR spectra of each compound studied in this paper. [file 13065_2019_623_MOESM1_ESM.pdf]

1H and 13C NMR spectra:

Compound 1

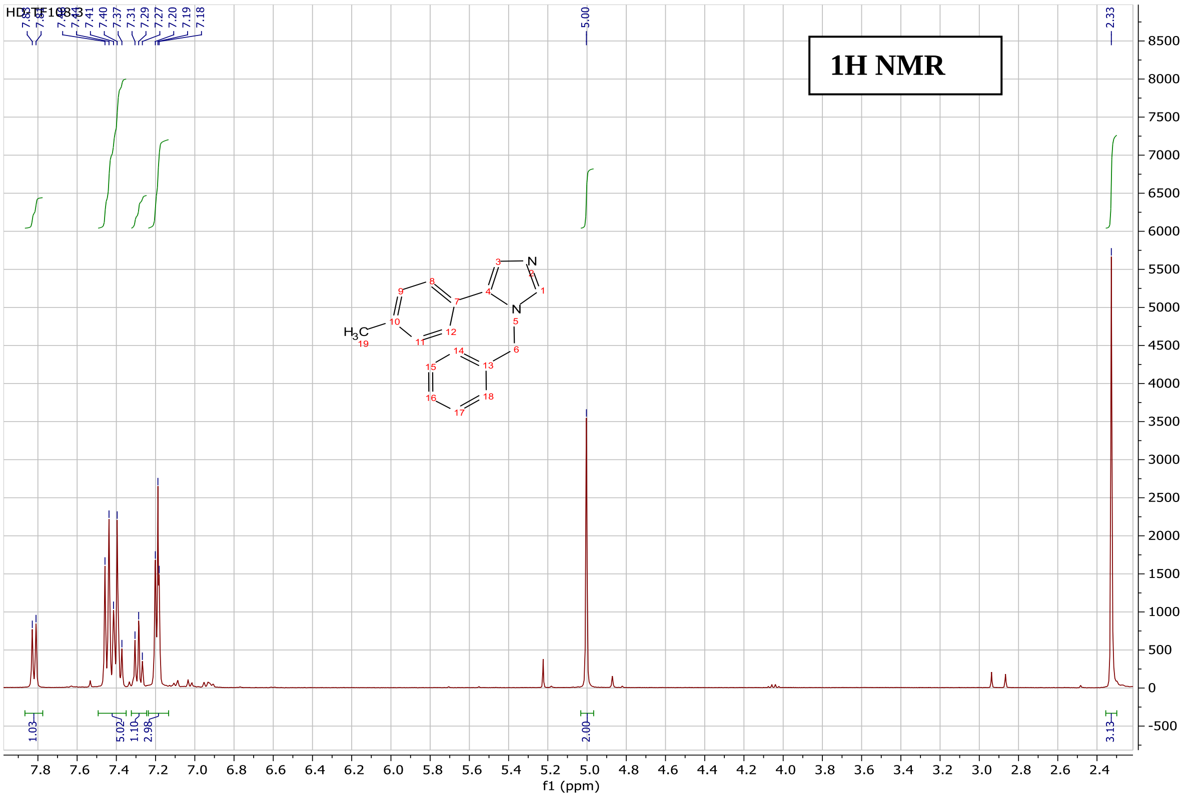

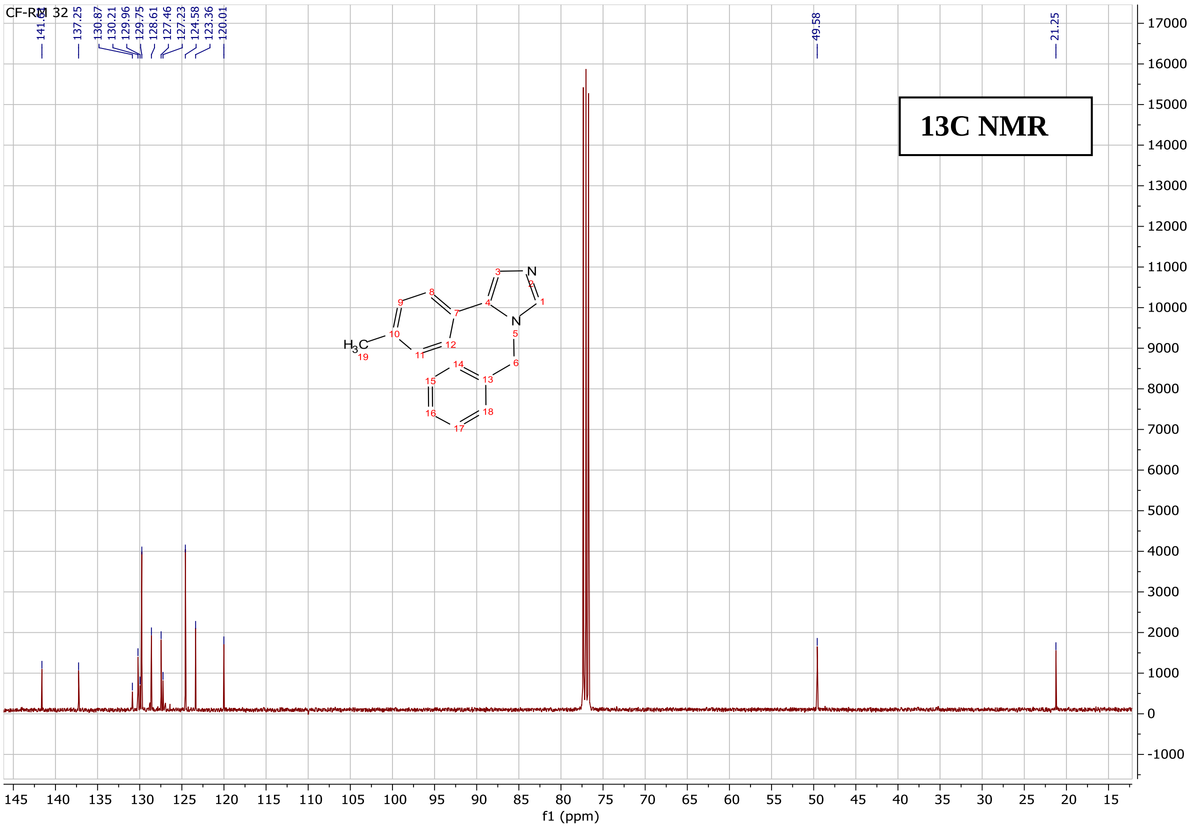

Compound 2

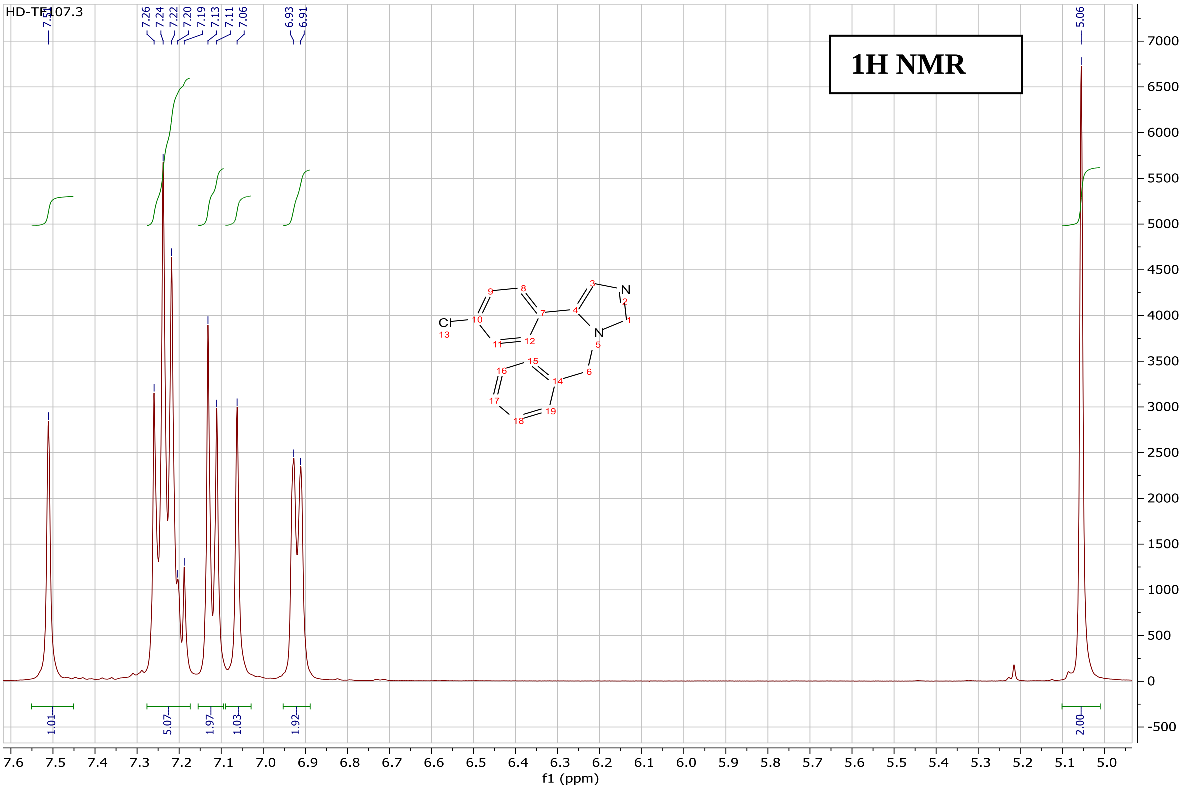

HD-TF107.3
1H NMR
Cl
N
f1 (ppm)

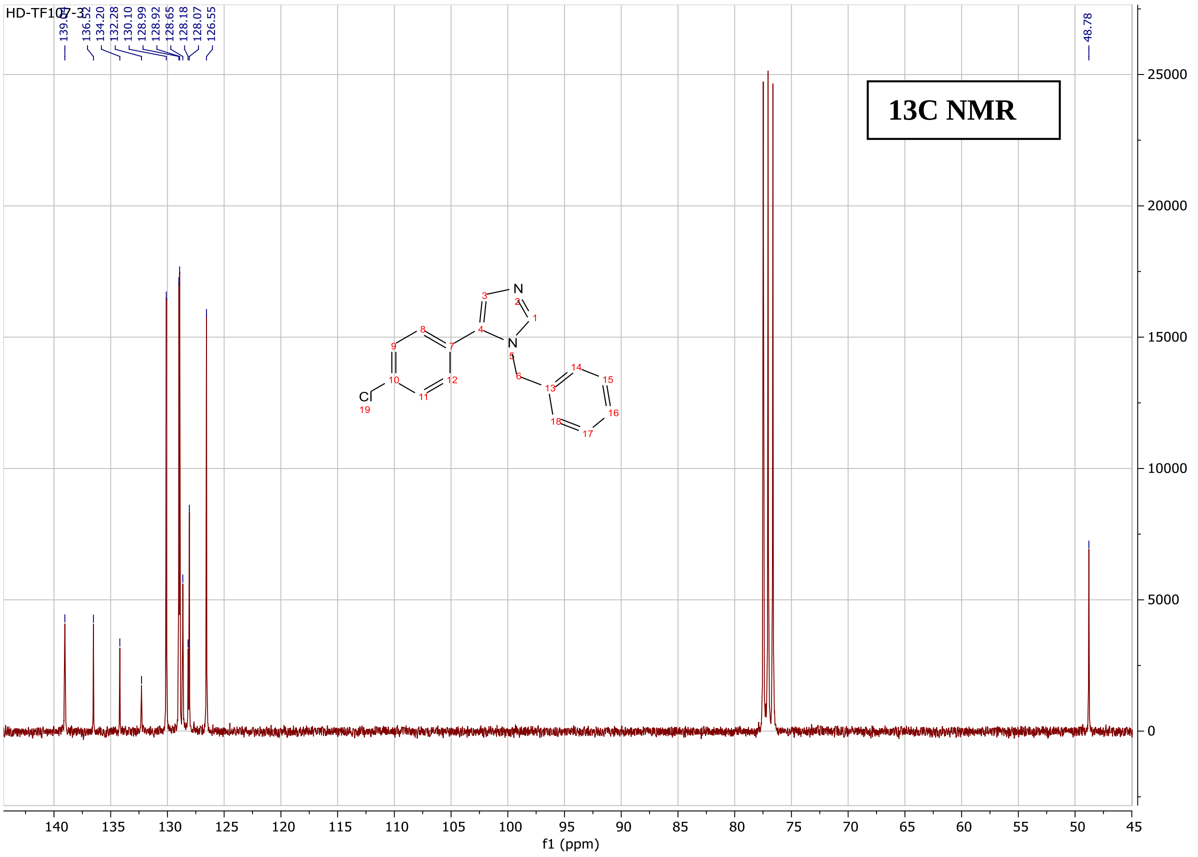

13C NMR
134.20
132.28
130.10
128.99
128.92
128.65
128.18
128.07
126.55
48.78
Cl
N
f1 (ppm)

Compound 3

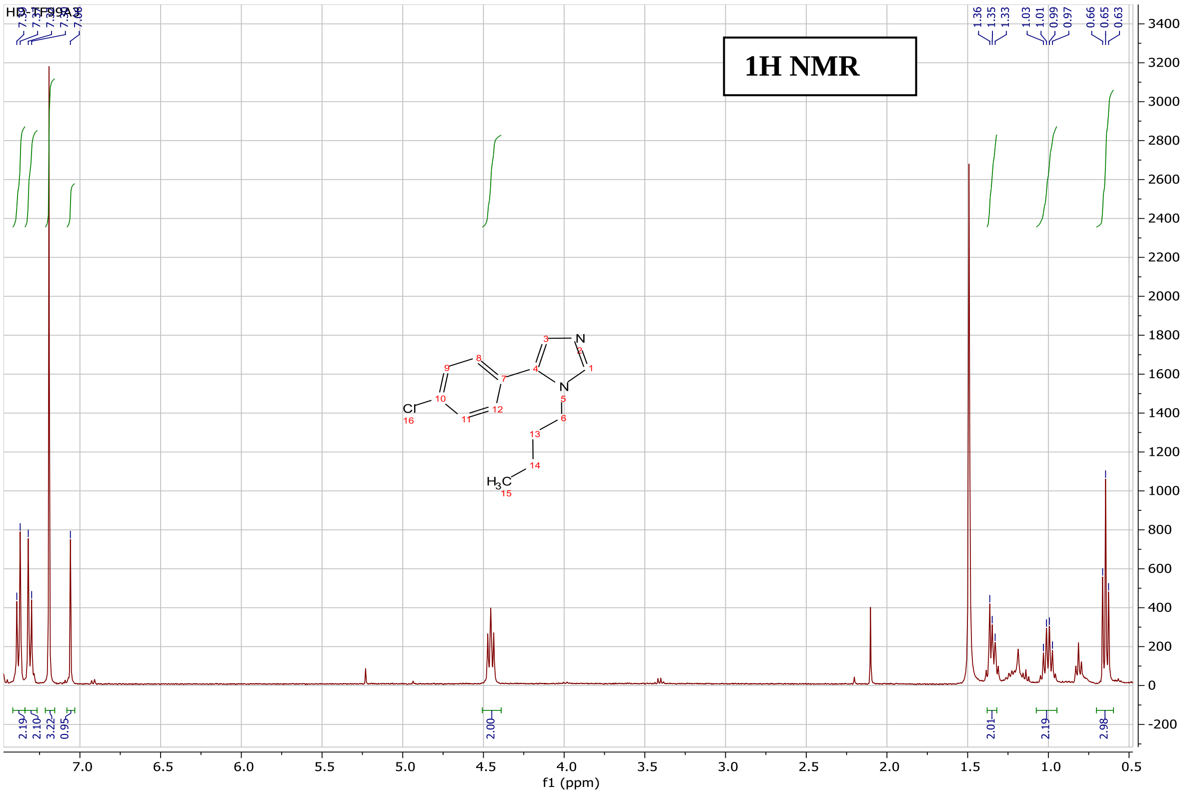
HD-TF99A3
1H NMR
1.36
1.35
1.33
1.03
1.01
0.99
0.97
0.66
0.65
0.63
2.19
2.10
3.22
0.95
2.00
2.01
2.19
2.98
Cl
H3C
N
f1 (ppm)

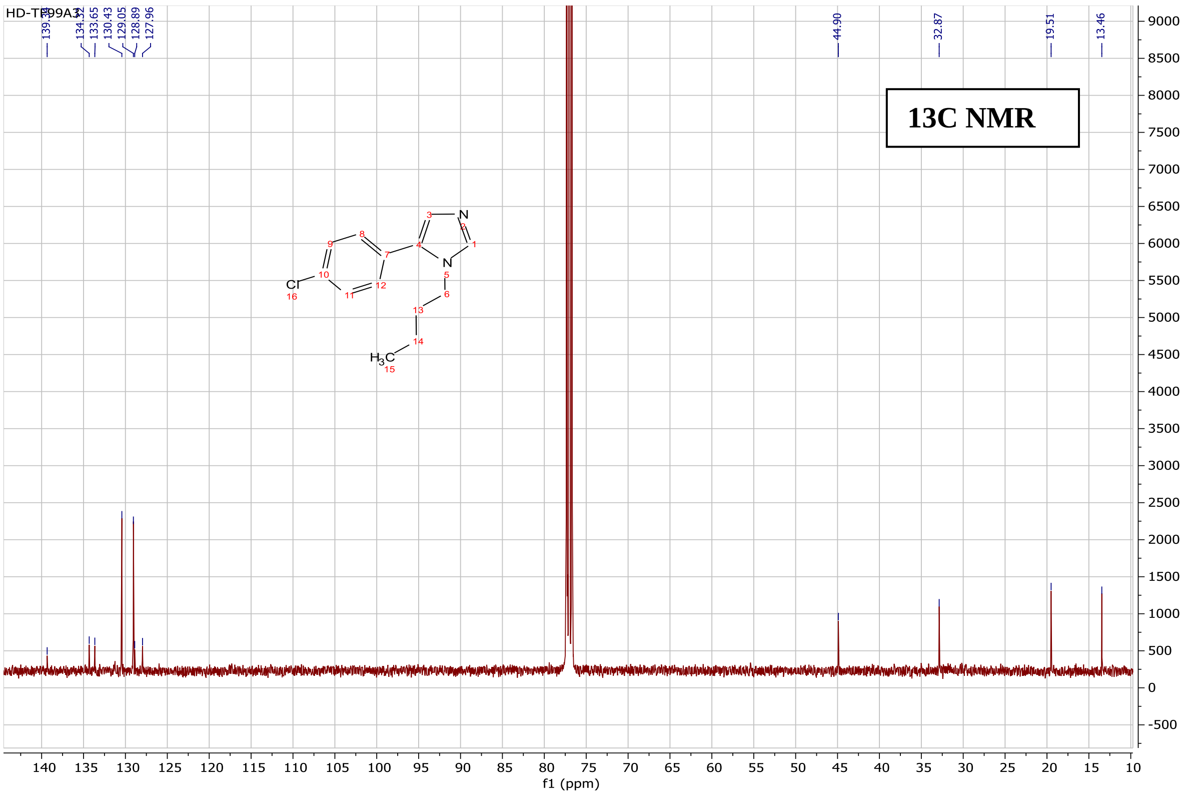
HD-TF99A3
13C NMR
133.65
130.43
129.05
128.89
127.96
44.90
32.87
19.51
13.46
Cl
H3C
N
f1 (ppm)

Compound 5

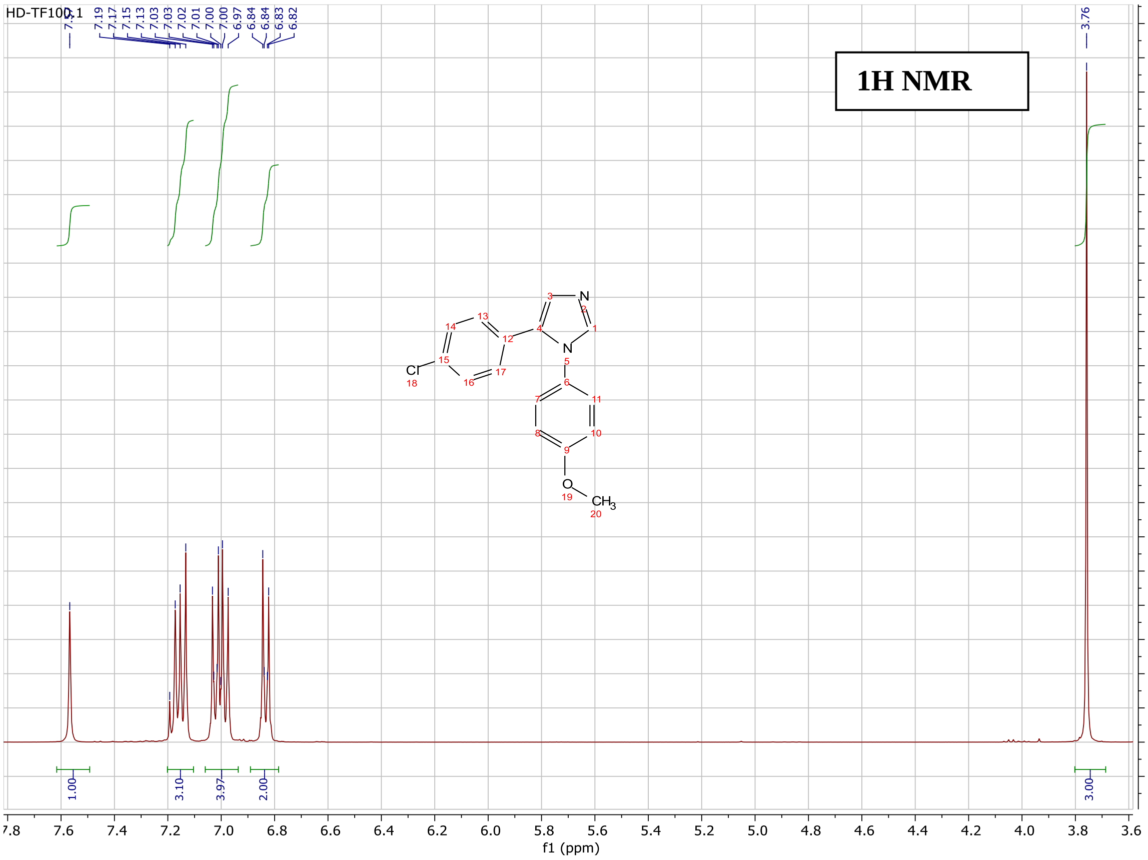

HD-TF100.1
1H NMR
7.19
7.17
7.15
7.13
7.03
7.03
7.02
7.01
7.00
7.00
6.97
6.84
6.84
6.83
6.82
3.76
1.00
3.10
3.97
2.00
3.00
Cl
N
O
CH3
f1 (ppm)

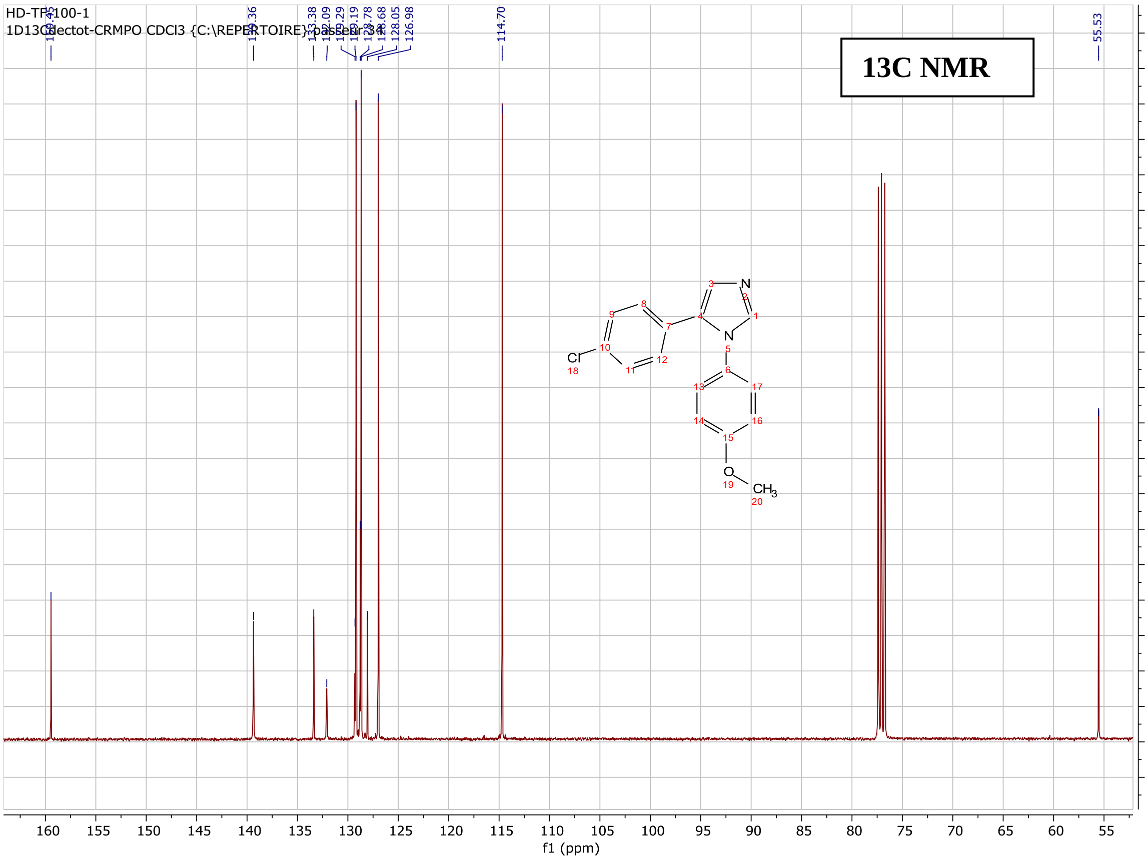

HD-TF100-1
13C NMR
133.38
132.09
129.29
129.19
128.78
128.68
128.05
126.98
114.70
55.53
Cl
N
O
CH3
f1 (ppm)

Compound 6

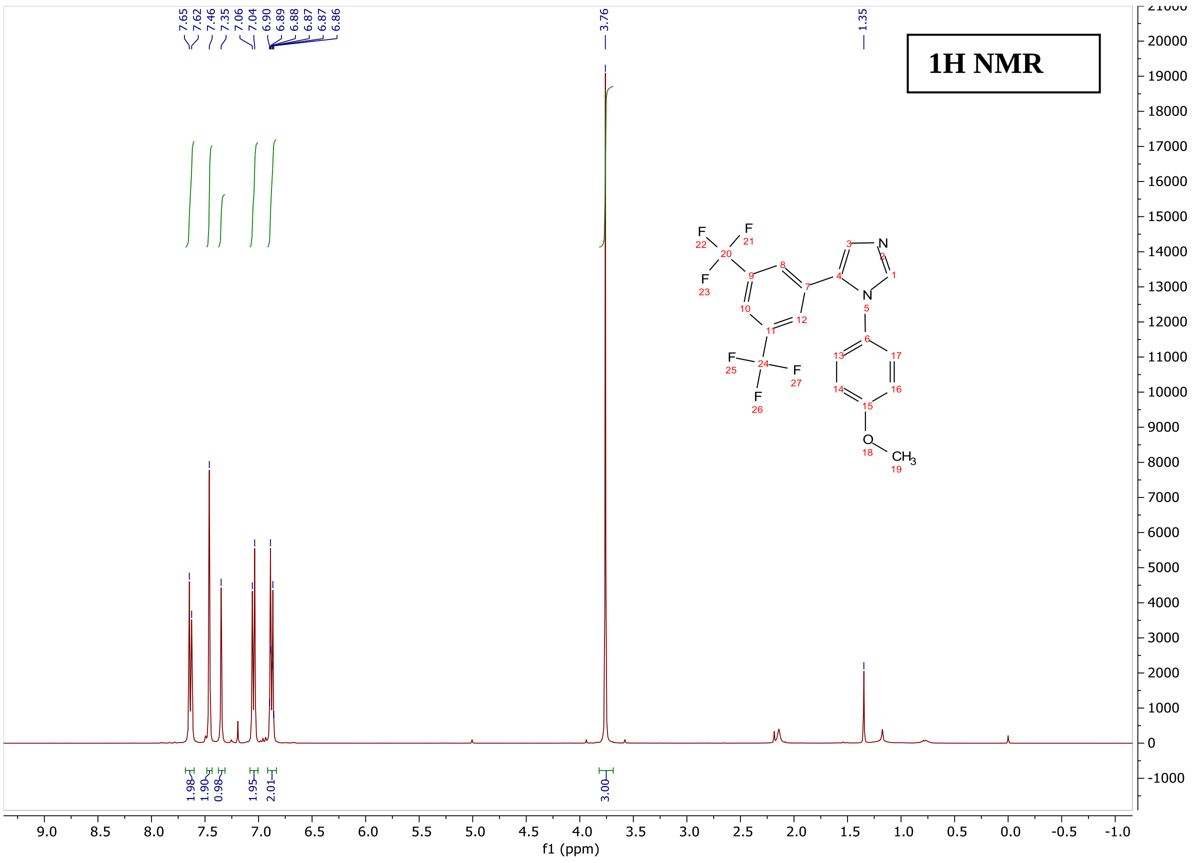

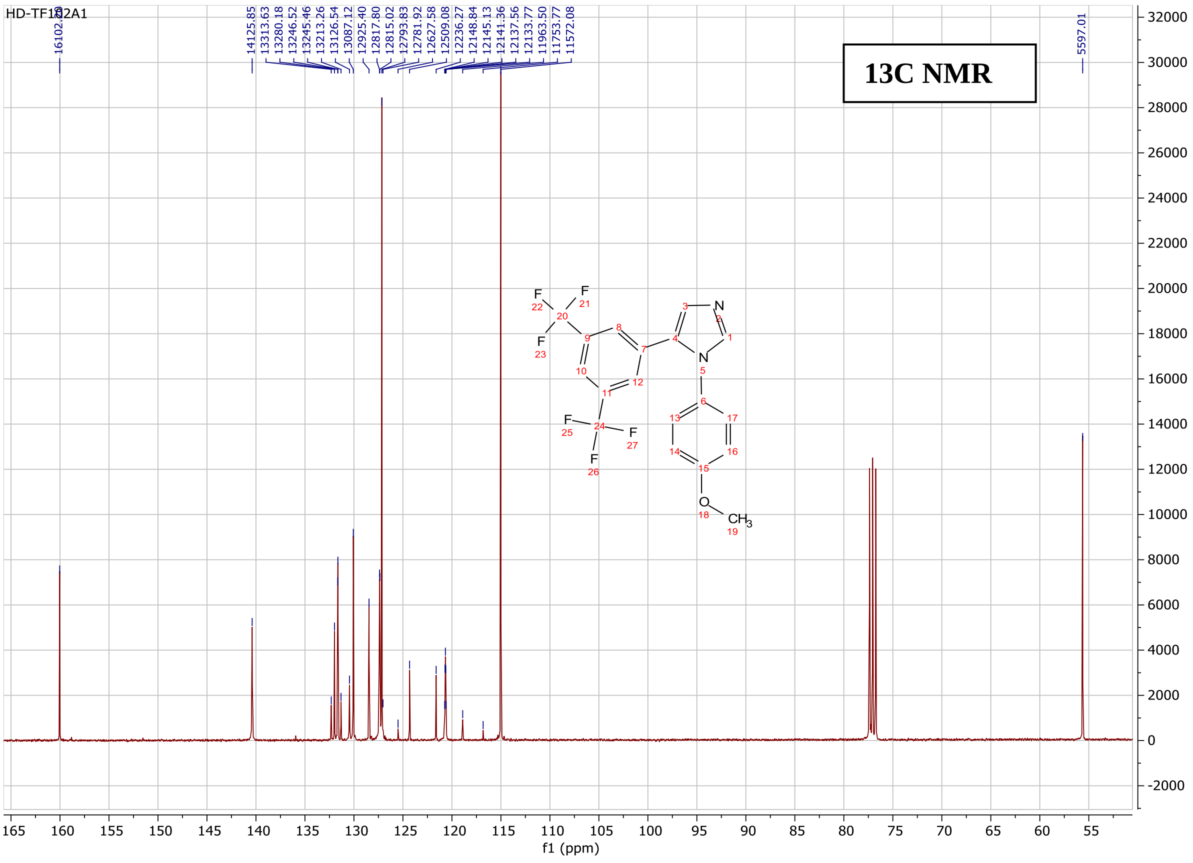

Compound 7

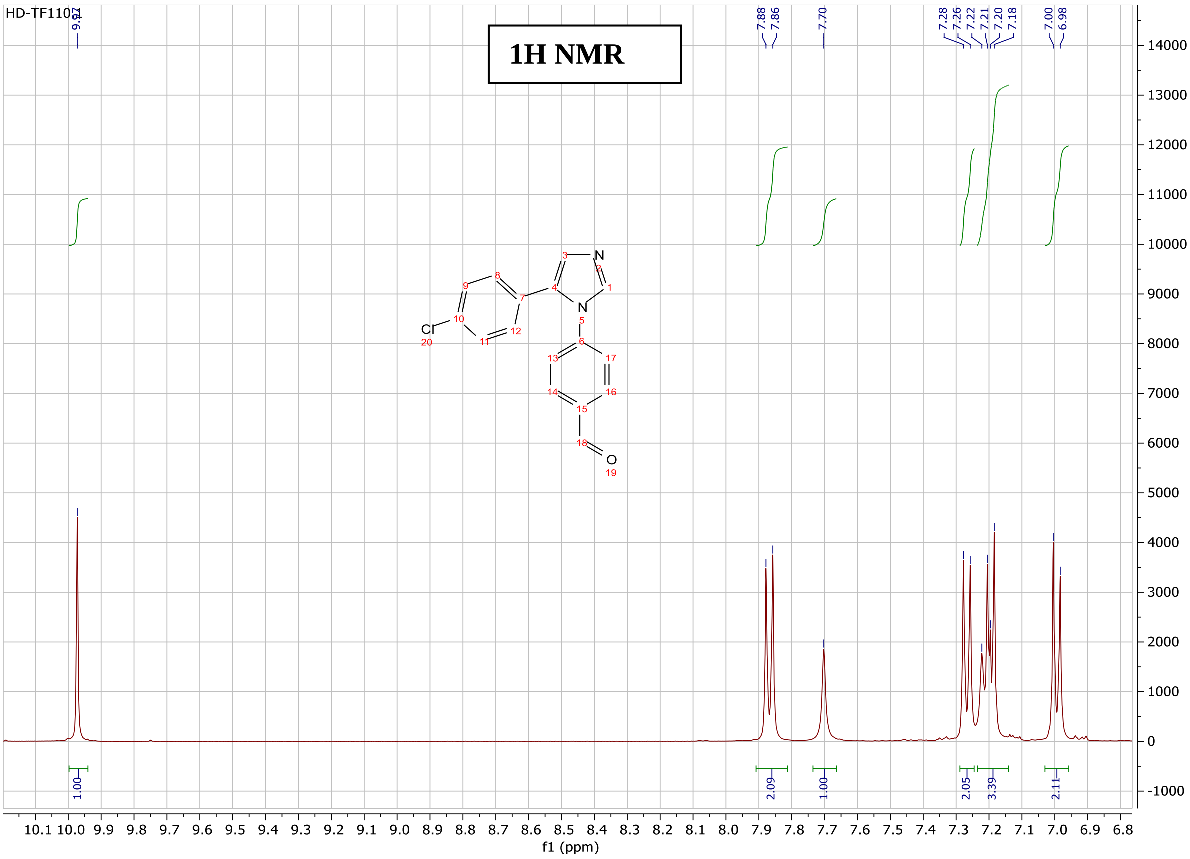

HD-TF110-1
1H NMR
9.97
7.88
7.86
7.70
7.28
7.26
7.22
7.21
7.20
7.18
7.00
6.98
1.00
2.09
1.00
2.05
3.39
2.11
Cl
N
O
f1 (ppm)

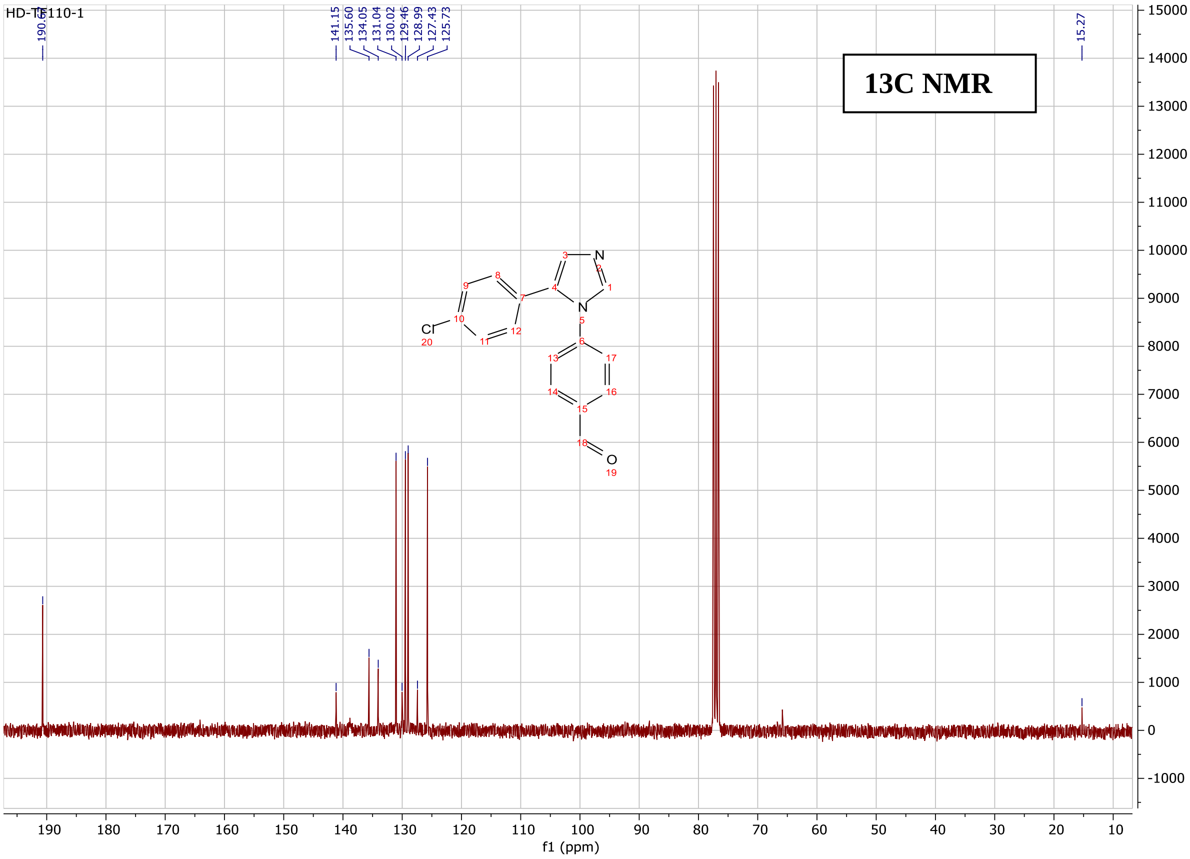

HD-TF110-1
13C NMR
190.61
141.15
135.60
134.05
131.04
130.02
129.46
128.99
127.43
125.73
15.27
Cl
N
O
f1 (ppm)

Compound 8

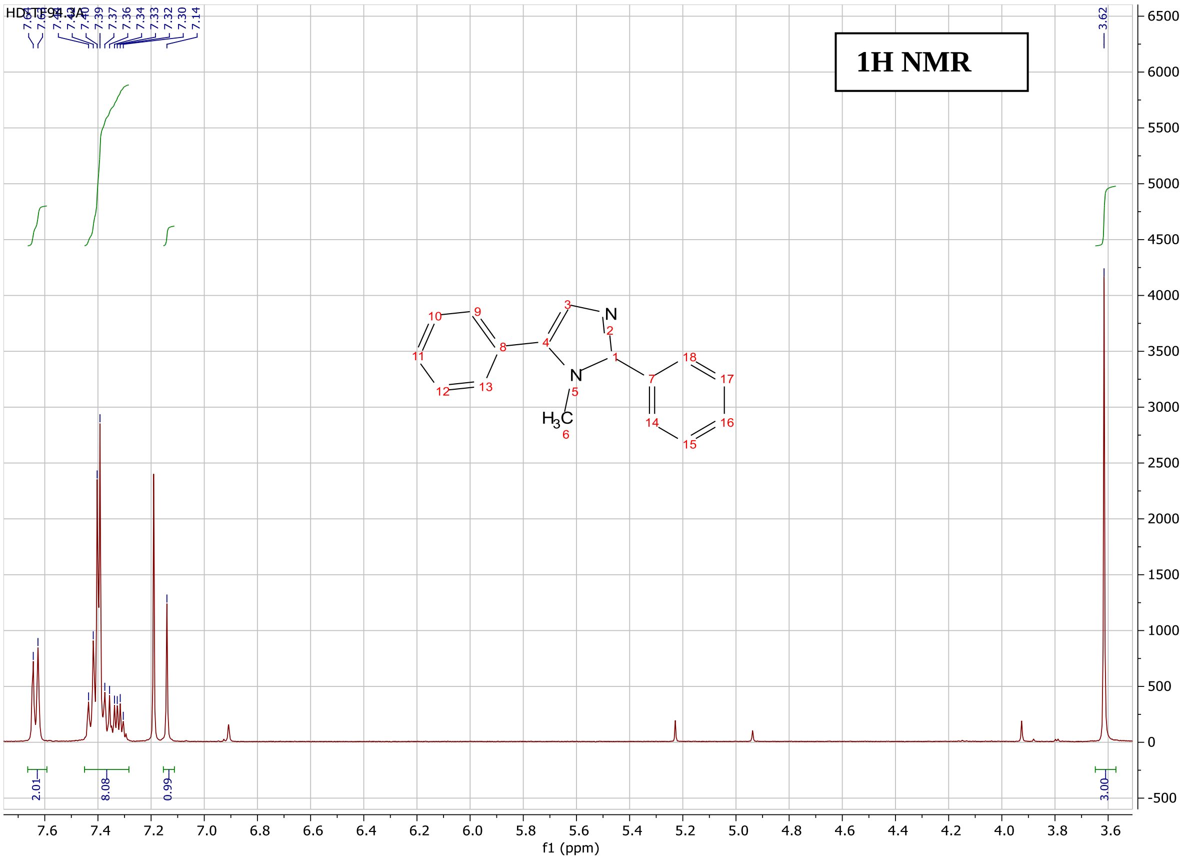

Compound 9

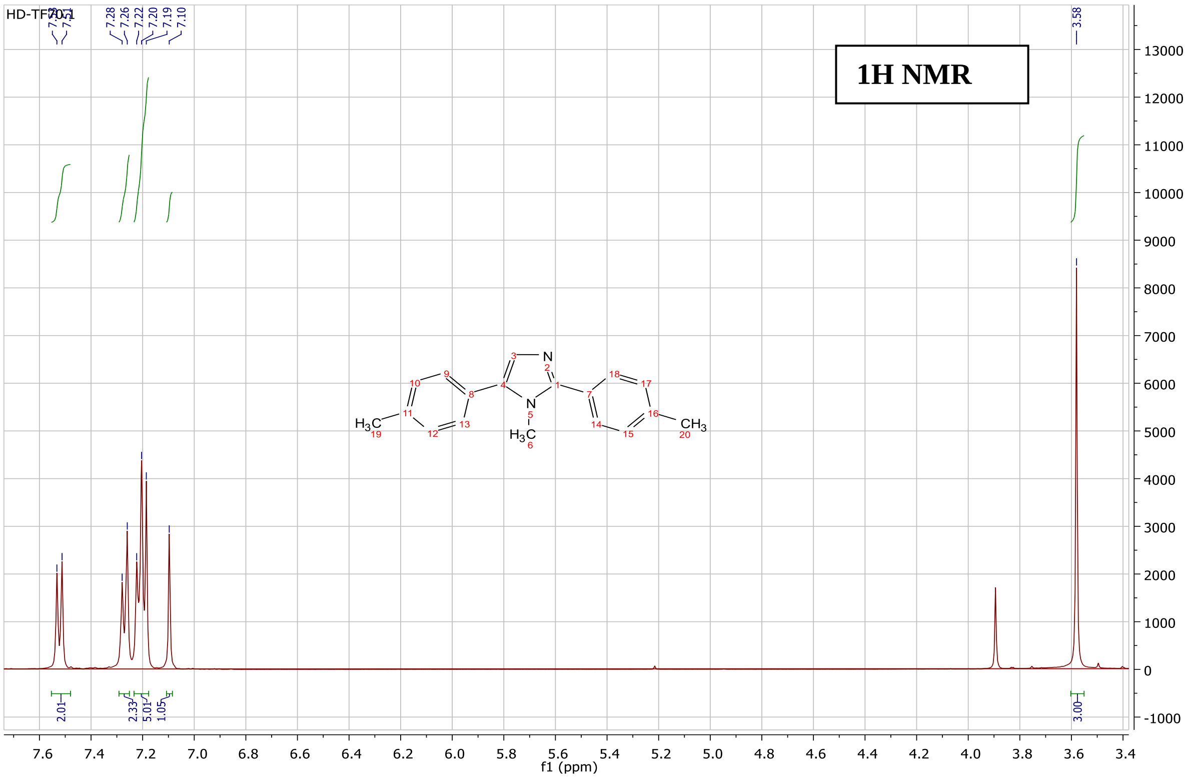
HD-TF70.1
7.28
7.26
7.22
7.20
7.19
7.10
3.58
1H NMR
2.01
2.33
5.01
1.05
3.00
f1 (ppm)

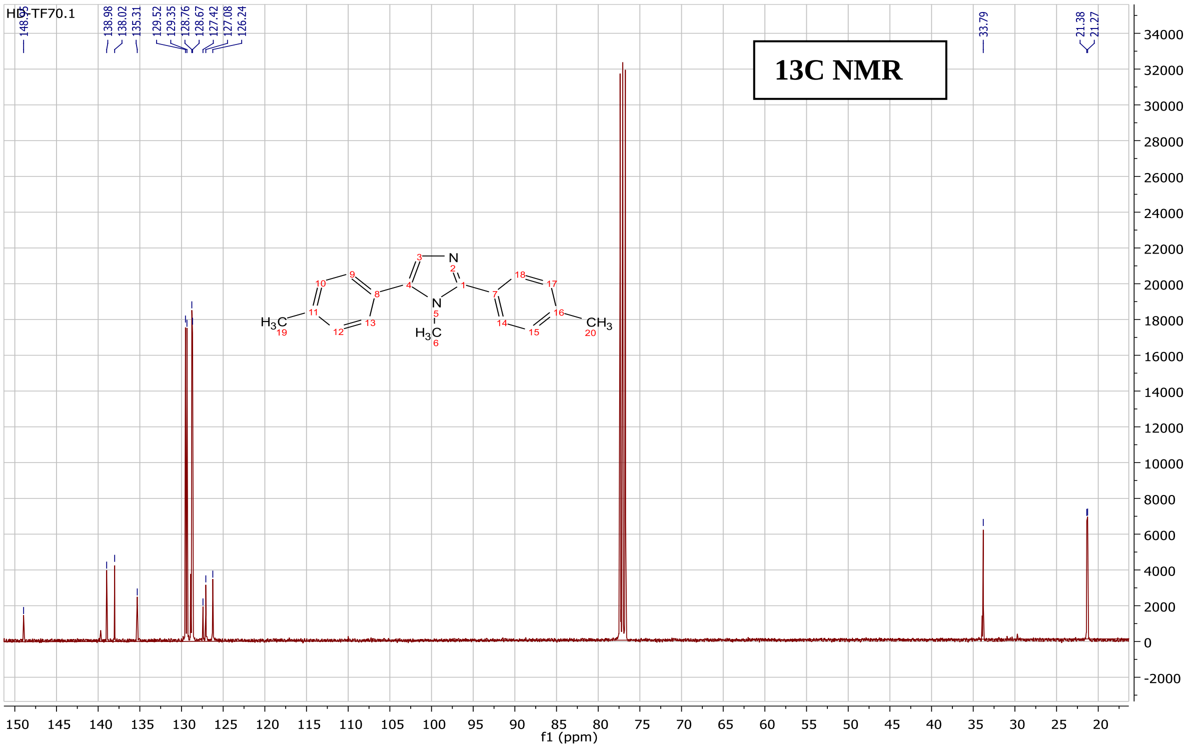
HD-TF70.1
138.98
138.02
135.31
129.52
129.35
128.76
128.67
127.42
127.08
126.24
33.79
21.38
21.27
13C NMR
f1 (ppm)

Compound 10

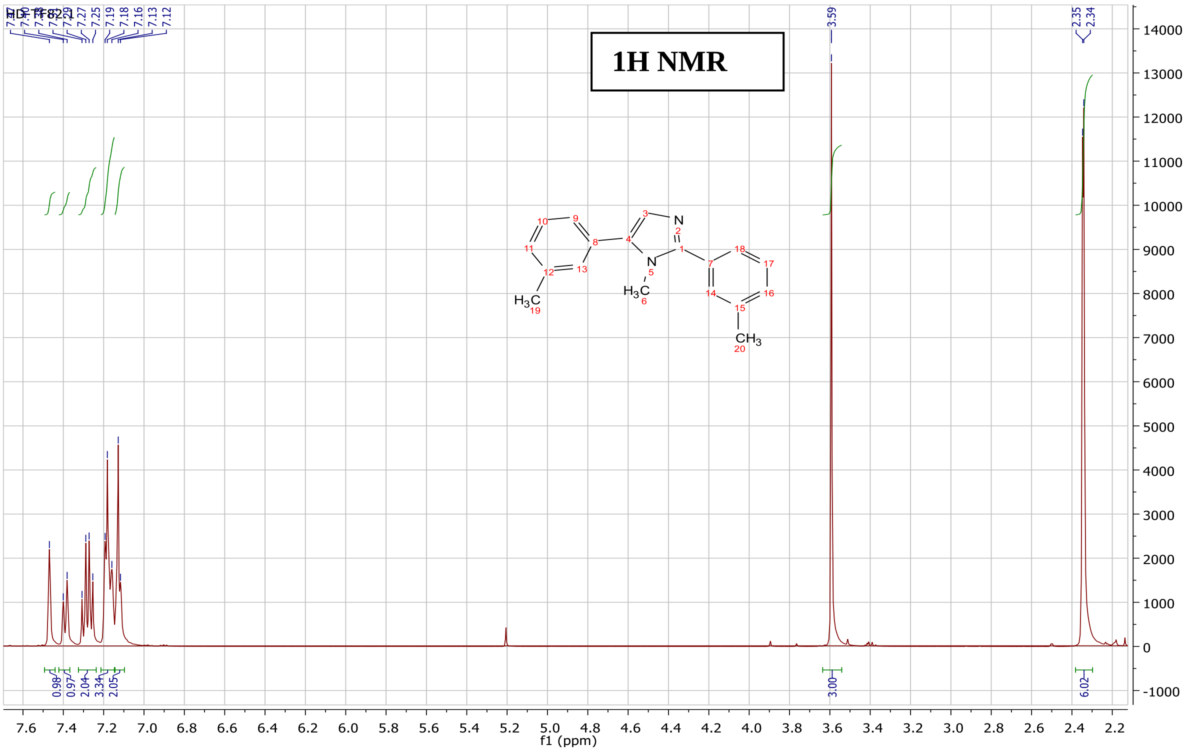
1H NMR
HD-TF82.1
3.59
2.35
2.34
0.98
0.97
2.04
3.34
2.05
3.00
6.02
f1 (ppm)

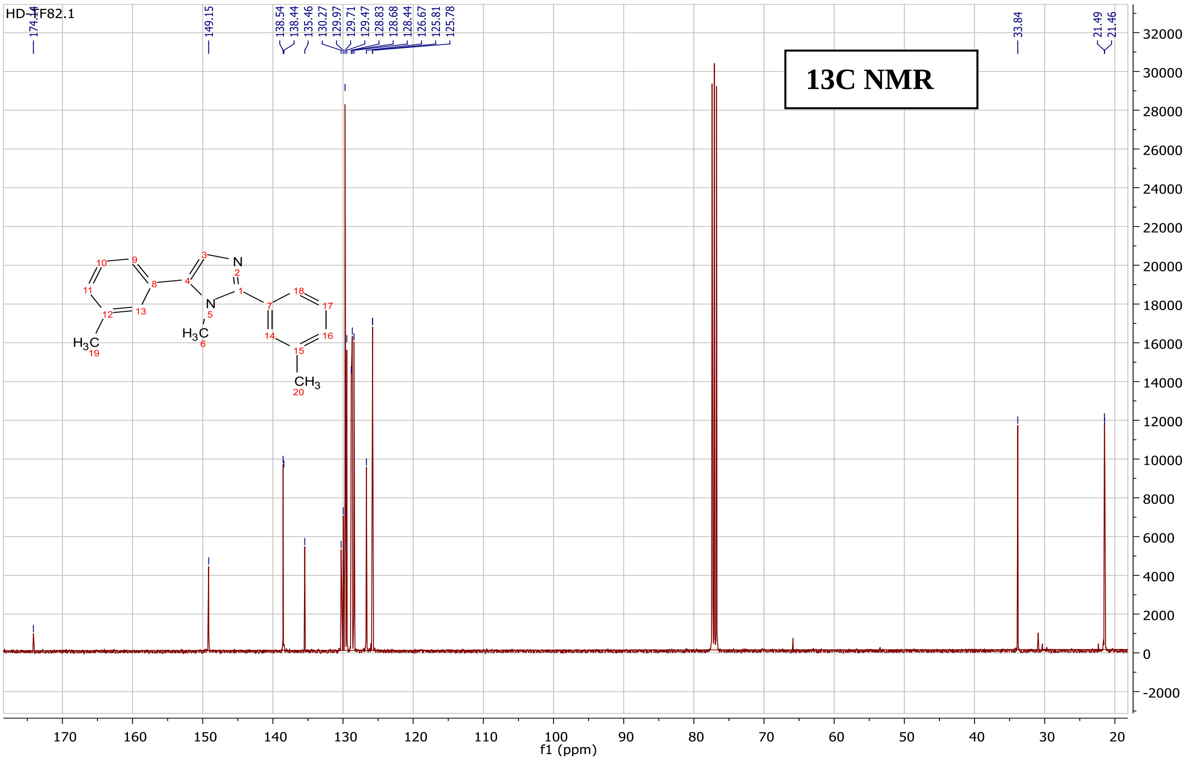
13C NMR
HD-TF82.1
174.44
149.15
138.54
138.44
135.46
130.27
129.97
129.71
129.47
128.83
128.68
128.44
126.67
125.81
125.78
33.84
21.49
21.46
f1 (ppm)

Compound 11

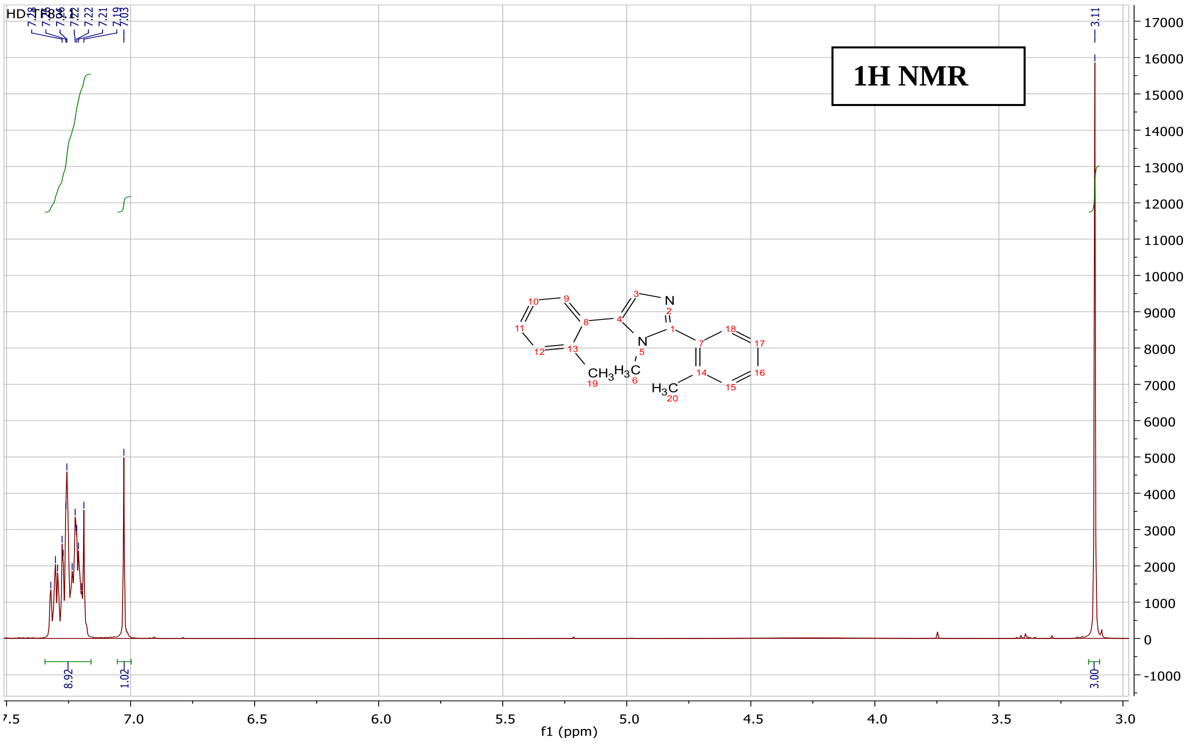
HD-TF83.1
7.03
3.11
1H NMR
8.92
1.02
3.00
f1 (ppm)

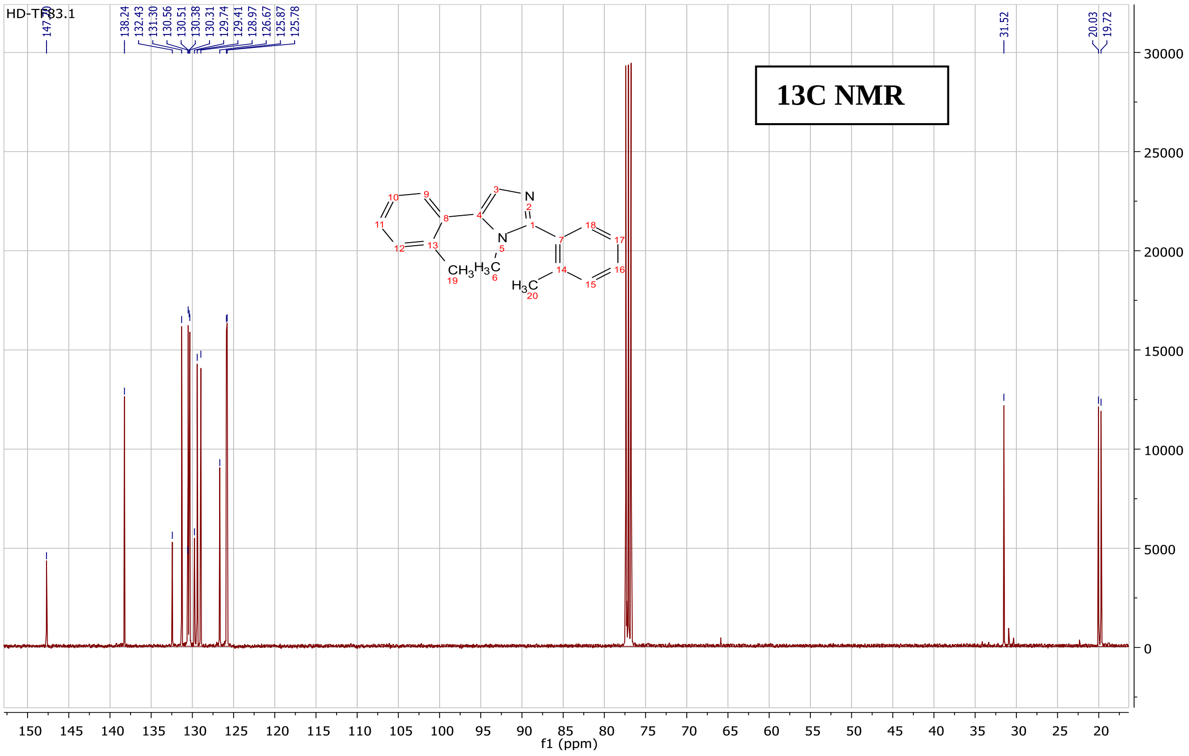
HD-TF83.1
138.24
132.43
131.30
130.56
130.51
130.38
130.31
129.74
129.41
128.97
126.67
125.87
125.78
31.52
20.03
19.72
13C NMR
f1 (ppm)

Compound 12

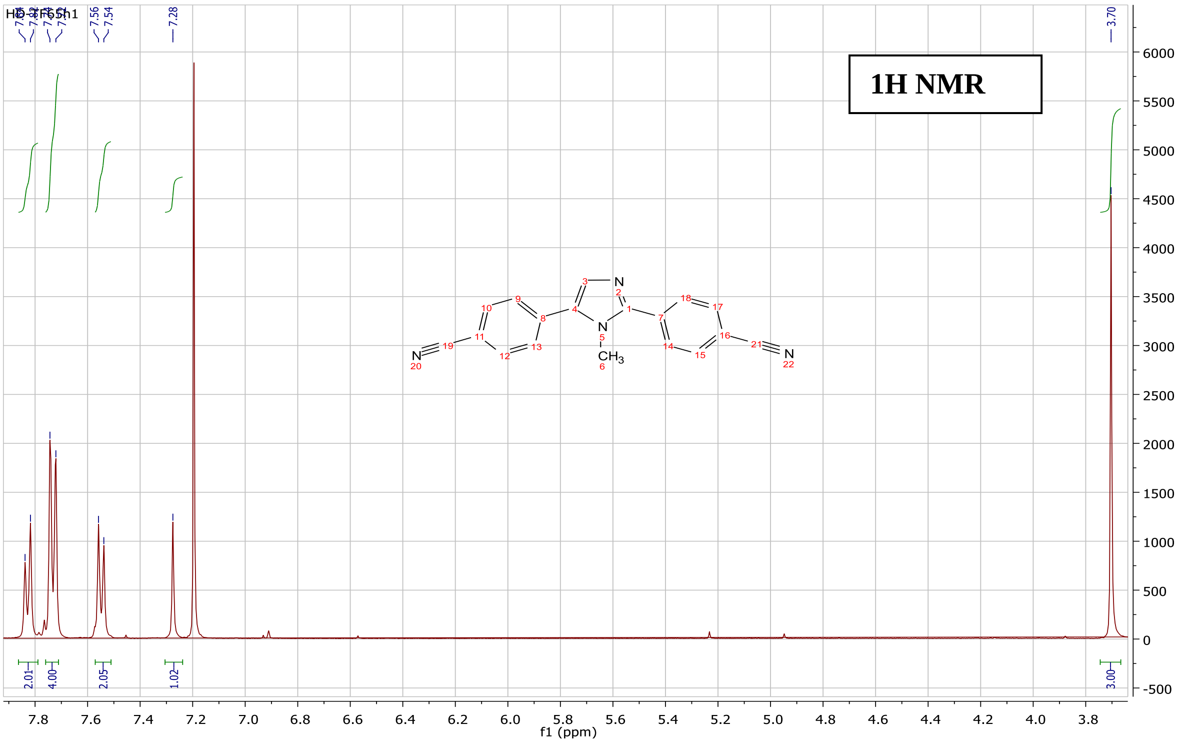

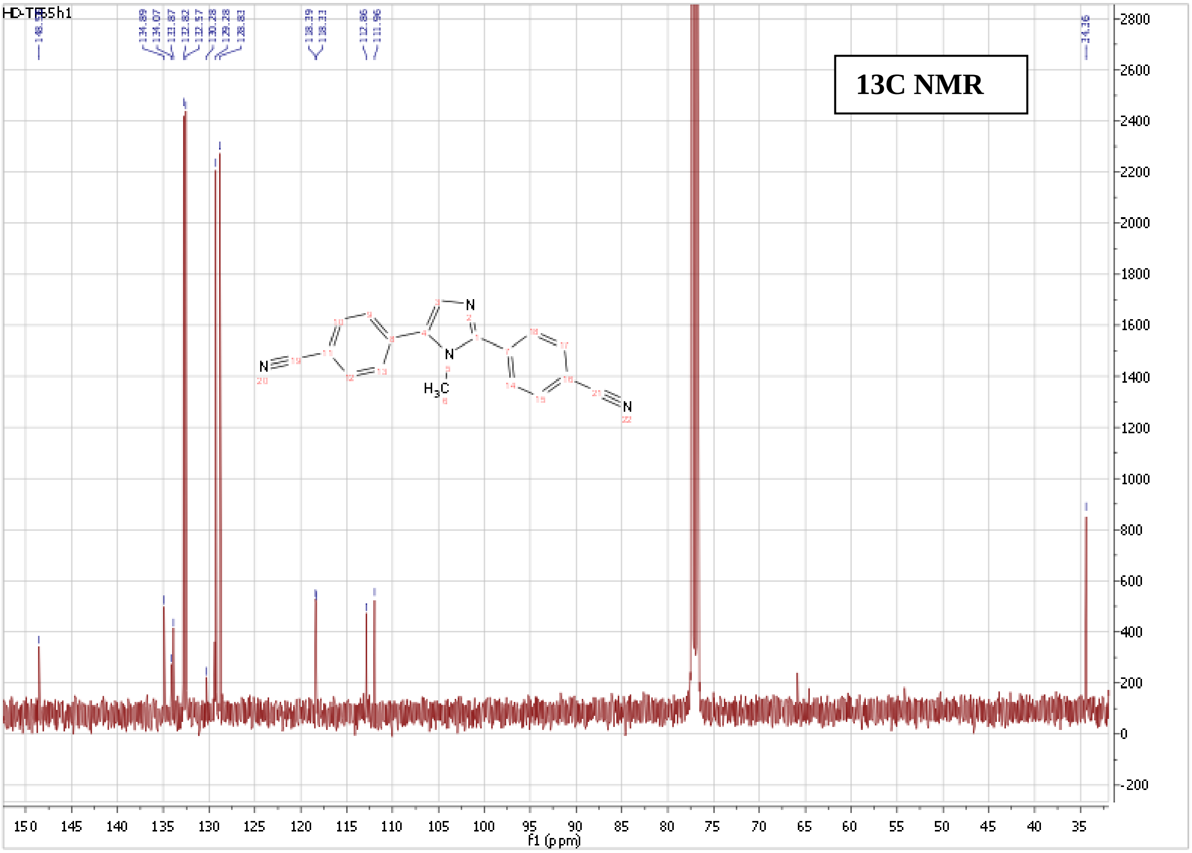

Compound 13

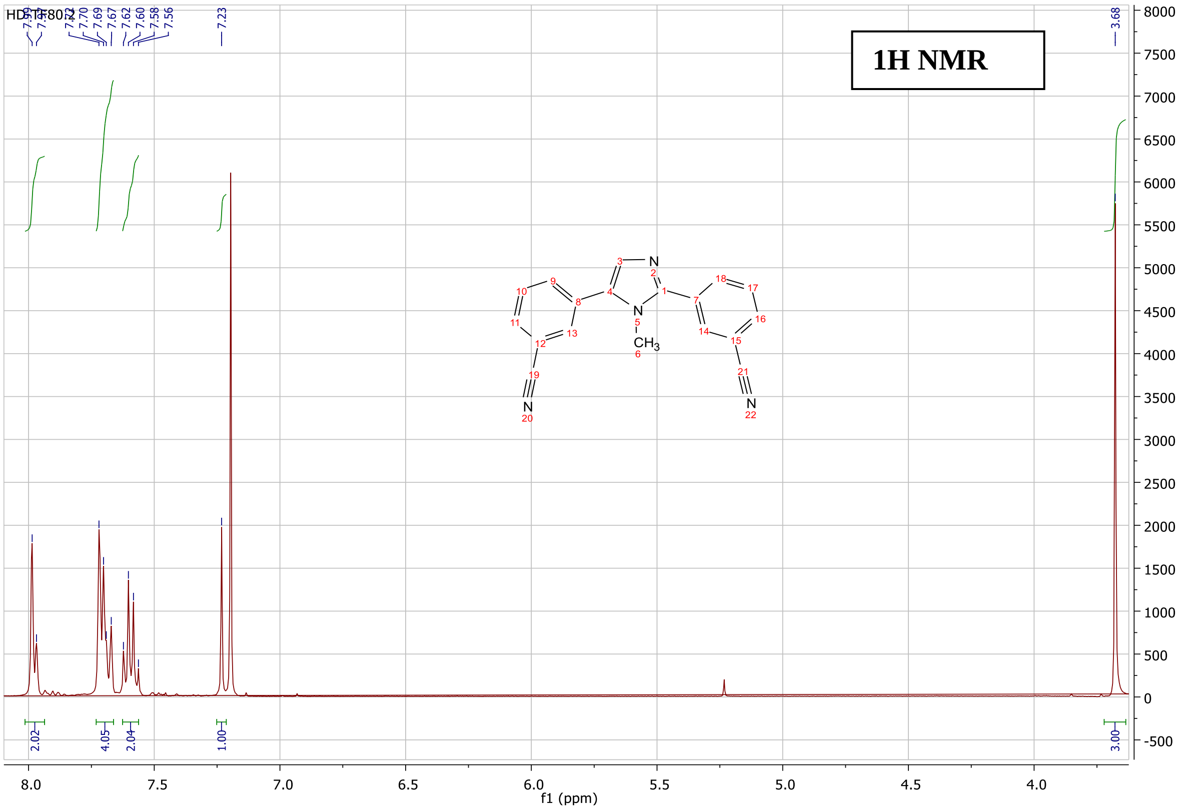

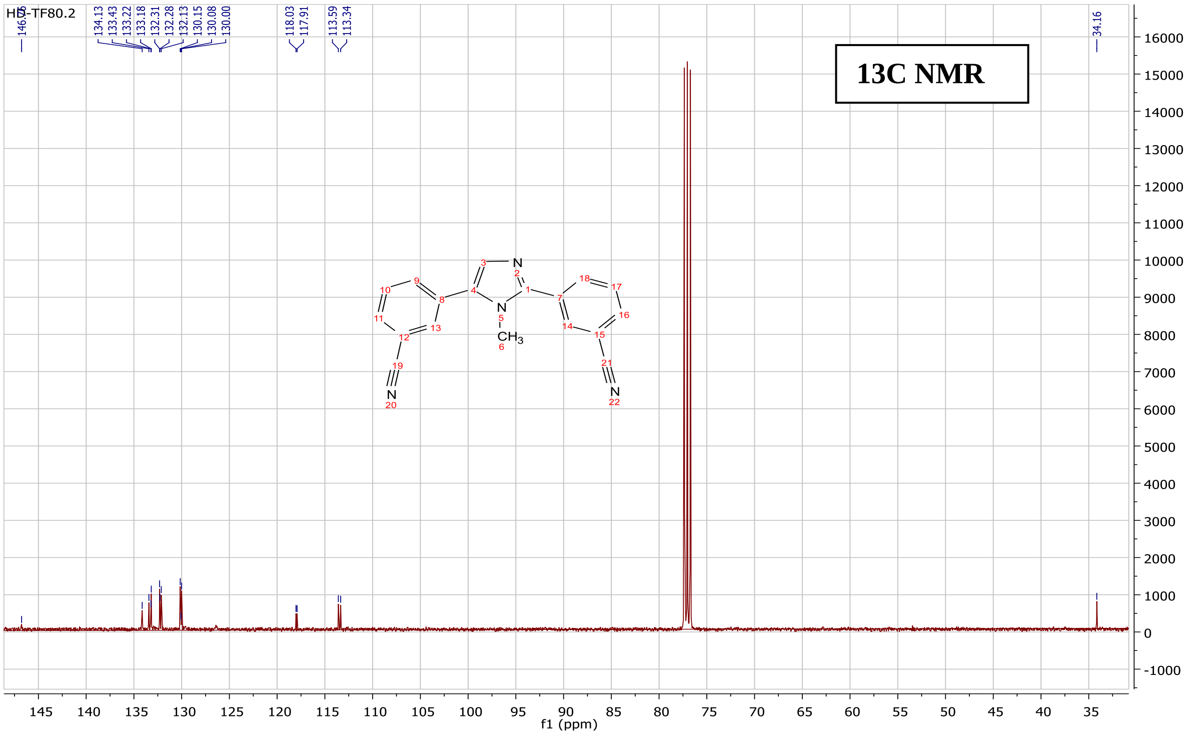

Compound 14

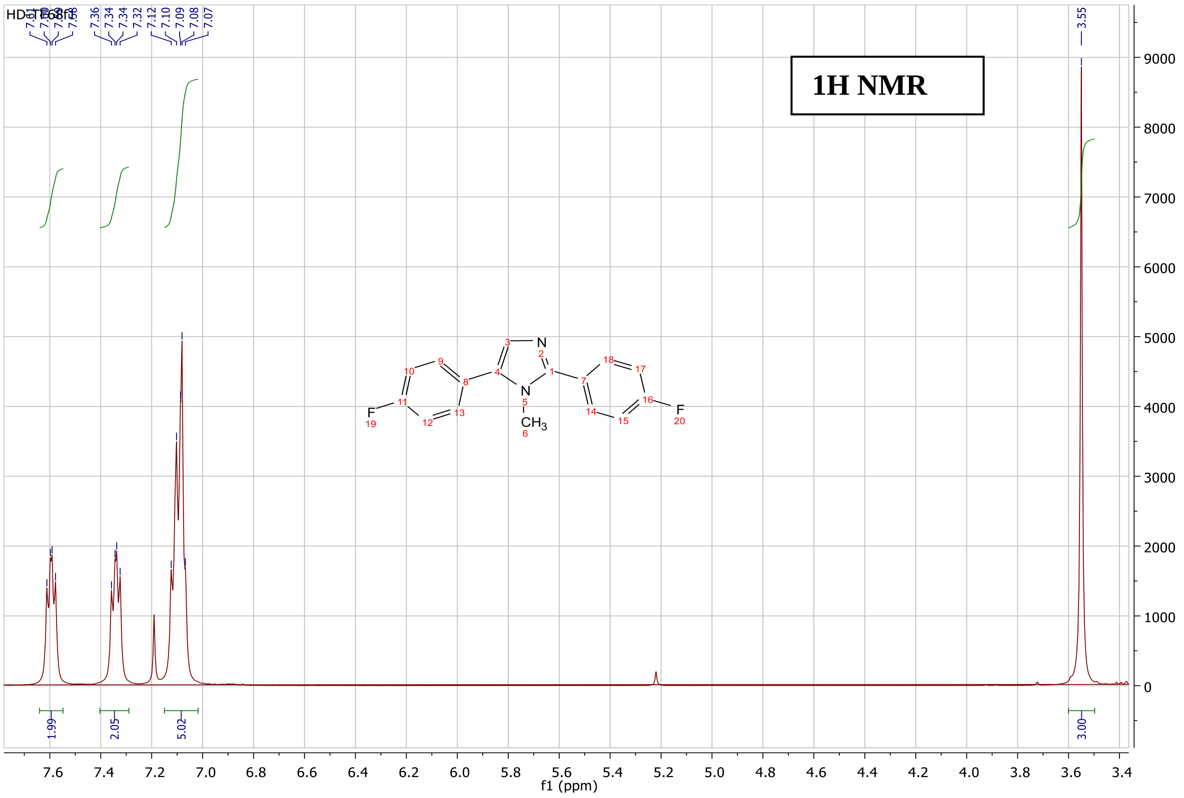

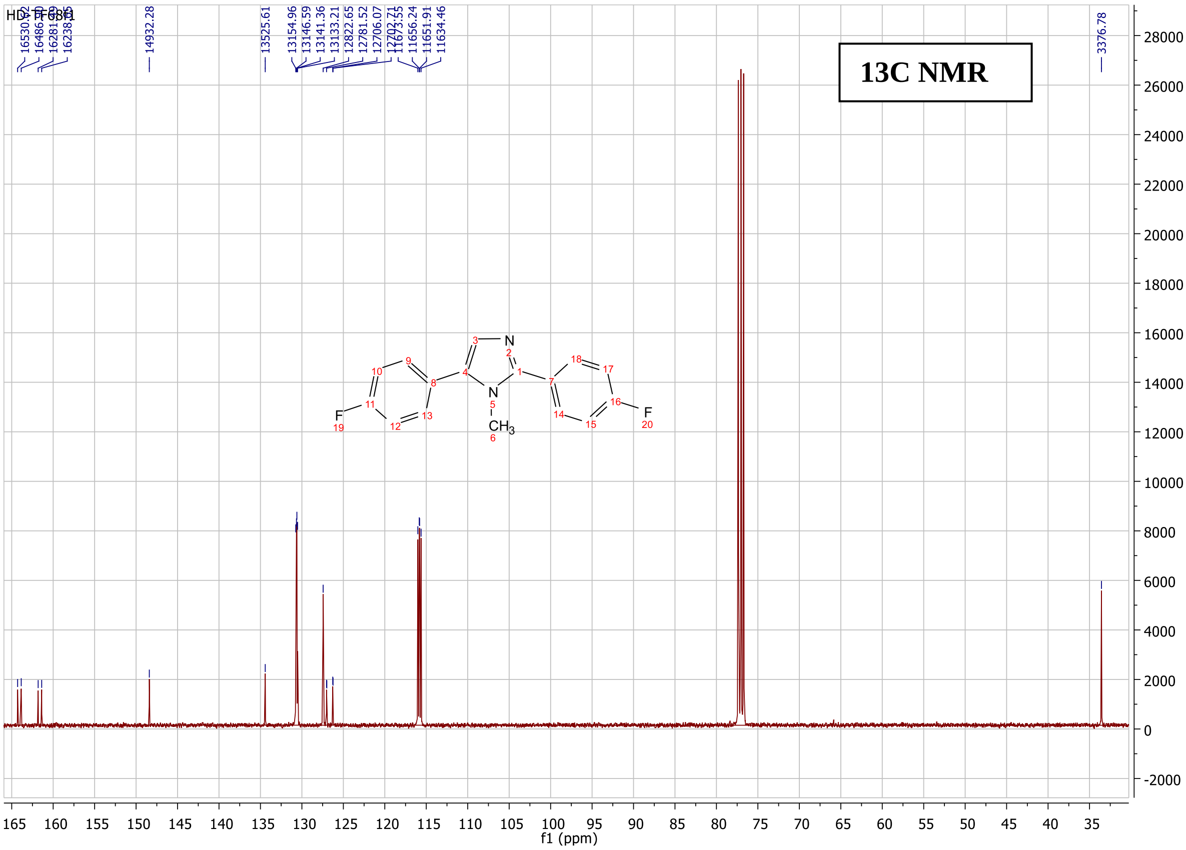

Compound 15

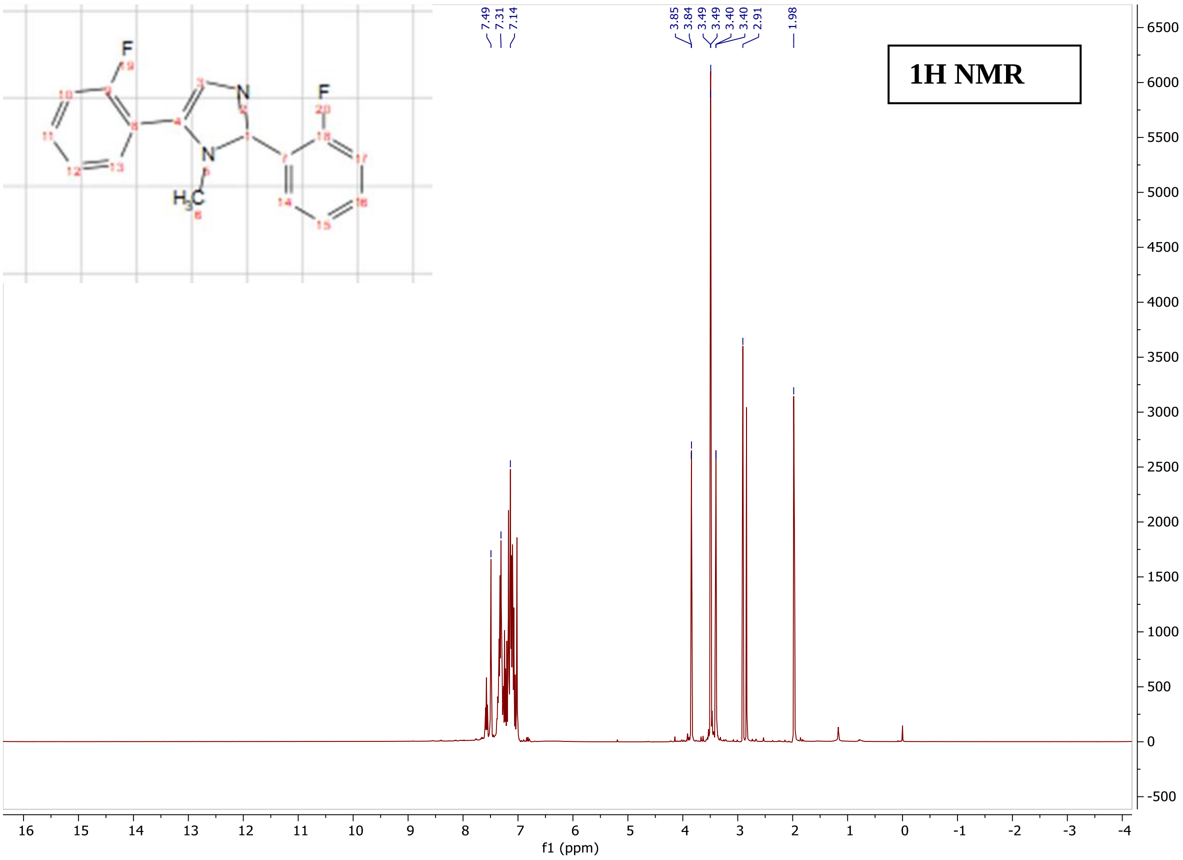

1H NMR
7.49
7.31
7.14
3.85
3.84
3.49
3.49
3.40
3.40
2.91
1.98
f1 (ppm)

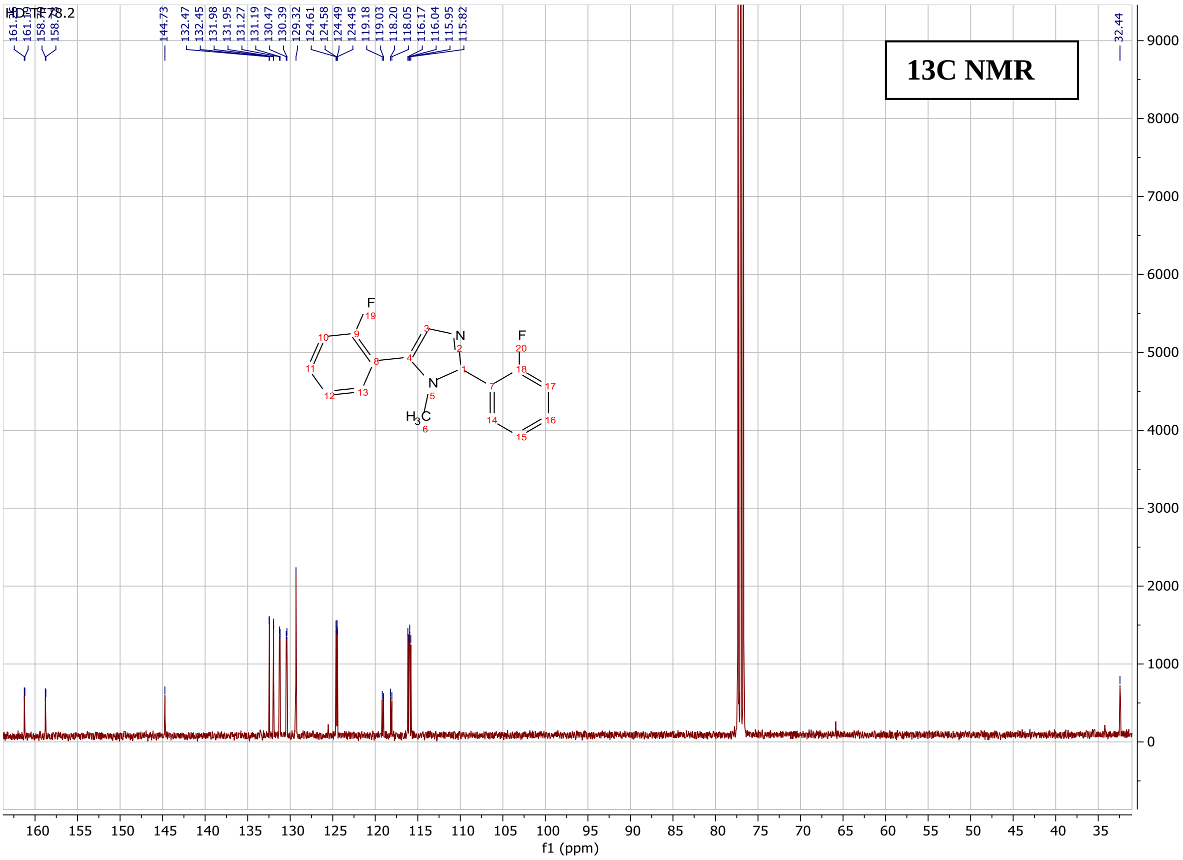

13C NMR
144.73
132.47
132.45
131.98
131.95
131.27
131.19
130.47
130.39
129.32
124.61
124.58
124.49
124.45
119.18
119.03
118.20
118.05
116.17
116.04
115.95
115.82
32.44
f1 (ppm)

Compound 16

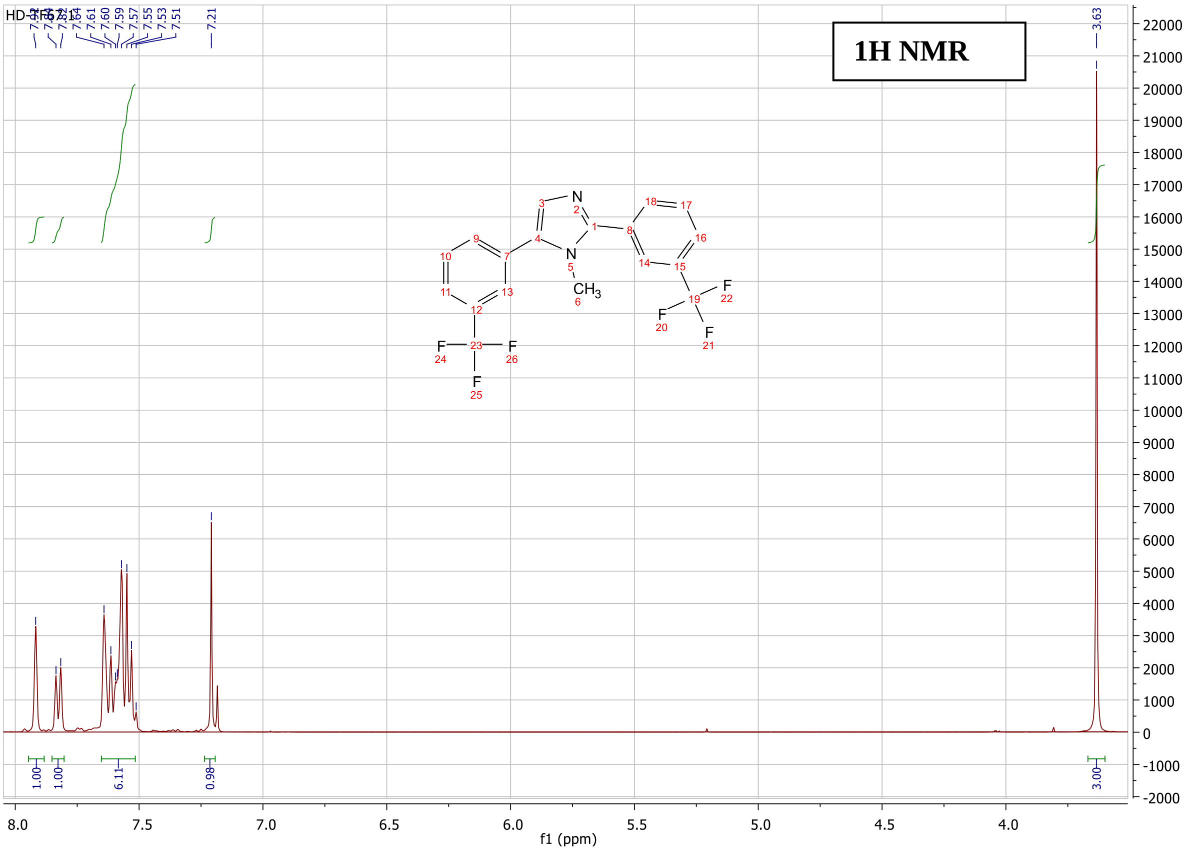

1H NMR
7.61
7.60
7.59
7.57
7.55
7.53
7.51
7.21
3.63
1.00
1.00
6.11
0.98
3.00
f1 (ppm)

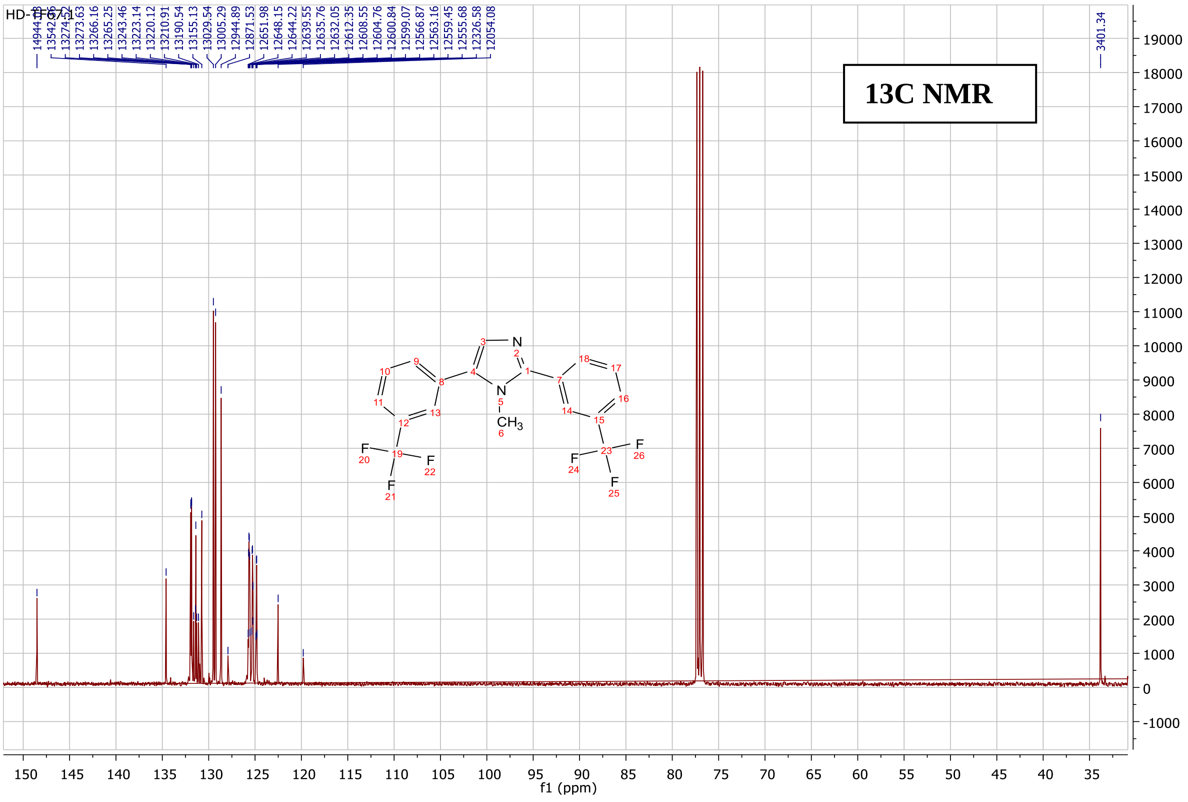

13C NMR
13273.63
13266.16
13265.25
13243.46
13223.14
13220.12
13210.91
13190.54
13155.13
13029.54
13005.29
12944.89
12871.53
12651.98
12648.15
12644.22
12639.55
12635.76
12632.05
12612.35
12608.55
12604.76
12600.84
12599.07
12566.87
12563.16
12559.45
12555.68
12326.58
12054.08
3401.34
f1 (ppm)

Compound 17

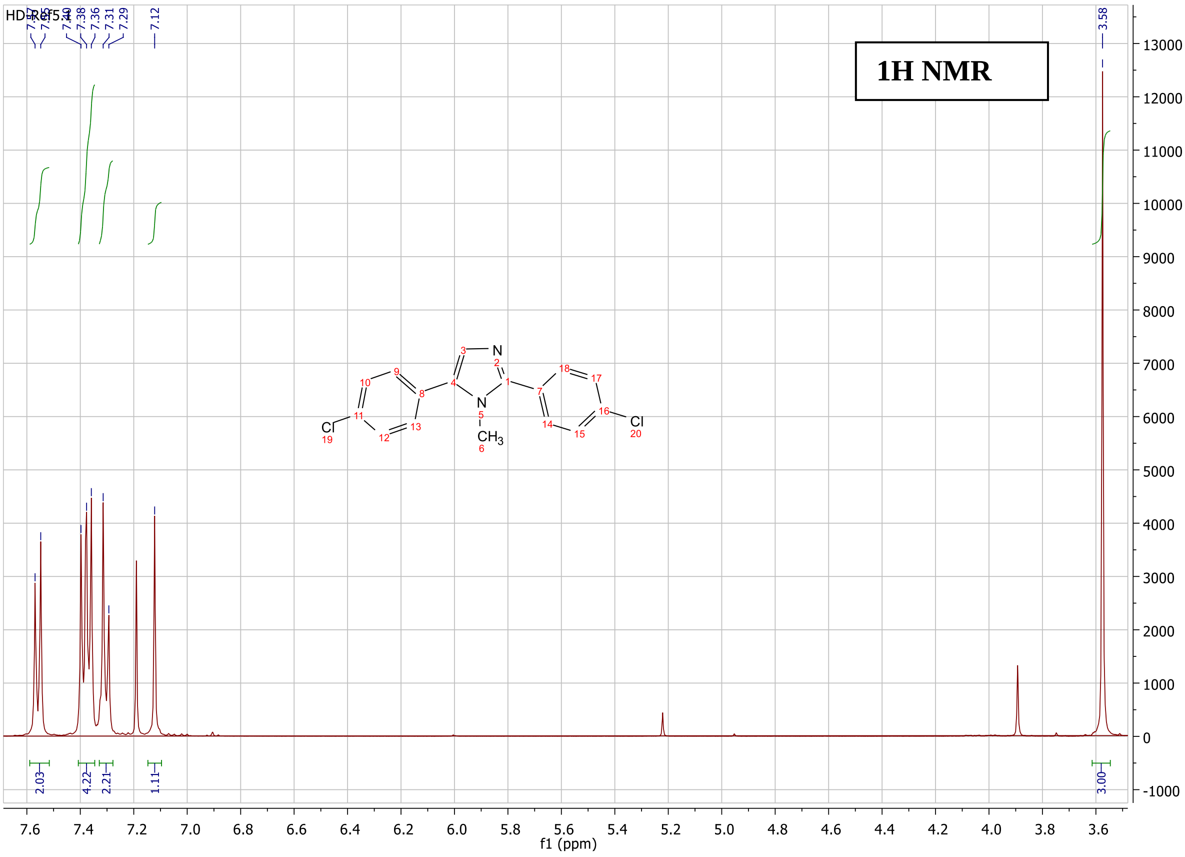
1H NMR
7.38
7.36
7.31
7.29
7.12
3.58
2.03
4.22
2.21
1.11
3.00
f1 (ppm)

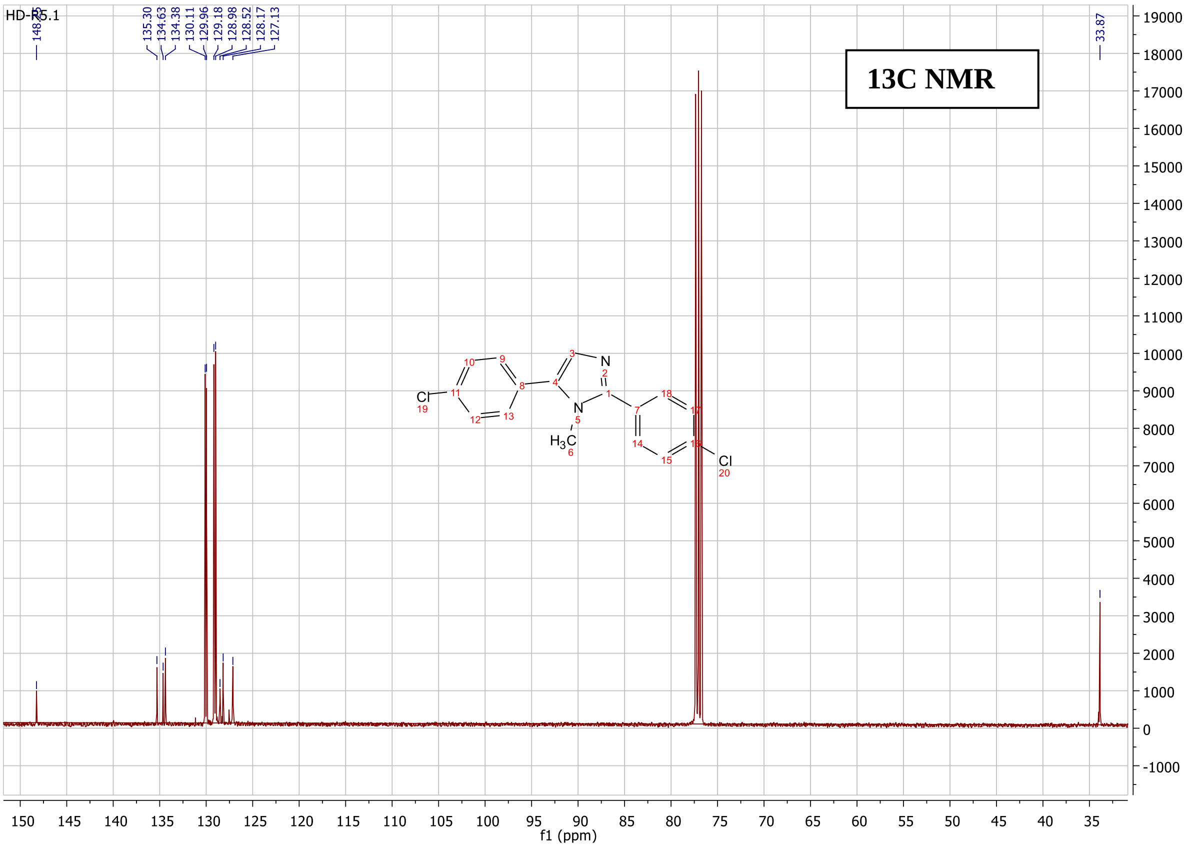
HD-R5.1
13C NMR
135.30
134.63
134.38
130.11
129.96
129.18
128.98
128.52
128.17
127.13
33.87
f1 (ppm)

Compound 19

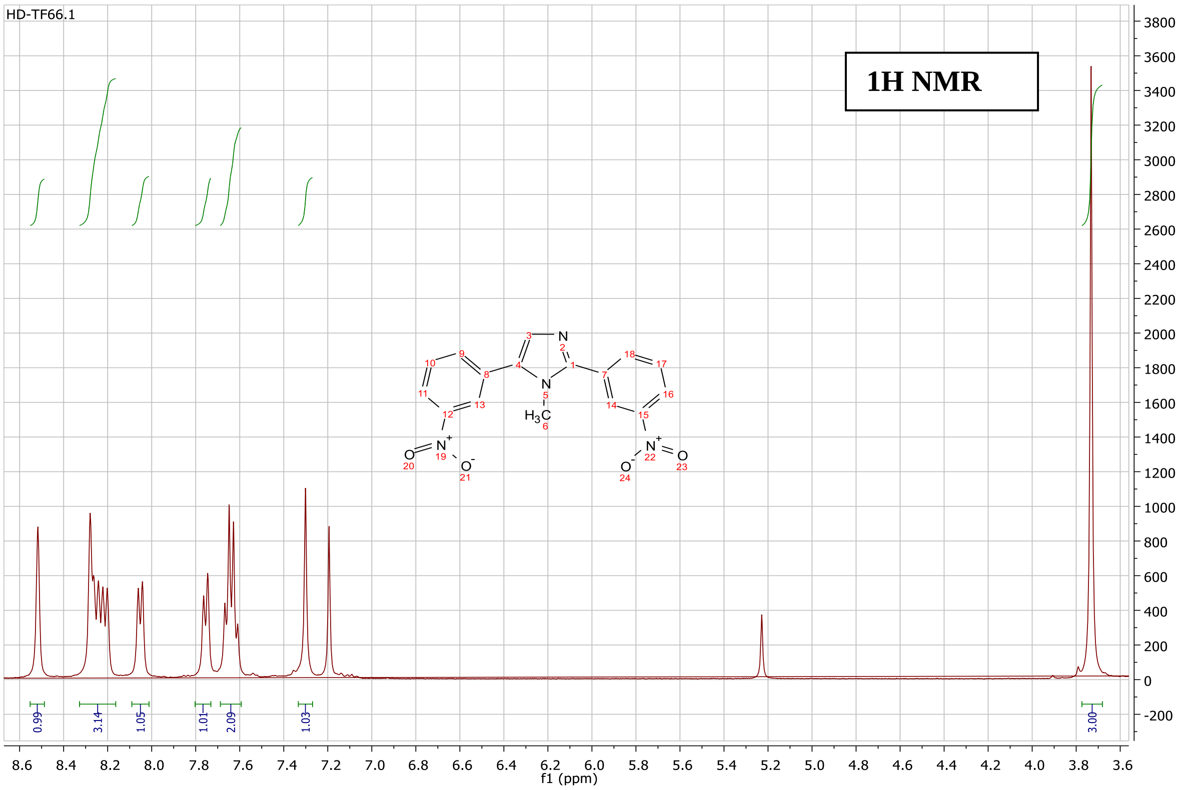

HD-TF66.1
1H NMR
0.99
3.14
1.05
1.01
2.09
1.03
3.00
f1 (ppm)

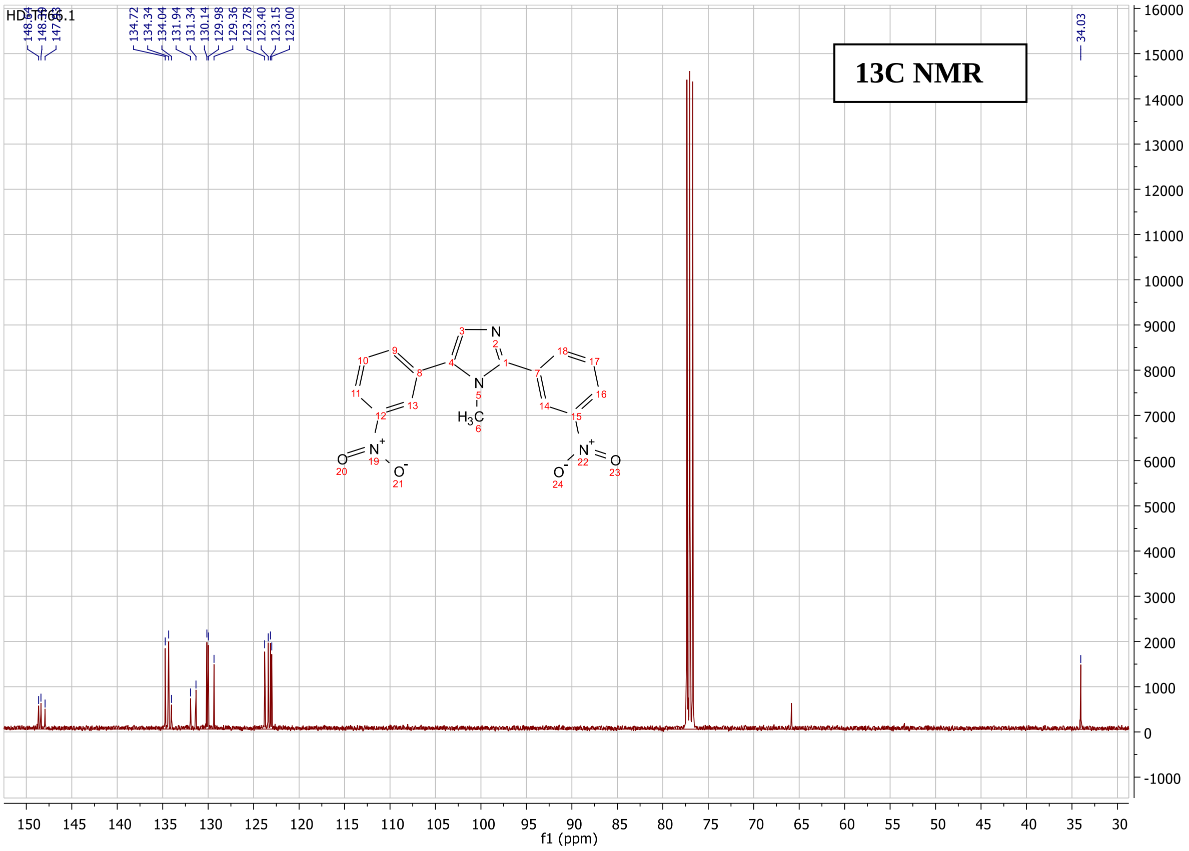

HD-TF66.1
134.72
134.34
134.04
131.94
131.34
130.14
129.98
129.36
123.78
123.40
123.15
123.00
34.03
13C NMR
f1 (ppm)

Compound 20

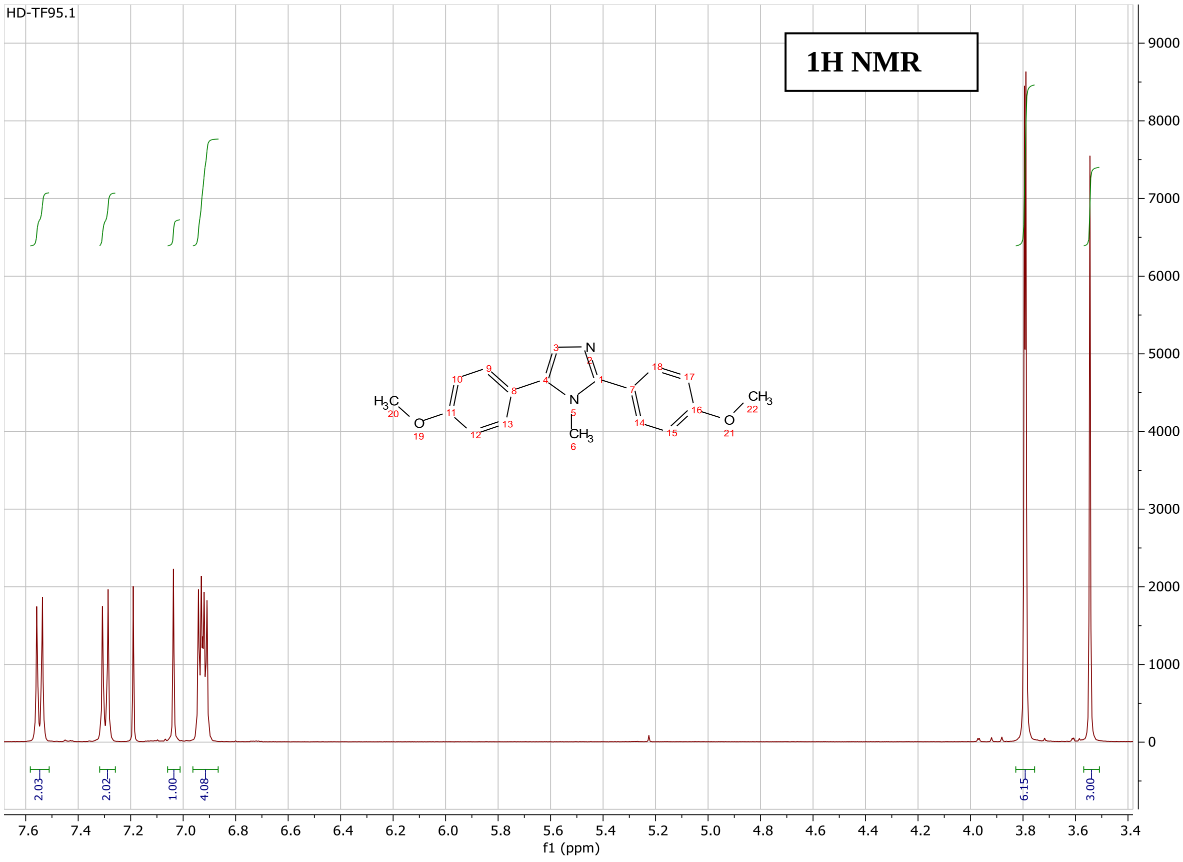

HD-TF95.1
1H NMR
2.03
2.02
1.00
4.08
6.15
3.00
f1 (ppm)

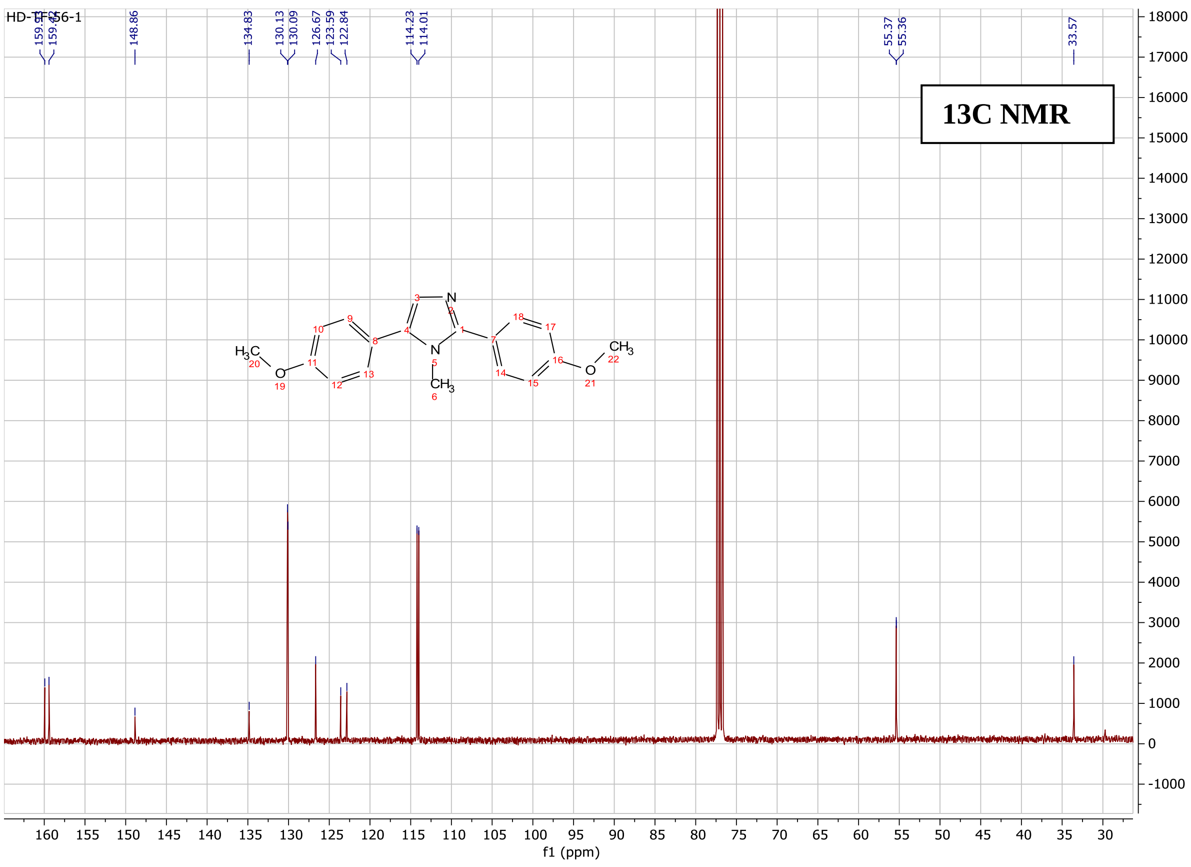

HD-TF56-1
148.86
134.83
130.13
130.09
126.67
123.59
122.84
114.23
114.01
55.37
55.36
33.57
13C NMR
f1 (ppm)

Compound 21

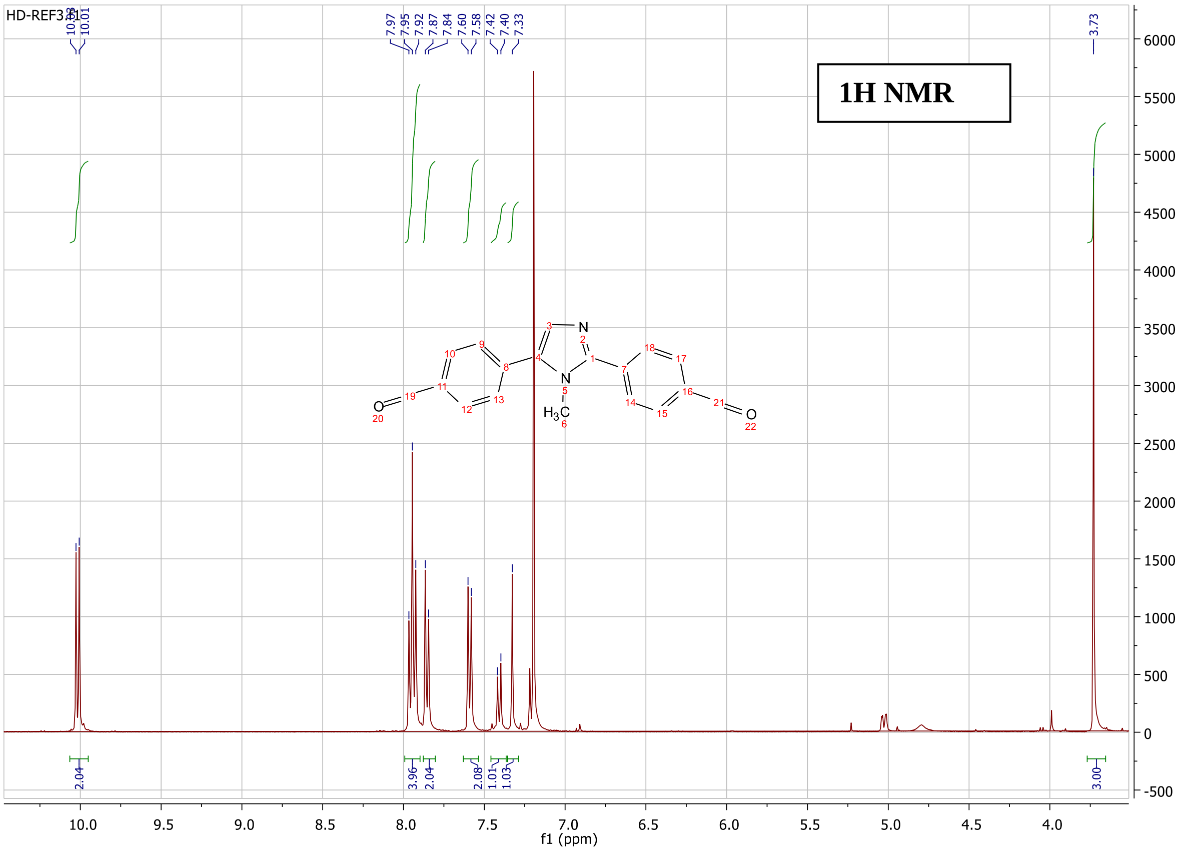

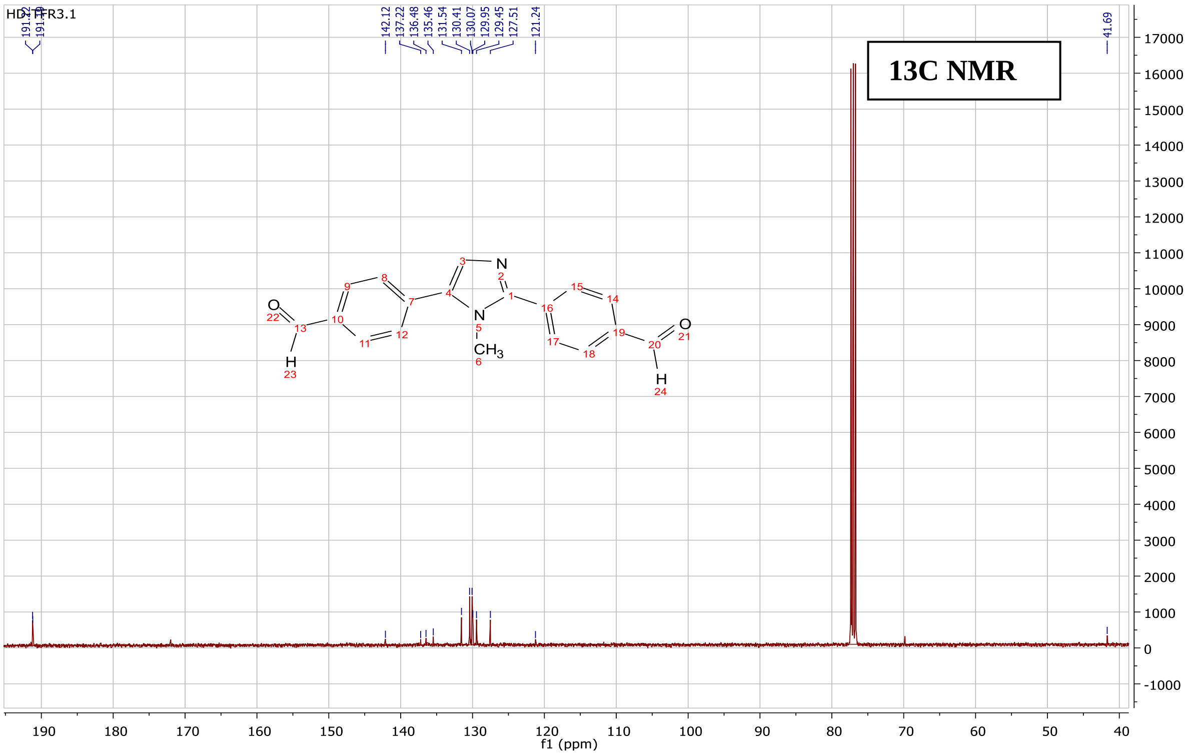

Compound 22

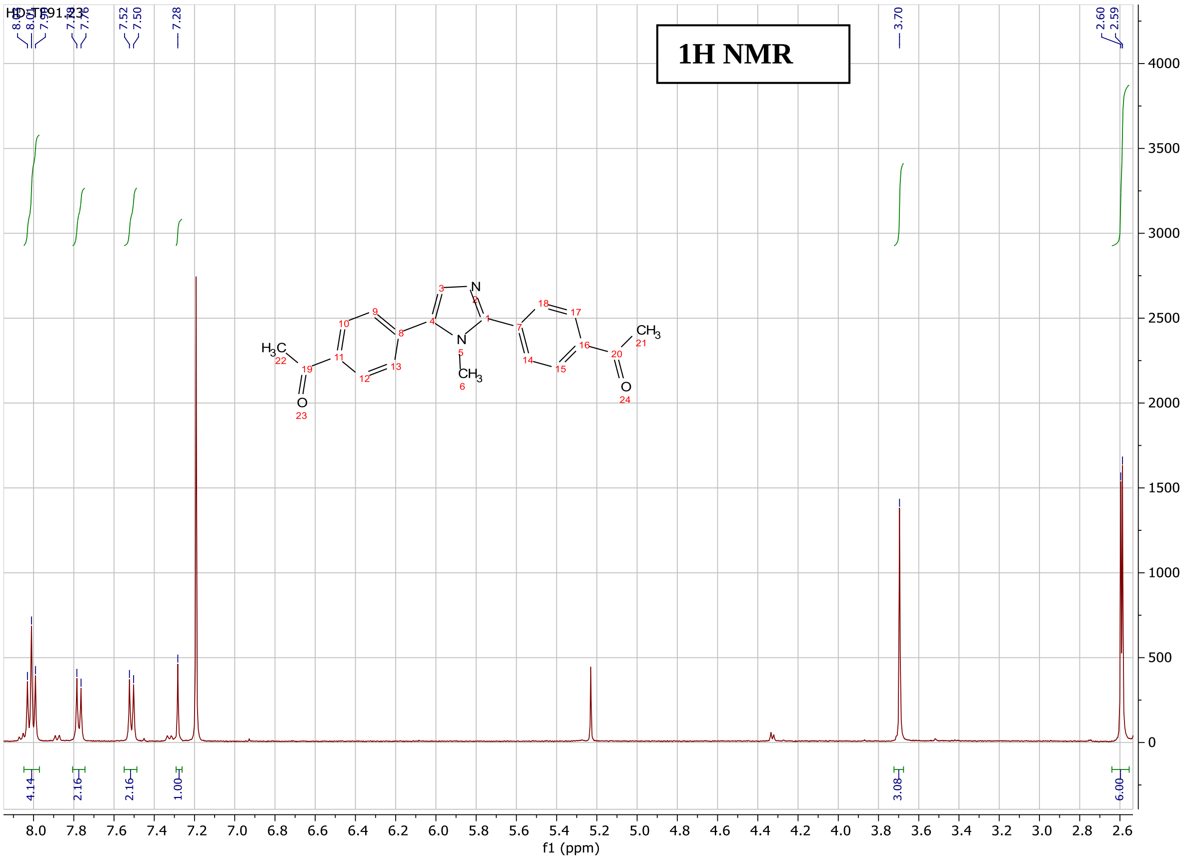
1H NMR
HD-TF91.23
7.52
7.50
7.28
3.70
2.60
2.59
4.14
2.16
2.16
1.00
3.08
6.00
f1 (ppm)

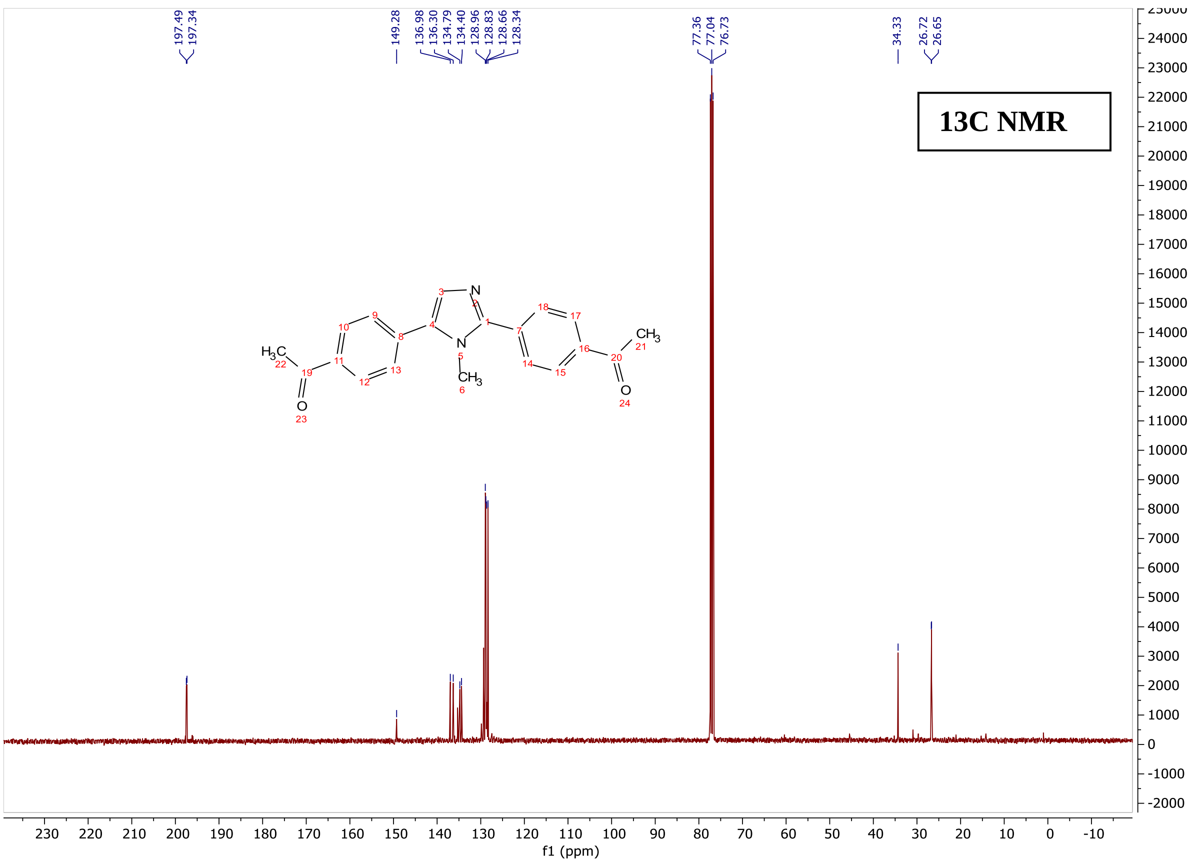
13C NMR
197.49
197.34
149.28
136.98
136.30
134.79
134.40
128.96
128.83
128.66
128.34
77.36
77.04
76.73
34.33
26.72
26.65
f1 (ppm)

Compound 23

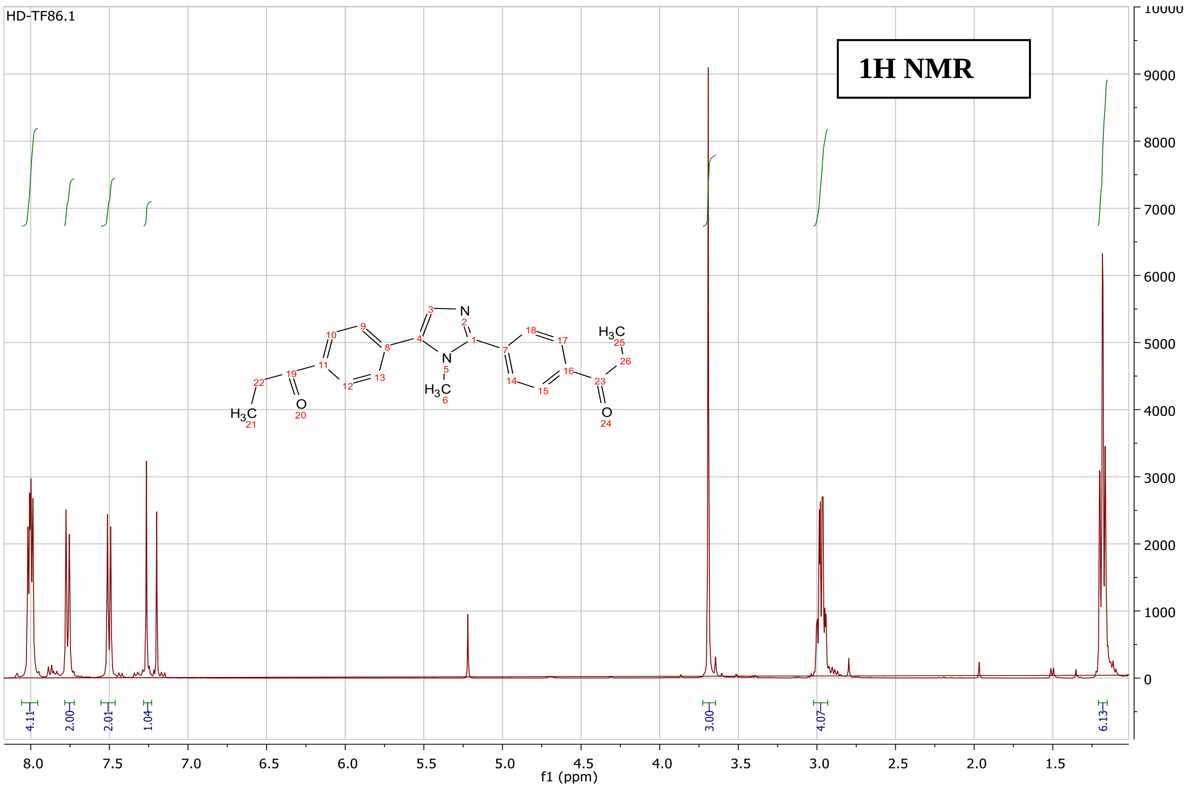
HD-TF86.1
1H NMR
4.11
2.00
2.01
1.04
3.00
4.07
6.13
f1 (ppm)

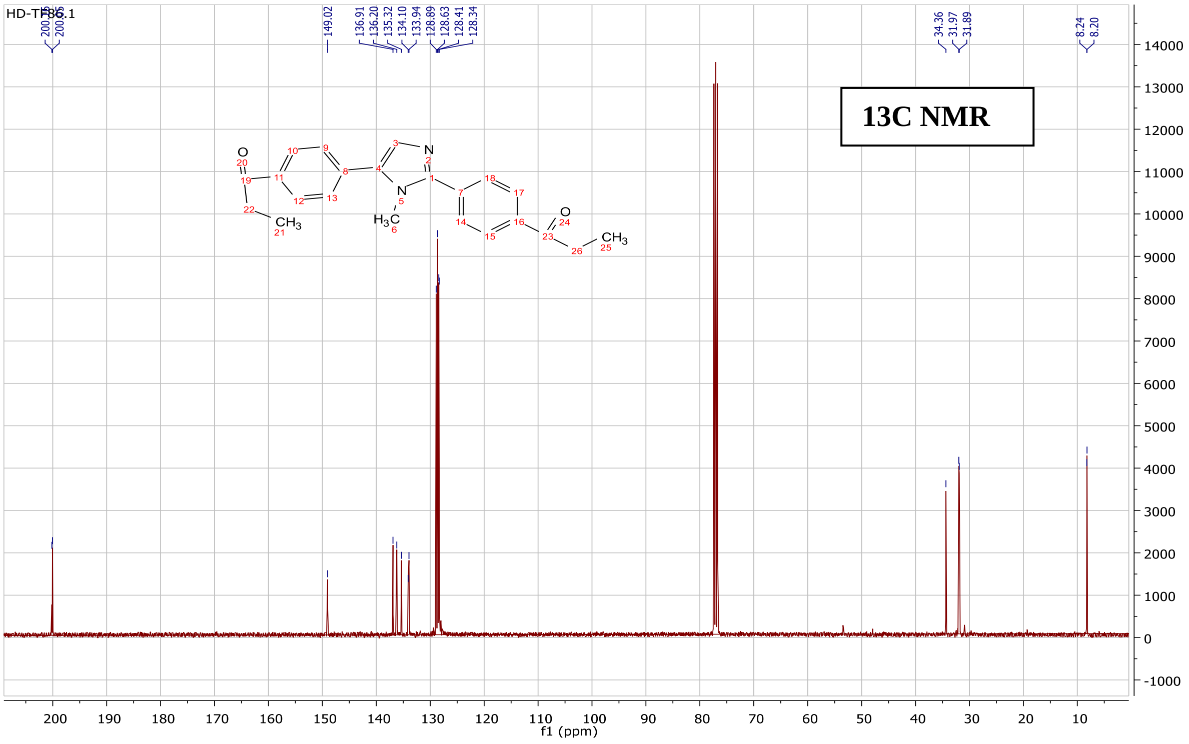
HD-TF86.1
149.02
136.91
136.20
135.32
134.10
133.94
128.89
128.63
128.41
128.34
34.36
31.97
31.89
8.24
8.20
13C NMR
f1 (ppm)

Compound 24

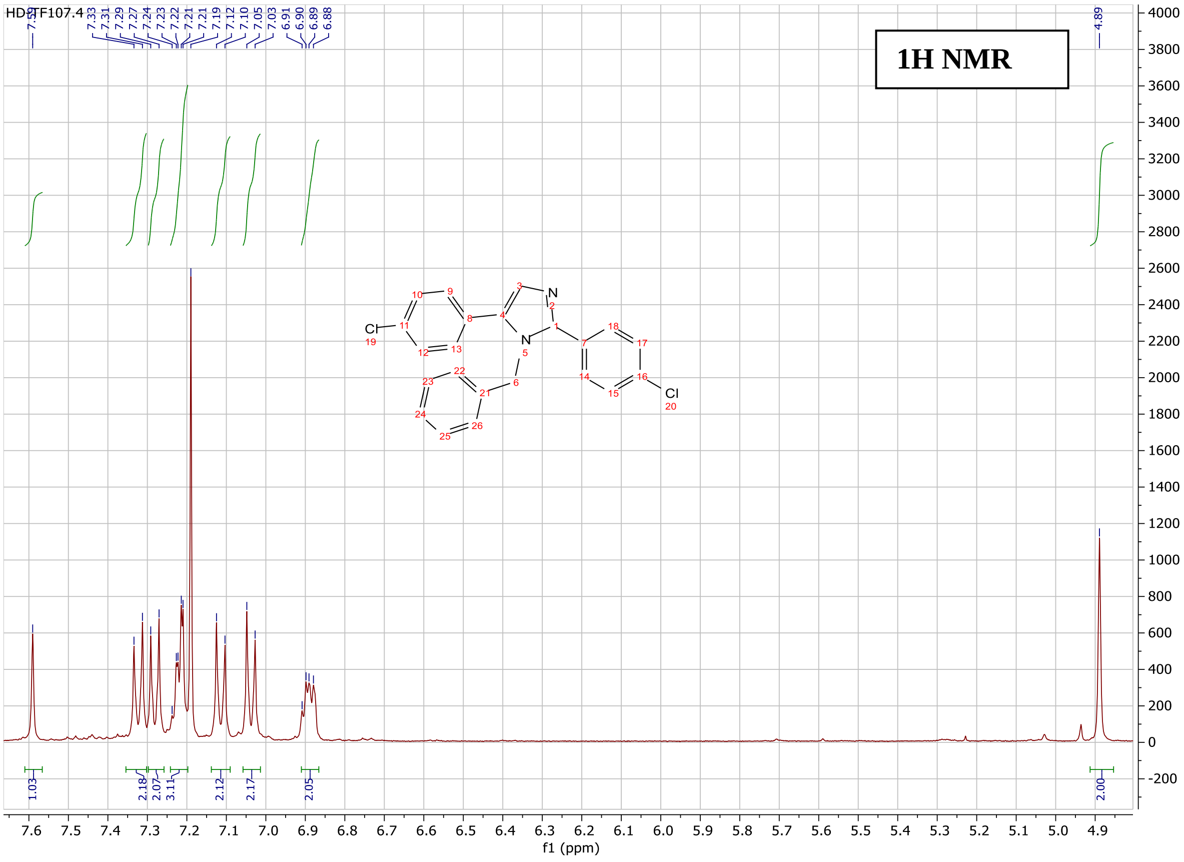

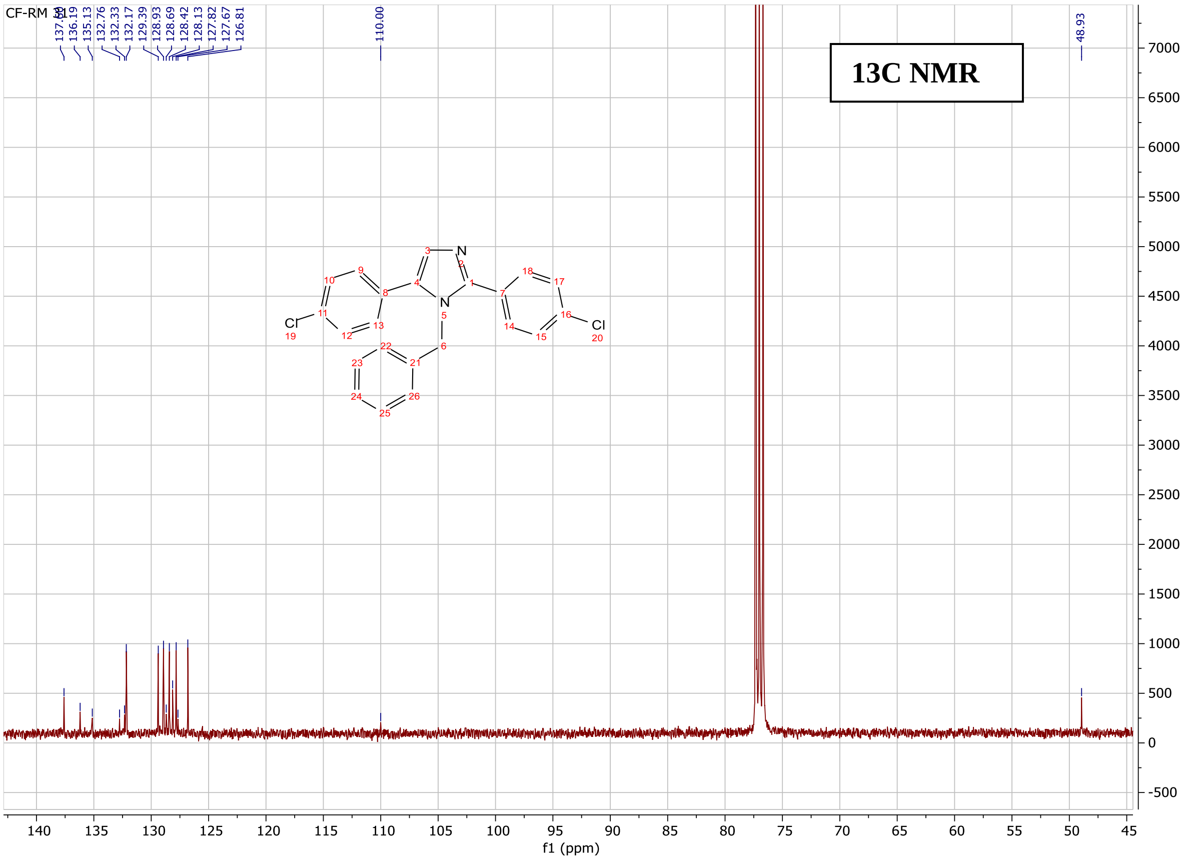

Compound 25

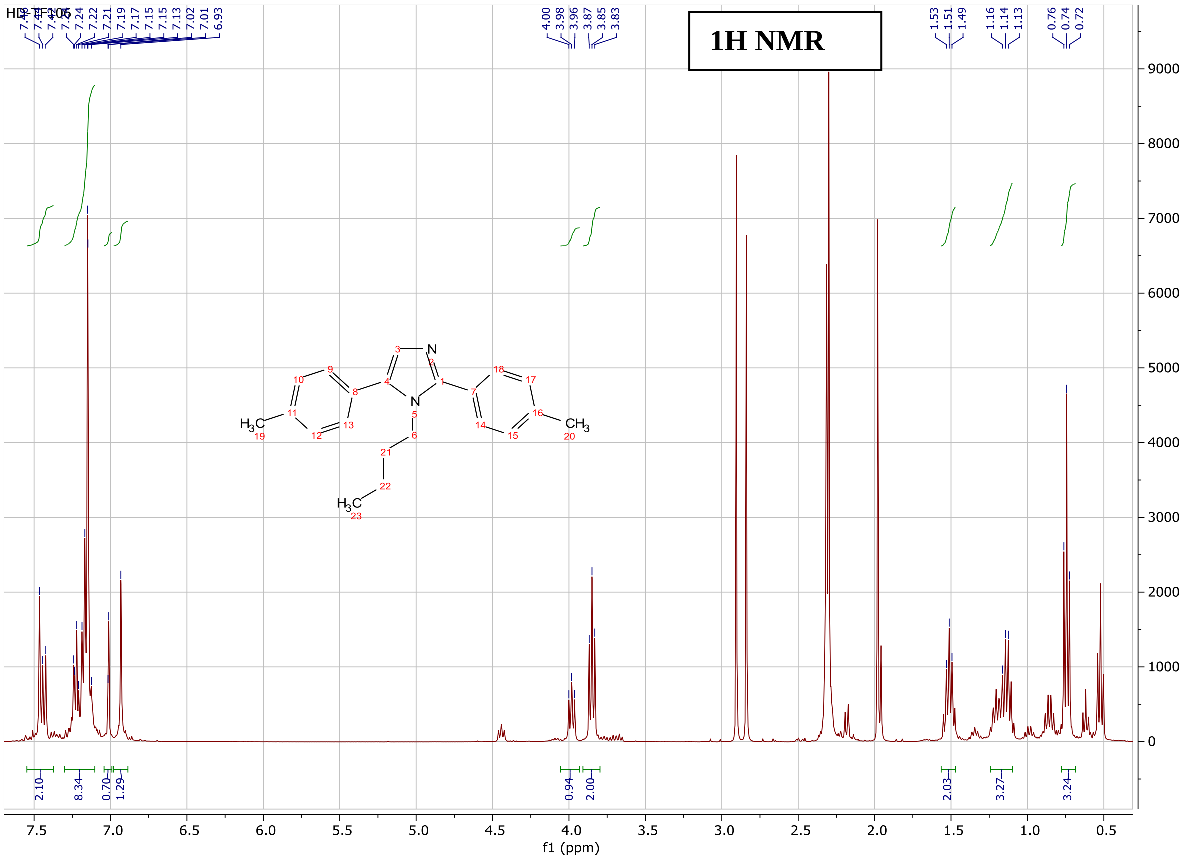

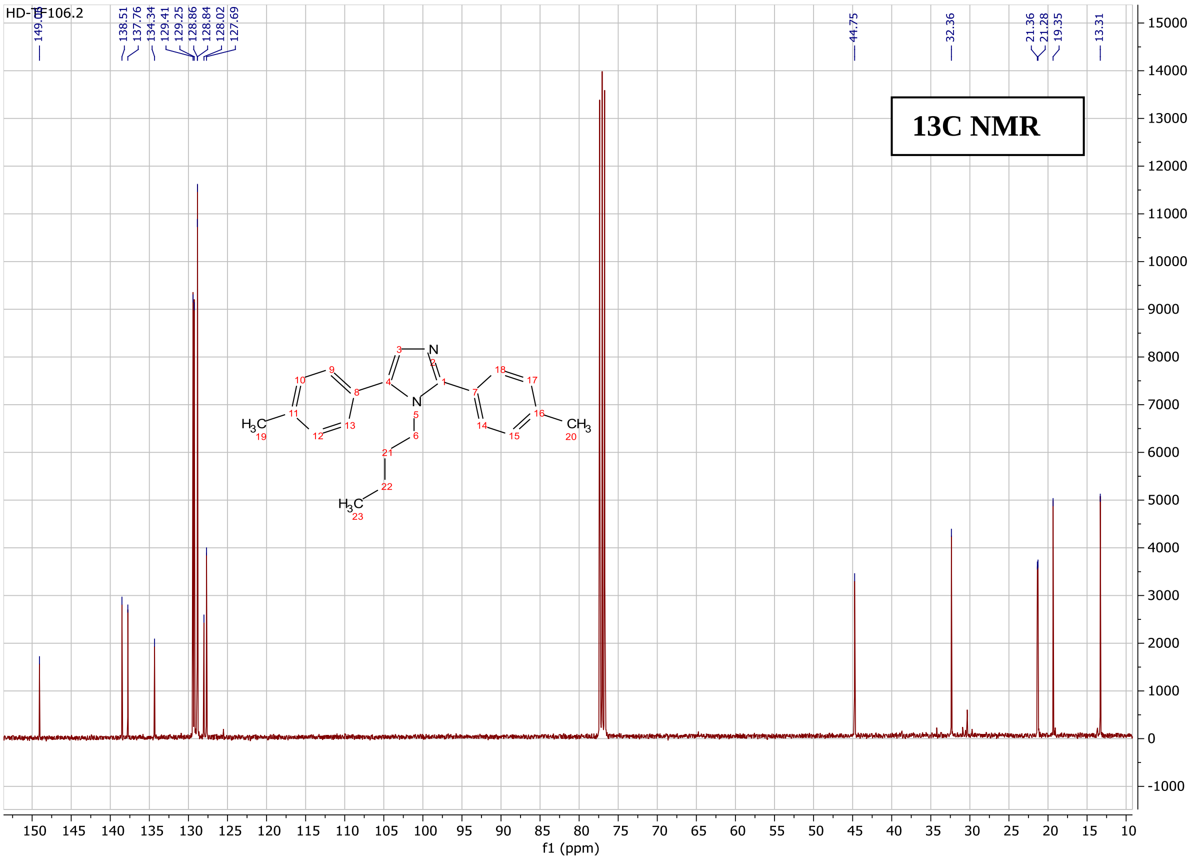

Compound 26

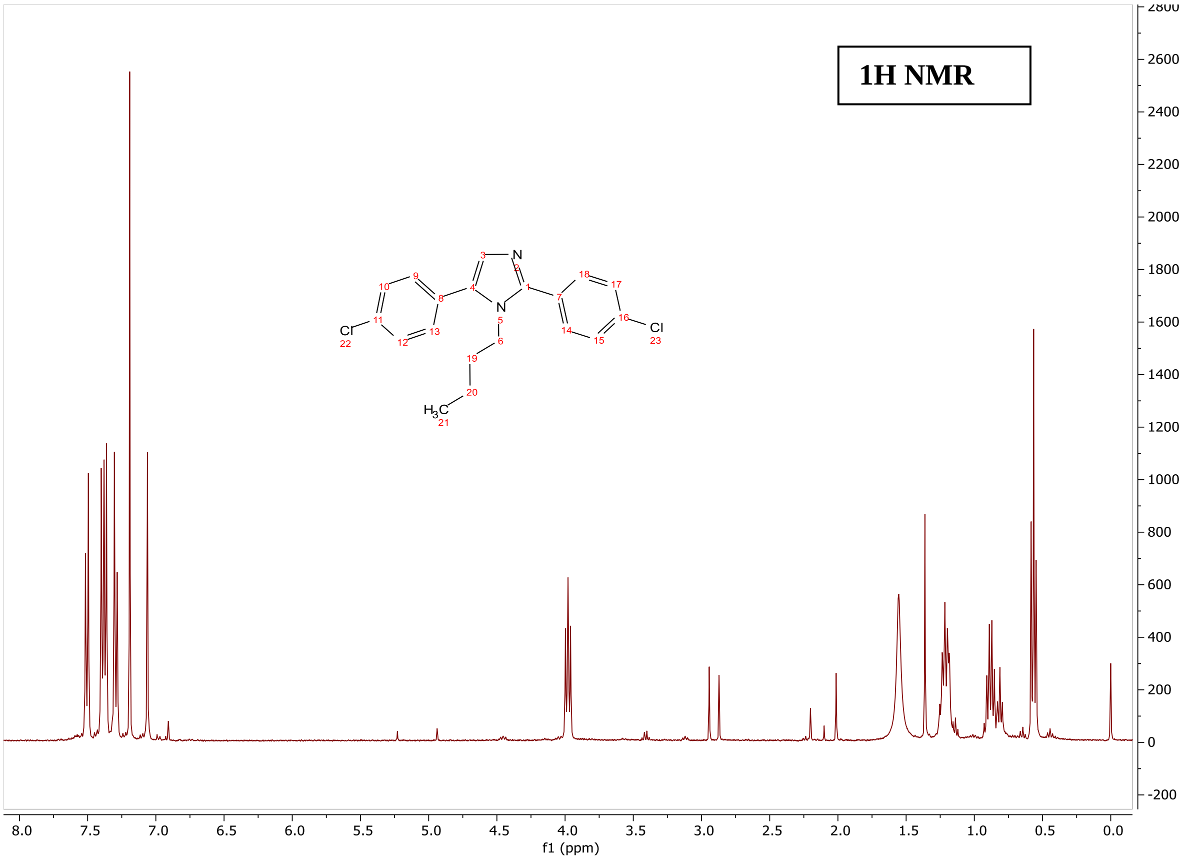

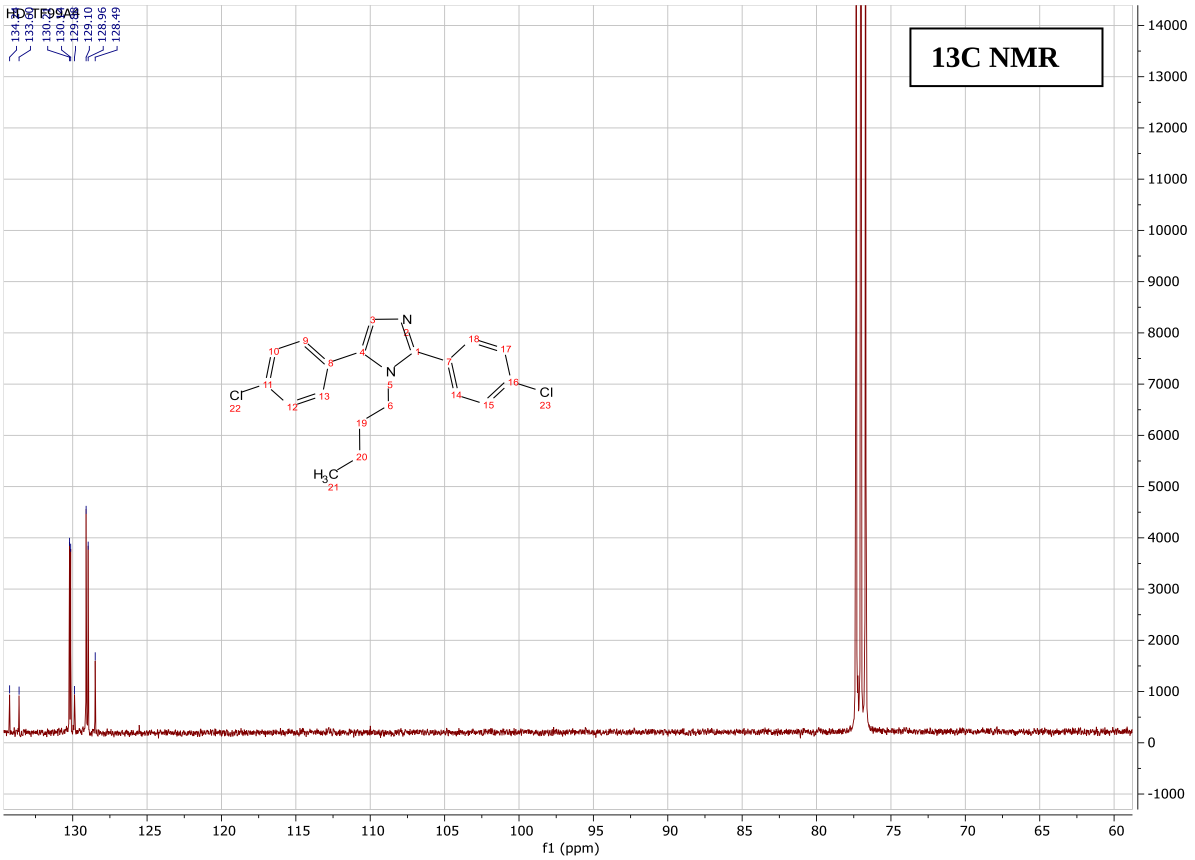

Compound 27

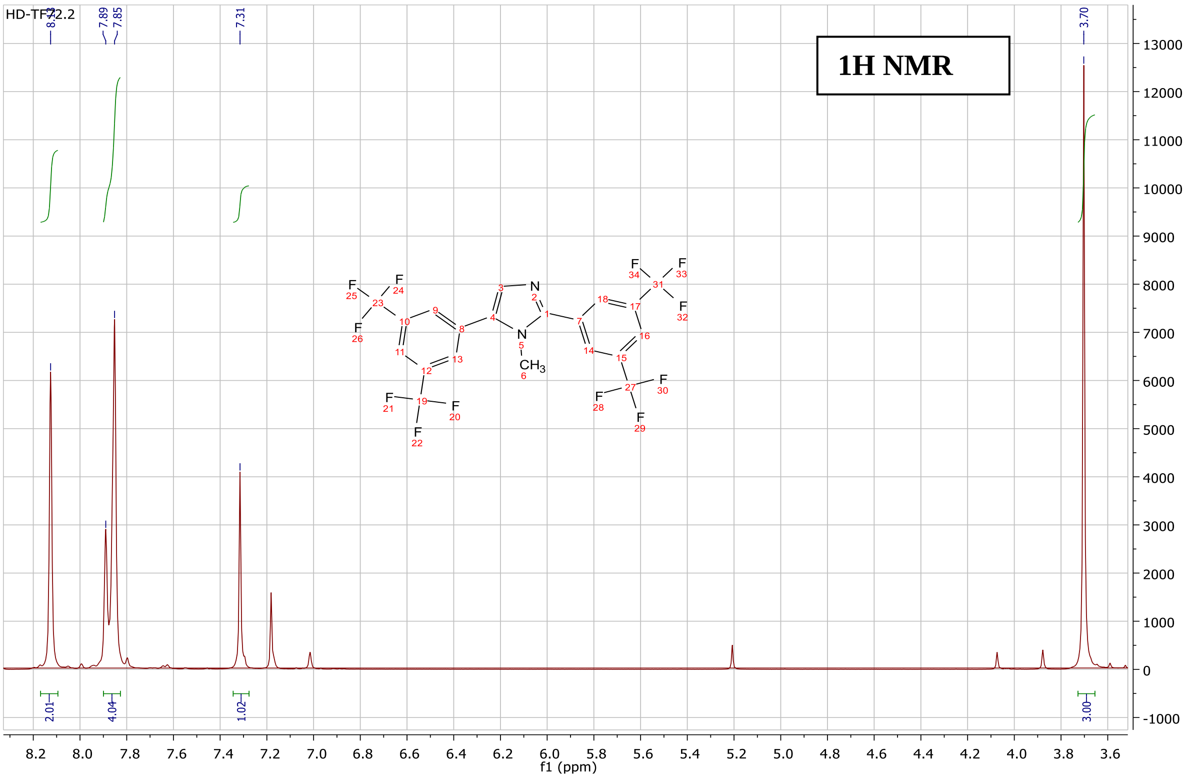
HD-TF72.2
1H NMR
7.89
7.85
7.31
3.70
2.01
4.04
1.02
3.00
f1 (ppm)

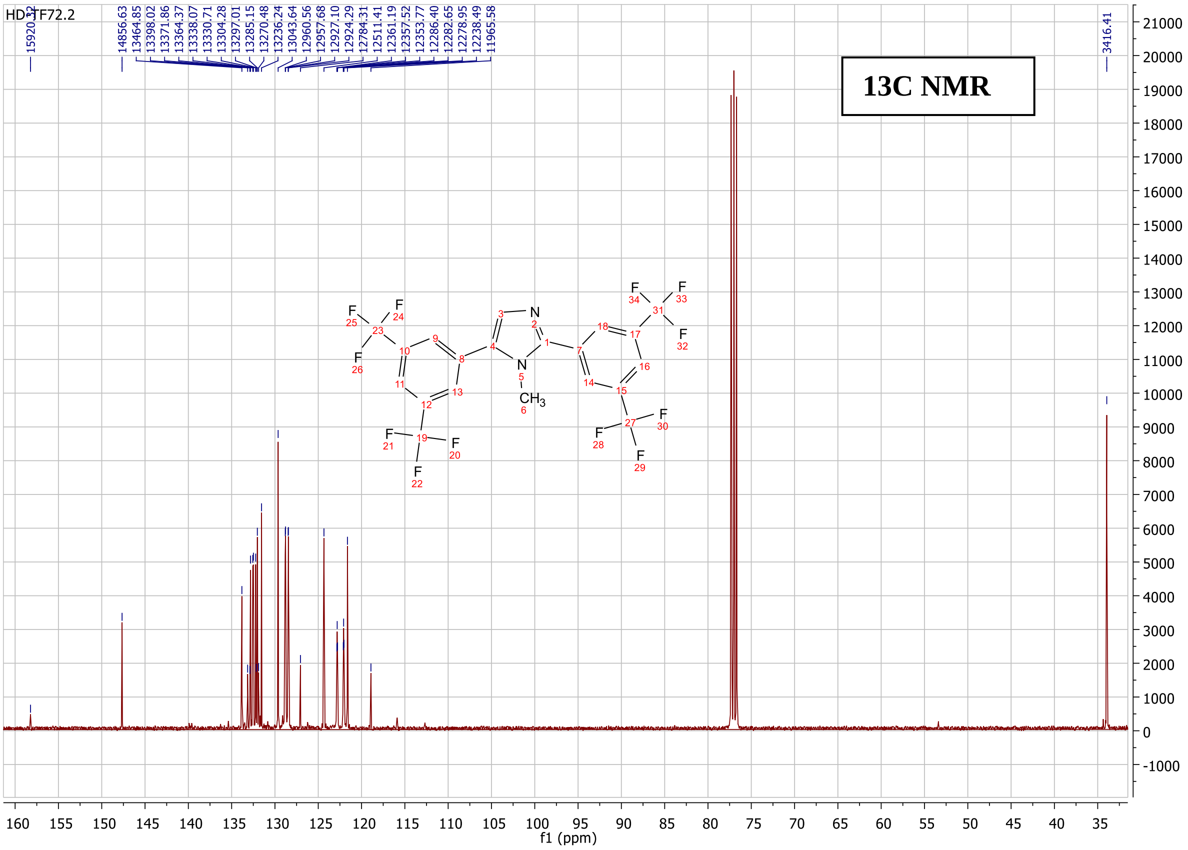
HD-TF72.2
13C NMR
14856.63
13464.85
13398.02
13371.86
13364.37
13338.07
13330.71
13304.28
13297.01
13285.15
13270.48
13236.24
13043.64
12960.56
12957.68
12927.10
12924.29
12784.31
12511.41
12361.19
12357.52
12353.77
12286.40
12282.65
12278.95
12238.49
11965.58
3416.41
f1 (ppm)

Compound 28

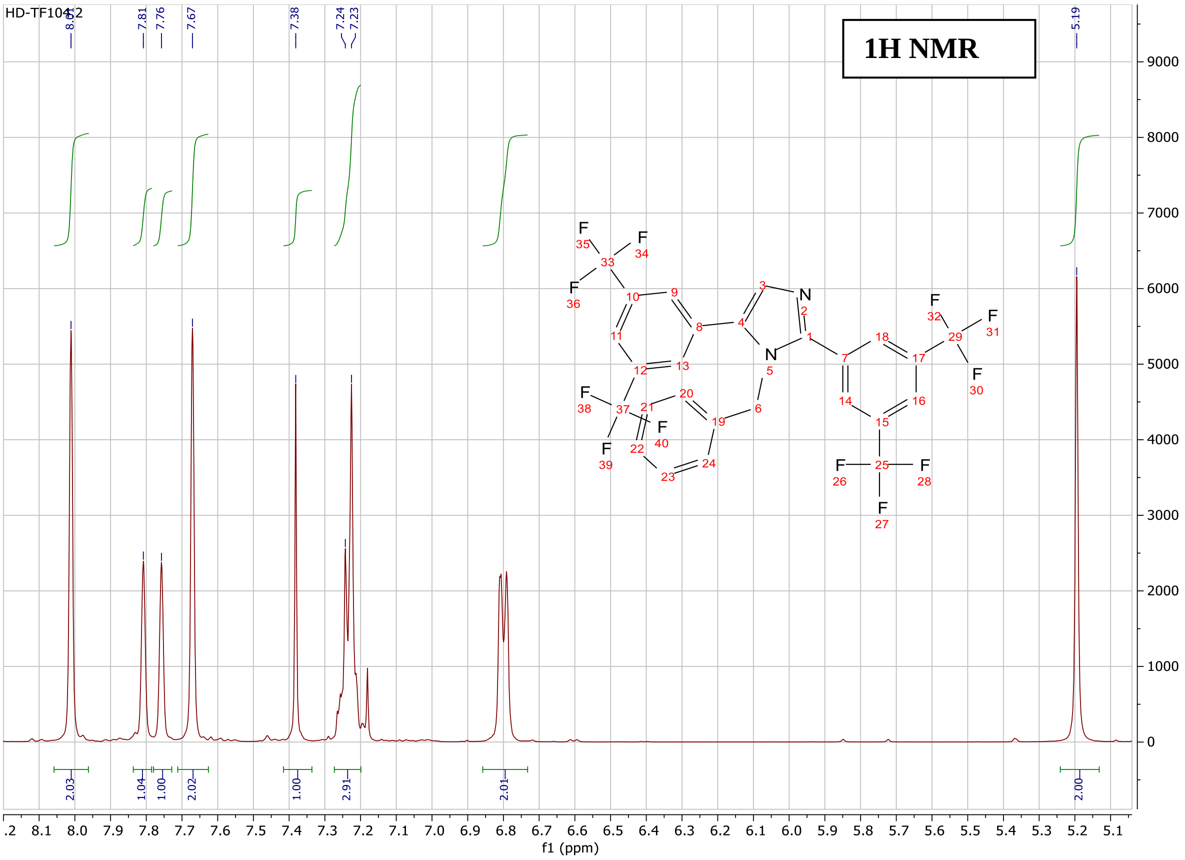
HD-TF104-2
1H NMR
8.01
7.81
7.76
7.67
7.38
7.24
7.23
5.19
2.03
1.04
1.00
2.02
1.00
2.91
2.01
2.00
f1 (ppm)

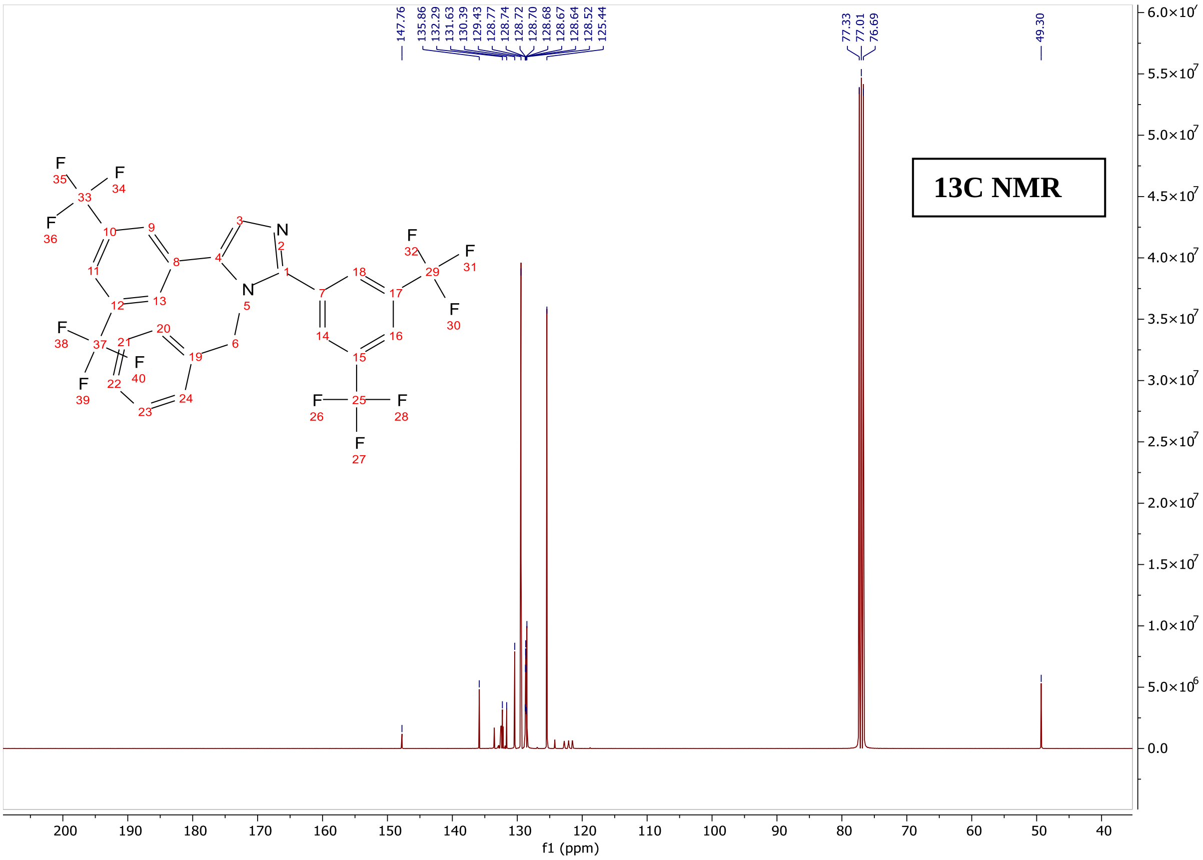
13C NMR
147.76
135.86
132.29
131.63
130.39
129.43
128.77
128.74
128.72
128.70
128.68
128.67
128.64
128.52
125.44
77.33
77.01
76.69
49.30
f1 (ppm)

Compound 31

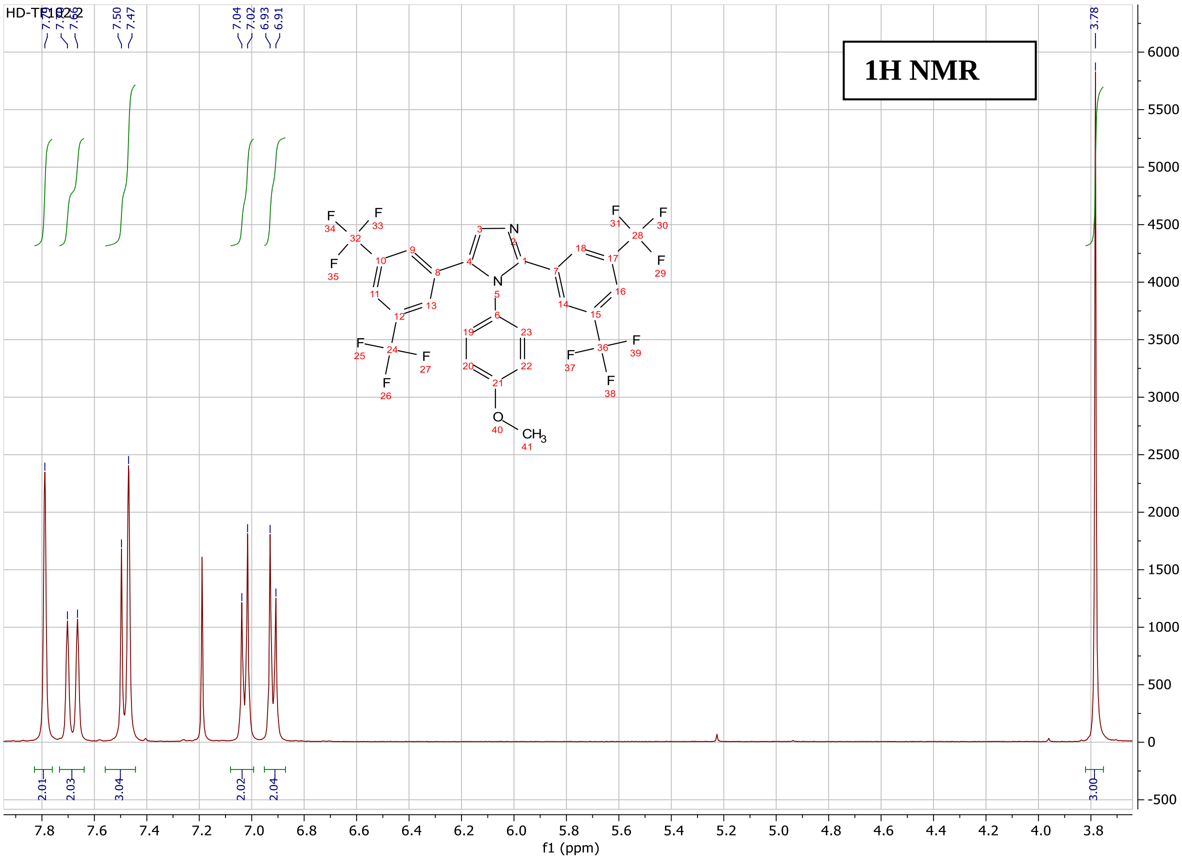
1H NMR
7.50
7.47
7.04
7.02
6.93
6.91
3.78
2.01
2.03
3.04
2.02
2.04
3.00
f1 (ppm)

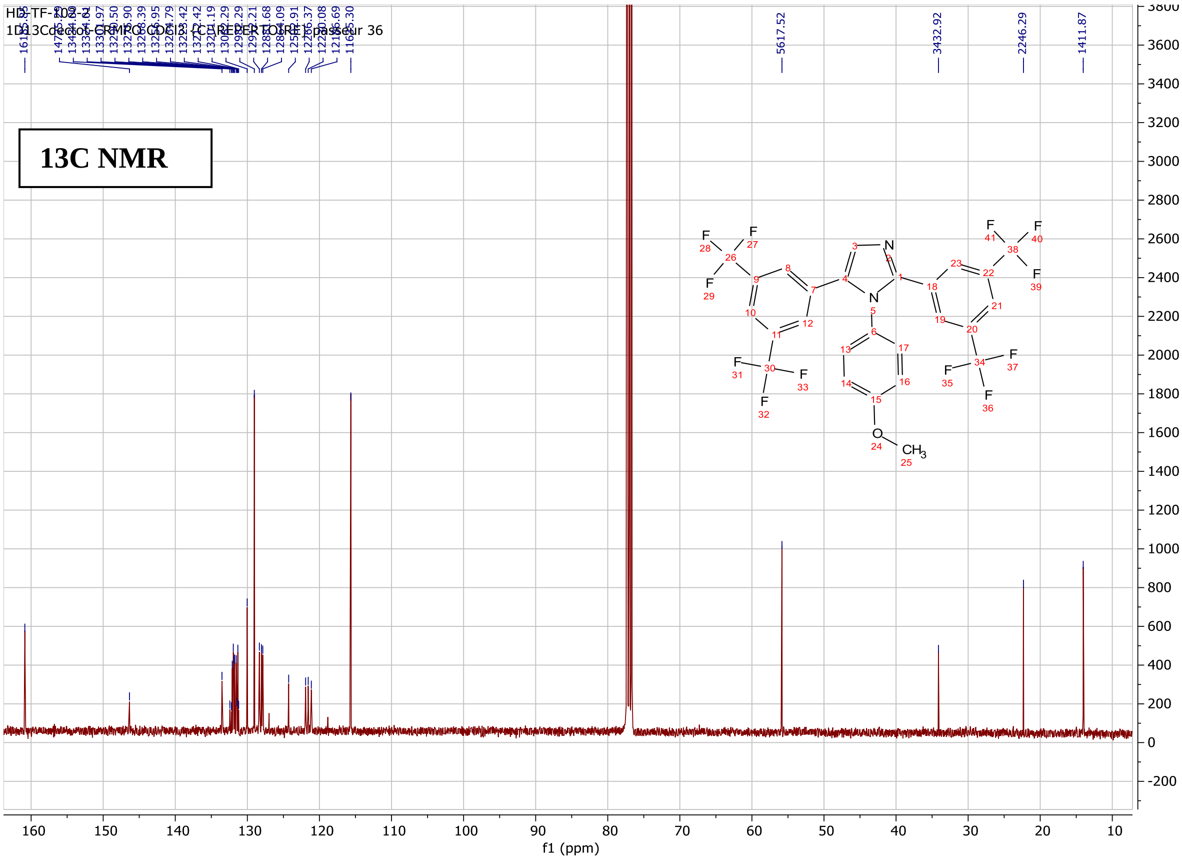
13C NMR
5617.52
3432.92
2246.29
1411.87
f1 (ppm)

Compound 32

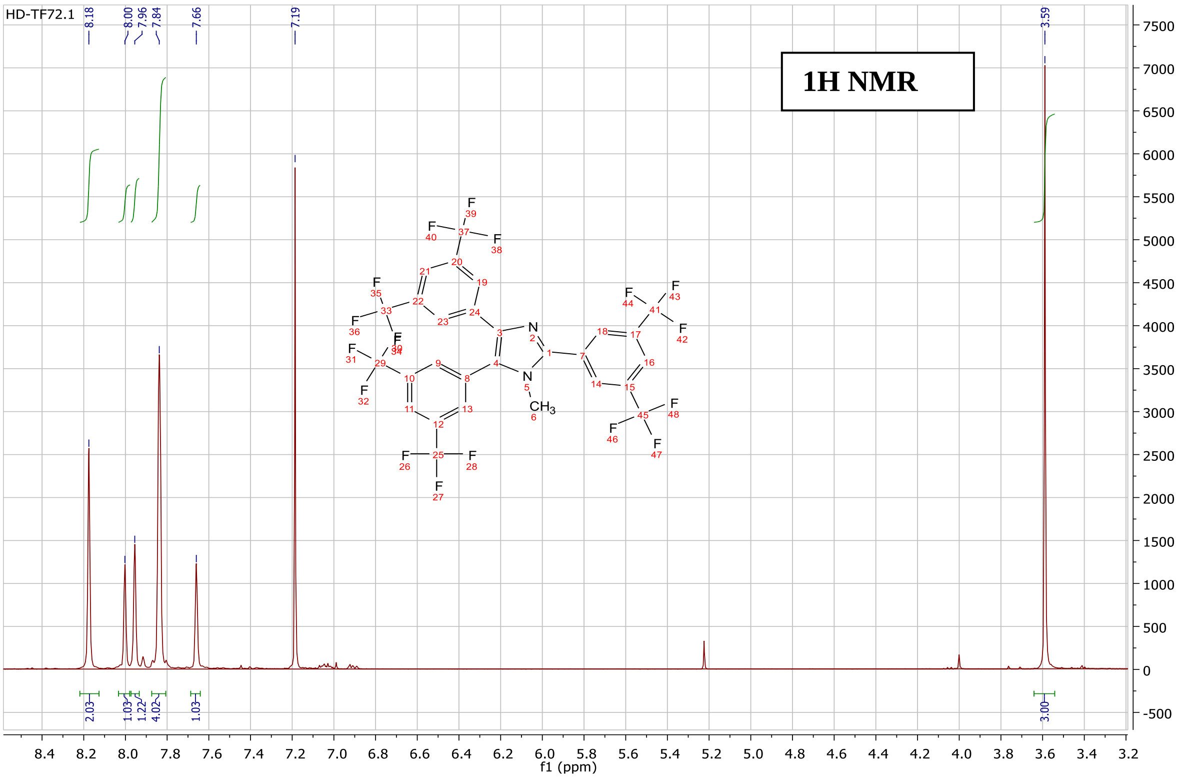

HD-TF72.1
1H NMR
8.18
8.00
7.96
7.84
7.66
7.19
3.59
2.03
1.03
1.22
4.02
1.03
3.00
f1 (ppm)

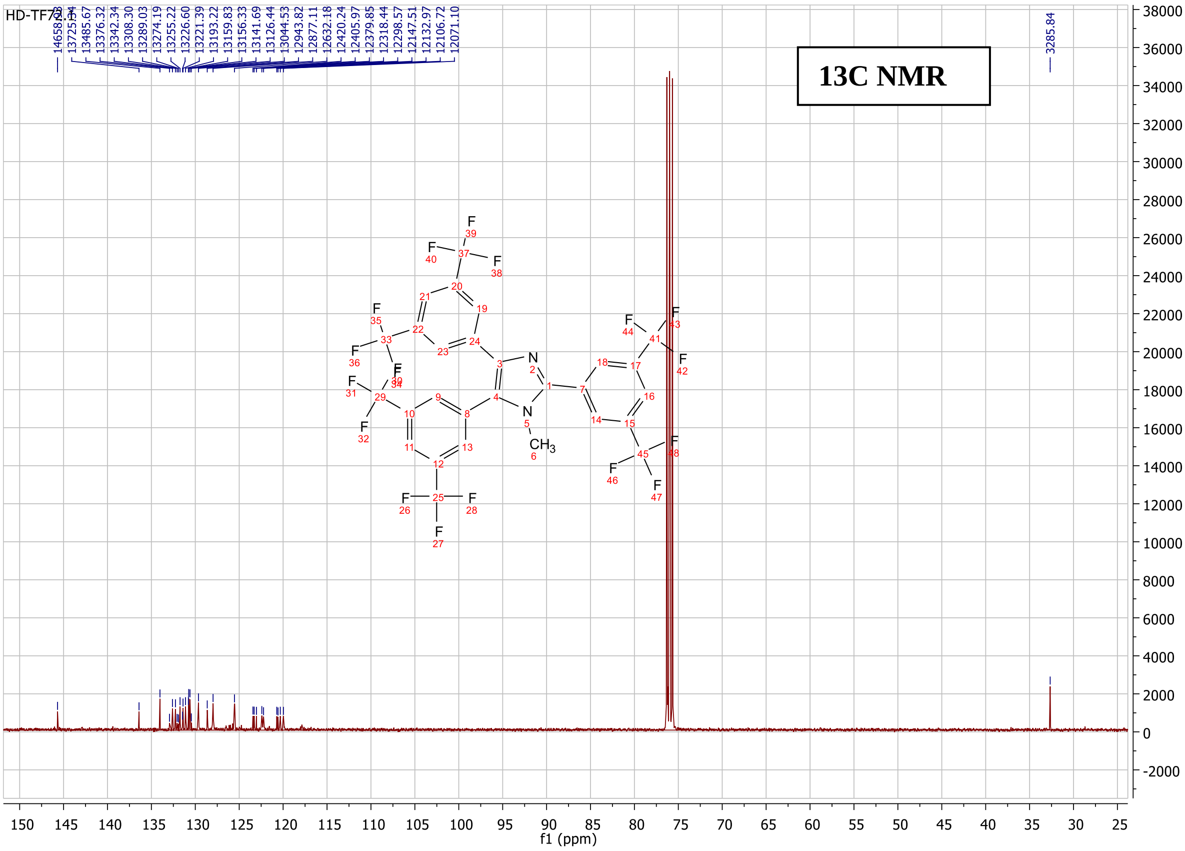

HD-TF72.1
13C NMR
3285.84
f1 (ppm)

Compound 34

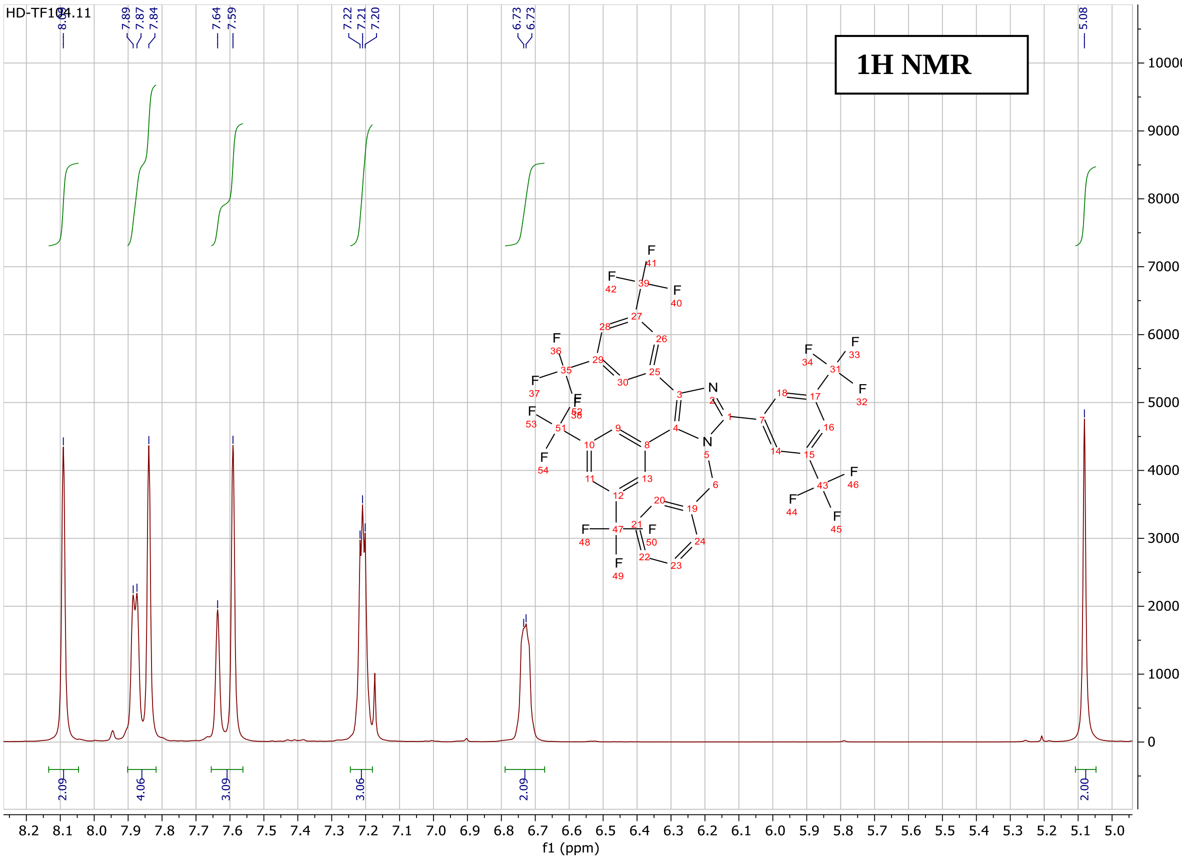

HD-TF104.11
1H NMR
8.10
7.89
7.87
7.84
7.64
7.59
7.22
7.21
7.20
6.73
6.73
5.08
2.09
4.06
3.09
3.06
2.09
2.00
f1 (ppm)

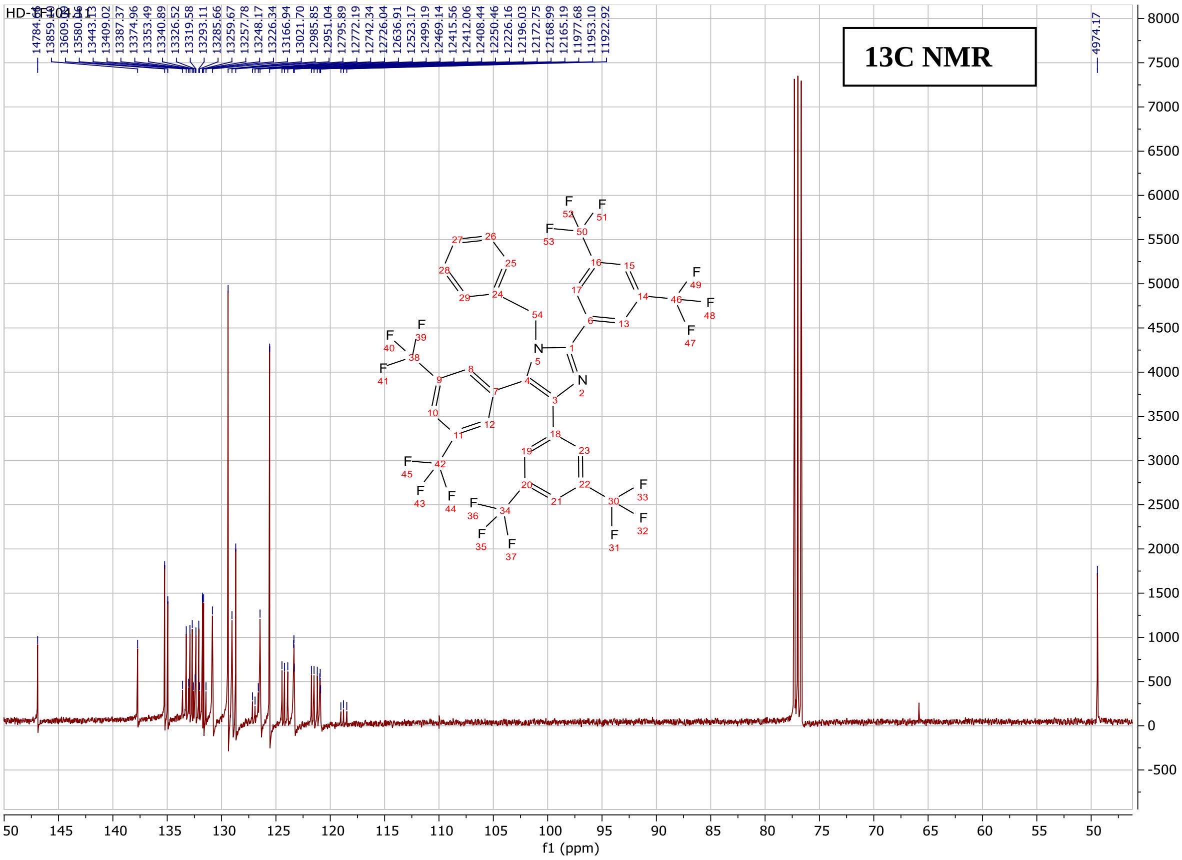

13C NMR
4974.17
f1 (ppm)
